# A novel single‐cell NAD‐ME C_4_ subtype integrated with CAM and bicarbonate use in an aquatic plant

**DOI:** 10.1111/nph.70673

**Published:** 2025-10-21

**Authors:** Hong Sheng Jiang, Wenmin Huang, Shijuan Han, Pengpeng Li, Zuying Liao, Liyuan Wei, Lei Zhao, Shuping Gu, Jun Ding, Brigitte Gontero, Stephen C. Maberly, Wei Li

**Affiliations:** ^1^ State Key Laboratory of Lake and Watershed Science for Water Security Wuhan Botanical Garden, Chinese Academy of Sciences Wuhan 430074 China; ^2^ Hubei Key Laboratory of Wetland Evolution & Ecological Restoration Wuhan Botanical Garden, Chinese Academy of Sciences Wuhan 430074 China; ^3^ Hainan Key Laboratory for Sustainable Utilization of Tropical Bioresources, School of Life Sciences Hainan University HaiKou 570228 China; ^4^ State Key Laboratory of Plant Diversity and Specialty Crops Wuhan Botanical Garden, Chinese Academy of Sciences Wuhan 430074 China; ^5^ Shanghai Sequen Bio‐info Studio Shanghai 200092 China; ^6^ Renmin Hospital of Wuhan University Wuhan University 430072 Wuhan China; ^7^ Aix Marseille Univ, CNRS BIP, UMR 7281, IMM FR 3479 13009 Marseille France; ^8^ UK Centre for Ecology & Hydrology Library Avenue Bailrigg Lancaster LA1 4AP UK; ^9^ School of Ecology and Environment Tibet University Lhasa Xizang 850000 China

**Keywords:** C_4_ photosynthesis, CO_2_ concentrating mechanism, Crassulacean acid metabolism, *Ottelia alismoides*, PEP carboxylase, photosynthesis, Rubisco, single‐cell

## Abstract

- Many plants maximize photosynthesis by using a CO_2_‐concentrating mechanism (CCM). Based on physiology, the freshwater plant *Ottelia alismoides* has three CCMs: C_4_ metabolism (NAD‐malic enzyme (NAD‐ME) subtype) and bicarbonate‐use during the day plus crassulacean acid metabolism (CAM) at night and lacks Kranz anatomy.
- Here, we combined a range of techniques including analysis of enzyme activity and location, transcriptomics, proteomics and ^13^C labelling in plants grown at low and high concentrations of CO_2_ to investigate how these CCMs interact and can be integrated without Kranz anatomy.
- We showed that, unlike canonical NAD‐ME subtypes, malate is the first stable compound, produced by a cytosolic malate dehydrogenase, rather than aspartate produced by aspartate aminotransferase. CAM depends on the nocturnal synthesis and transport of malic acid into the vacuole involving a vacuolar‐ATPase and a tonoplast dicarboxylate transporter that are highly expressed at night.
- These results show that C_4_ and CAM are compatible within a single cell, thanks to temporal regulation and expression of different isoforms of key enzymes and transporters. They contribute to the growing appreciation of the diversity of CCMs and how different processes can co‐occur and be coordinated. This study presents a model that could facilitate future plant engineering.

## Abstract 2

See also the Commentary on this article by Horiguchi, **249**: 2160–2162.

## Introduction

CO_2_‐concentrating mechanisms (CCMs) maximize productivity in many species. They concentrate CO_2_ around the active site of the core carboxylation enzyme of the primary fixation mechanism, Ribulose bisphosphate carboxylase‐oxygenase (Rubisco), within the Calvin–Benson–Bassham (CBB) cycle that operates during the day. These CCMs can be either biochemical or biophysical. Two biochemical CCMs have been described: C_4_ photosynthesis (Sage *et al*., 2012) that fixes carbon during the day and crassulacean acid metabolism (CAM) (Winter & Smith, 2022) that fixes carbon at night. In both, a C_4_ compound, initially produced by phosphoenolpyruvate carboxylase (PEPC), is subsequently decarboxylated around Rubisco. In C_4_ photosynthesis, carboxylation and decarboxylation occur during the day, but in CAM plants, carbon uptake and fixation by PEPC occurs at night. The C_4_ acid produced is stored in the vacuole and decarboxylated during the day nearby Rubisco, often when stomata are closed to minimize water loss for terrestrial plants. About 9% of terrestrial plant species, mainly from regions that experience water stress, or high light and high temperature, possess biochemical CCMs (Silvera *et al*., 2010; Sage *et al*., 2011). For aquatic plants, low rates of CO_2_ diffusion through substantial boundary layers and depletion of CO_2_ in productive sites are compensated by a biophysical CCM based on bicarbonate acquisition (Allen & Spence, 1981) that is found in *c*. 44% of tested freshwater species (Iversen *et al*., 2019). In addition, C_4_ photosynthesis occurs in *c*. 4% of freshwater species, including the well‐studied monocot *Hydrilla verticillata* (Holaday & Bowes, 1980; Gontero & Maberly, 2022). CAM is found in *c*. 9% of freshwater species (Maberly & Gontero, 2018) and was first shown in the lycopod *Isoetes howelli* (Keeley, 1981). It traps respiratory CO_2_ and exploits the higher nocturnal concentrations of CO_2_ as well as CO_2_ diffusing from the sediment via air channels within the roots and leaves.

There are many variations in the structures and mechanisms that increase CO_2_ around Rubisco within the three canonical CCMs. For example, in terrestrial C_4_ plants, carboxylation and decarboxylation typically occur in different cells in Kranz anatomy, but in a few species from the dicot genera *Bienertia* and *Suaeda* (formerly *Borszczowia*), these processes occur within different regions of a single cell (Voznesenskaya *et al*., 2001; Edwards *et al*., 2004). Furthermore, in C_4_ and CAM, different enzymes are involved in decarboxylation: primarily a NADP‐malic enzyme (NADP‐ME) within the chloroplasts, a NAD‐malic enzyme (NAD‐ME) within the mitochondria or a PEP carboxykinase in the cytosol (Brautigam *et al*., 2014). In NADP‐ME C_4_ species with Kranz anatomy, malate is transferred from the mesophyll cells into the bundle sheath and pyruvate is returned to the mesophyll, while in the NAD‐ME and PEP carboxykinase subtypes, aspartate is the dominant C_4_ compound transferred into the bundle sheath cells and alanine is returned (Brautigam *et al*., 2014). It has been suggested that C_4_ and CAM cannot operate in the same tissue because of anatomical, biochemical and biophysical incompatibilities (Sage, 2002), but several C_4_ species within the terrestrial dicot genera within the order Caryophyllales, *Portulaca* and *Trianthema*, also possess low‐level CAM under drought (Guralnick *et al*., 2002; Ferrari *et al*., 2020; Winter *et al*., 2021) as does the monocot from the Poaceae *Spinifex littoreus* (Ho *et al*., 2019).

*Ottelia alismoides*, a freshwater plant from the monocot family Hydrocharitaceae (as is *H. verticillata*), is the only species known to have three CCMs: constitutive C_4_ photosynthesis, bicarbonate use, and, in addition, facultative CAM under inorganic carbon limitation (Zhang *et al*., 2014). The use of bicarbonate in addition to CO_2_ has been characterized previously and involves carbonic anhydrase and solute carrier family 4 (SLC4), a well‐known bicarbonate transporter (Huang *et al*., 2020). Here, we used approaches including transcriptomics, enzyme activity measurements, subcellular proteomics and ^13^C labelling to unravel the proteins involved in the three CCMs described above and how the CCMs interact and are regulated. The analyses of the temporal variation in transcripts and metabolites and of spatial variation of proteins substantially extend previous physiological and anatomical analysis on this species. This study also enhances our understanding of CCM diversity and integration in plants.

## Materials and Methods

### Plants cultivation and treatment

Seeds of *Ottelia alismoides* (L.) Pers. were germinated in plastic pots (15 cm diameter and 12 cm height) containing sterile soil from nearby Donghu Lake (Wuhan, China). They were covered with *c*. 2‐cm tap water with an alkalinity of *c*. 2 mequiv l^−1^ (as measured by Gran titration (Zhang *et al*., 2014)) and concentrations of *c*. 1.39 mg l^−1^ total nitrogen and *c*. 0.1 mg l^−1^ total phosphorus using methods described in the GB3838‐2002 standard (China, 2002). The pots were placed in a growth room at 25°C and illuminated with 4000 K LED lamps for 14 h per day (from 08:00 h to 22:00 h) at a photon irradiance of *c*. 150 μmol photons m^−2^ s^−1^, 400 to 700 nm (LI‐COR 1500; LI‐COR, Lincoln, NE, USA). When the seedlings were *c*. 5 cm tall, three seedlings were transplanted into new pots and six pots were placed in a white plastic container (60 cm length × 40 cm width × 35 cm height) in the growth room and filled with *c*. 70‐l tap water. The water level in each container was maintained daily, and the water was renewed about every 2 wk.

The plants (24–30 per treatment) were grown at two carbon conditions as previously described (Zhang *et al*., 2014, calculated using equations in Maberly, 1996): high CO_2_ (HC) and low CO_2_ (LC). In the LC treatment, the CO_2_ was depleted by plant photosynthesis, causing the pH to fluctuate between *c*. 8.5 and 9.9. The geometric mean pH values were 8.9 at the end of the night (07:45 h) and 9.5 at the end of the day (21:40 h). The CO_2_ concentration varied between 0.13 and 9.2 μM with an average of 3.3 μM at the end of the night and 0.8 μM at the end of the day (Supporting Information Fig. S1). In the HC treatment, CO_2_ was bubbled into the water two to three times per day to bring the pH to 6.5. The geometric average pH ranged between 6.5 at night and 7.0 during the day; the alkalinity was 2.6 mequiv l^−1^, and the CO_2_ concentration fluctuated between 501 and 2205 μM with an average of 1535 μM at the end of the night and 610 μM at the end of the day (Fig. S1). The C_3_ aquatic plant *Cabomba caroliniana* A. Gray was grown under the same conditions as *O. alismoides*.

An additional experiment was designed to follow the time course of acidity and PEPC activity (see the Materials and Methods section that are described later) in more detail over 24 h. Seeds of *O. alismoides* from Wuhan were grown outside at Haikou, China, under natural light conditions between 16 May and 17 June where the average photoperiod was 13.1 h. Potted plants were grown in buckets containing *c*.160 l of water with an alkalinity of *c*. 3.2 mequiv l^−1^ at LC and HC, produced as described previously. The water temperature varied between 28.7°C and 33.4°C, measured every minute with a HOBO MX2202 pendant. Material was harvested every 3 h at: 22:00 h, 01:00 h and 04:00 h during the night and 07:00 h, 10:00 h, 13:00 h, 16:00 h and 19:00 h during the day. To distinguish between the different PEPC isoforms responsible for C_4_ and CAM, samples from plants grown at LC were collected at 04:00 h and 10:00 h.

### Acidity and enzyme activity measurement

To measure CAM capacity, leaves from HC and LC were harvested at 7:30 h (30 min before the end of the dark period, designated dark, D) and at 21:30 h (30 min before the end of the photoperiod, designated light, L). The two factors, CO_2_ concentration and sampling time, generated four treatments: HC‐D, HC‐L, LC‐D and LC‐L. Leaves were rinsed in tap water, blotted, weighed to determine fresh weight (FW) and frozen in liquid nitrogen. Frozen leaves of *c*. 0.5 g were used to determine diel changes in acidity by titration with standardized 0.01 M NaOH to an endpoint of pH 8.3 (Zhang *et al*., 2014).

The activities of Rubisco, PEPC, pyruvate phosphate dikinase (PPDK), NAD‐ME, NADP‐ME and NAD‐MDH were measured *in vitro* by following the changing absorbance of NAD(H) or NADP(H) at 340 nm with a spectrophotometer at pH 8.0, as previously described (Zhang *et al*., 2014; Jiang *et al*., 2019). The activity of aspartate aminotransferase (AspAT), alanine aminotransferase (AlaAT) and NAD‐dependent malate dehydrogenase (NAD‐MDH) was spectrophotometrically measured *in vitro*, as previously described, by measuring the decrease in absorbance at 340 nm due to the oxidation of NADH (Hatch & Mau, 1973; Jiang *et al*., 2019), on protein extracts from HC and LC leaves harvested at 10:00 h. Enzyme activities and acidity were normalized to leaf FW. For all enzymes, we used excess substrate concentrations and excess coupled enzyme systems.

### Effect of C_4_ inhibitors on net photosynthesis

Bisindolylmaleimide IV (BIM4), a competitive inhibitor of PPDK for ATP, and okanin, an allosteric inhibitor of PEPC, were used to inhibit C_4_ metabolism (Minges *et al*., 2019) in HC and LC *O. alismoides* and the C_3_ aquatic plant *C. caroliniana* as a control. Stocks of 20 mM BIM4 or 10 mM okanin were prepared in dimethylsulfoxide (DMSO). Approximately 0.2 g FW leaves of *O. alismoides* from both treatments were sealed into a perspex chamber with a magnetic stirrer containing 37 ml of 25 mM HEPES at pH 7.5. The O_2_ concentration was reduced to between 4.8 and 5.6 mg l^−1^ by bubbling with N_2_, and equimolar concentrations of NaHCO_3_ and KHCO_3_ at an overall concentration of 5 mM were added. The O_2_ exchange rate was measured over 5–10 min at 200 μmol photons m^−2^ s^−1^ irradiance and 25°C using an O_2_ electrode (YSI Pro ODO Yellow Spring Instruments, Yellow Springs, OH, USA). Rates were measured at different concentrations of BIM4 or okanin (2–70 μM) or DMSO alone (0.2%, corresponding to the DMSO concentration at the highest inhibitor concentration). The same treatments were made with the C_3_ aquatic plant *C. caroliniana* as a control. Each concentration was replicated 3–6 times. The IC_50_ concentration (the concentration required to inhibit photosynthesis by 50%) was calculated by fitting the absolute rates against concentration with a three‐parameter logistic model.

### ^13^C labelling kinetics and metabolite analysis

Leaves of *O. alismoides* (50 mg FW) collected from the growth chamber, described previously, during the day at 14:00 h. They were placed in 100 ml of 1 mM NaH^13^CO_3_ at pH 7.0 for HC and pH 8.5 for LC for 0, 5, 30, 60, 300, 600, 1200, 2400 and 3600 s in the growth chamber. After labelling and quenching in liquid nitrogen, three zirconia beads (3 mm diameter, prechilled at −80°C) and 600 μl methanol (prechilled at −20°C) were added to each sample. After homogenization at 60 Hz for 1 min, 600 μl of chloroform (prechilled at −20°C) was added to each sample and shaken at 1400 rpm for 1 min. Subsequently, 240 μl double distilled H_2_O (room temperature) was added, and samples were vortexed for 1 min. The samples were kept on dry ice for 10 min and then centrifuged at 4°C, at 13 400 ***g***, for 10 min. The supernatant (250 μl) was collected, transferred to a new 1.5‐ml Eppendorf tube, and dried using a vacuum centrifuge. Subsequently, samples were derivatized with 100 μl of solution comprising acetonitrile (20 μl), boric acid (70 μl, 50 mM H_3_BO_3_ buffer, pH 4.0), and 8‐(diazomethyl) quinoline (10 μl 8‐DMQ (10 mg ml^−1^)). 8‐DMQ was synthesized according to a previous study (Jiang *et al*., 2018). After centrifugation for 10 min at 4°C and at 13 400 ***g***, a 30 μl aliquot of supernatant was analysed by Liquid Chromatography coupled to tandem mass spectrometry (MS) analysis. This analysis was performed on a Vanquish UHPLC system (Thermo Scientific, Waltham, MA, USA) coupled to a ThermoFisher Q‐Exactive HFx high‐resolution mass spectrometer with a HESI‐II ion source (Thermo Scientific). Liquid Chromatography separation was accomplished on a Waters Acquity UPLC CSH C18 column (100 × 2.1 mm, 1.7 μm) at a flow rate of 0.4 ml min^−1^. Mobile phases A and B were an ammonium acetate aqueous solution (10 mM) and acetonitrile, respectively. The gradient was as follows: 0 to 1 min from 10% B to 12% B, 1 to 3 min, 12% B to 95% B and maintained at 95% B for 1 min, then 4–4.01 min from 95% B back to 10% B, and 4.01 to 6.5 min, re‐equilibrated at 10% B. The ion source conditions were as follows: spray voltage, 3.6 kV; sheath gas flow rate, 30 arbitrary units; auxiliary gas flow rate, 10 arbitrary units; capillary temperature, 320°C; S‐lens RF level, 50; and probe heater temperature, 400°C. A data‐dependent acquisition mode was employed for MS/MS analysis. The MS parameters were as follows: MS1 mass range, *m/z* 66.7–1000; MS1 resolution, 60 000 fwhm (*m/z* 100); number of data‐dependent scans per cycle, 4; MS/MS resolution, 15 000 fwhm (*m/z* 50); and normalized collision energy, 10%, 20%, 30%. Standard curves with malate, aspartate, alanine, 3‐phosphoglycerate (PGA) and pyruvate (0, 50, 100, 200, 500 and 1000 ng ml^−1^) were used to calculate their concentration in the samples; *R* ^2^ values of the standard curves for the five metabolites varied between 0.91 and 1.00, with an average of 0.97.

The ^13^C enrichment was calculated as previously described (Szecowka *et al*., 2013). The amount of ^13^C in each measured organic acid at each sampling time was calculated using the ^13^C percentage multiplied by its content. Because the initial (0 s) amount of ^13^C in each organic acid was different, the ^13^C amount was adjusted by the ^13^C amount at each sampling time minus the initial amount of ^13^C.

### RNA sequencing from HC and LC plants

Approximately 0.5 g FW leaves was harvested and frozen as described previously. Three leaves from independent plants were used for each of the four treatments: HC‐D, HC‐L, LC‐D and LC‐L. Total RNA was extracted using a commercial kit, RNAiso (Takara Biotechnology, Dalian, China), and then dissolved in RNase‐free water with DNase I (Promega, Beijing, China). RNA quality was checked with an Agilent 2100 Bioanalyzer (Agilent Technologies, Palo Alto, CA, USA). Only the total RNA samples with RNA integrity numbers ≥ 8 were used to construct the cDNA libraries.

Long‐read RNA sequencing (PacBio, Menlo Park, CA, USA) was conducted as follows. Total RNA (2 μg) from two different treatments (HC and LC) at two different times of day (D and L: HC‐D, HC‐L, LC‐D and LC‐L) was mixed together. The mixture was reverse‐transcribed into cDNA using a SMARTer PCR cDNA Synthesis Kit (Takara Biotechnology) following the manufacturer's protocol. After size selection using the BluePippin™ Size Selection System (Sage Science, Beverly, MA, USA), the size‐selected cDNA (1–2 μg) was used to construct a single‐molecule real‐time (SMRT) cell library using the Pacific Biosciences DNA Template Prep Kit 2.0. SMRT sequencing was performed on the Pacific Biosciences Sequel platform using the manufacturer's protocol.

Short‐read RNA sequencing (Illumina HiSeq) analyses were conducted following our previous work (Huang *et al*., 2020). A total amount of 1.5 μg RNA per sample was used. Sequencing libraries were generated using NEBNext® UltraTM RNA Library Prep Kit for Illumina® (NEB, Ipswich, MA, USA) following the manufacturer's recommendations, and index codes were added to attribute sequences to each sample. Briefly, mRNA was purified from total RNA using poly‐T oligo‐attached magnetic beads. Fragmentation was carried out using divalent cations under elevated temperature in NEBNext First‐Strand Synthesis Reaction Buffer (5×). First‐strand cDNA was synthesized using random hexamer primer and Moloney murine leukaemia virus Reverse Transcriptase (RNaseH‐). Second‐strand cDNA synthesis was subsequently performed using DNA Polymerase I and RNase H. Remaining overhangs were converted into blunt ends via exonuclease/polymerase activities. After adenylation of 3′ ends of DNA fragments, NEBNext Adaptor with hairpin loop structure was ligated to prepare for hybridization. In order to select cDNA fragments higher than 100 bp in length, the library fragments were purified with the AMPure XP system (Beckman Coulter, Beverly, USA). Then, 3 μl USER Enzyme (NEB, USA) was used with size‐selected, adaptor‐ligated cDNA at 37°C for 15 min followed by 5 min at 95°C. PCR was performed with Phusion High‐Fidelity DNA polymerase, Universal PCR primers, and Index (X) Primer. Finally, products were purified (AMPure XP system), and library quality was assessed on the Agilent Bioanalyzer 2100system. The clustering of the index‐coded samples was performed on a cBot Cluster Generation System using HiSeq 4000 PE Cluster Kit (Illumina) according to the manufacturer's instructions. After cluster generation, the library preparations were sequenced on an Illumina Hiseq 4000 platform and 150‐bp paired‐end reads were generated.

The long‐read RNA sequences subreads were filtered using the standard protocols in the SMRT analysis software suite (http://www.pacifcbiosciences.com), and reads of insert (ROIs) were generated. Full‐length nonchimeric (FLNC) reads and nonfull‐length (NFL) cDNA reads were recognized through the identification of poly (A) signal and 5′ and 3′ adaptors. The FLNC reads were clustered and polished by the Quiver program with the assistance of NFL reads, producing high‐quality isoforms (HQ) and low‐quality isoforms (LQ). The raw Illumina reads were filtered to remove ambiguous reads with ‘N’ bases, adaptor sequences and low‐quality reads. Filtered Illumina data were then employed to polish the LQ reads using the proovread 213.841 software. The redundant isoforms were then removed to generate a high‐quality transcript dataset for *O. alismoides*, using the program CD‐HIT. The clean reads from short‐read RNA sequences were mapped to the reference sequences from the long‐read RNA sequences (34 708 FLNC) using the BWA software. Transcript levels are not only reported as total counts (Reads per Kilobase per Million (RPKM)) but also shown as change‐fold relative to a control housekeeping gene (E3 ubiquitin protein ligase; Fig. S2), as described by Guo *et al*. (2025).

Each protein isoform contains many (several) transcripts that show the same expression pattern, and these transcripts were combined to construct a general linear model (glm). The treatment (four conditions) was set as a fixed factor, and transcripts were set as a random factor in R (4.5.0 version for windows). The model was analysed using the ‘ANOVA’ function in the car package to determine whether the treatments significantly affected protein isoform expression. If the ANOVA was significant (*P* < 0.05), a least significant difference method was used to perform multiple comparisons between each condition, with the *P* value adjusted using the ‘Bonferroni’ correction.

The transcripts were annotated based on Gene Ontology (GO) (https://www.geneontology.org/), Kyoto Encyclopedia of Genes and Genomes (KEGG) (https://www.kegg.jp/), NR (https://www.ncbi.nlm.nih.gov/refseq/about/nonredundantproteins/), Swiss‐prot (https://www.uniprot.org/) and pfam (https://pfam.xfam.org/). These data also provided amino acid sequence information, since although the genome of *O. alismoides* has been recently sequenced, it is not fully assembled (Wang *et al*., 2024). The amino acid sequences derived from coding DNA sequences are deposited under the database China National GeneBank (CNGB; https://db.cngb.org/): CNP0007412, CNP0007421 and are available publicly. The amino acid sequences of proteins relevant to this study are shown in Table S1.

### Location of candidate CCM proteins

Four methods were used to localize the CCM marker proteins. (1) Activities of Rubisco, PEPC, PPDK, NAD‐ME, NADP‐ME and NAD‐MDH were measured separately in leaves, purified chloroplasts and mitochondria as described previously. The organelle purification protocol is described later, and their purity was checked using microscopy (Fig. S3). The activities were normalized by total protein content. (2) Western blots of Rubisco, PEPC and NAD‐ME were performed with proteins from leaves, purified chloroplasts and mitochondria as described previously (Casati *et al*., 2000). Western blots using antibodies of these enzymes also allowed cross‐contamination between chloroplasts and mitochondria to be checked (Fig. S4a–c). The protein content loaded on the SDS‐gel before nitrocellulose transfer was 2.5 μg for Rubisco and 30 μg for PEPC and NAD‐ME. Polyclonal antibodies against PEPC (1:1000; Agrisera, Vännäs, Sweden), NAD‐ME (1:1000; Agrisera) and Rubisco large subunit (rbcL, 1:1000) were used. The polyclonal antibody of rbcL was prepared by injecting the rbcL (overexpressed in *Escherichia coli* using a *rbcL*‐containing pET‐32a plasmid) and Freund's Adjuvant Complete into a rabbit. Horseradish peroxidase‐conjugated goat anti‐rabbit IgG (1:1000; Servicebio, Wuhan, Hubei, China) was the secondary antibody. (3) MS was used to determine the relative intensity of different isoforms of candidate CCM proteins in leaves, from plants collected at 10:00 h, purified chloroplasts and mitochondria. MS was performed by SpecAlly Life Technology Co., Ltd (Wuhan, China). In brief, *c*. 100 μg of total leaf, chloroplasts and mitochondria proteins were incubated in 100 mM Tris–HCl buffer with 8 M urea and 10 mM dithiothreitol at 37°C for 1 h. After alkylation with 50 mM iodoacetamide, the urea was diluted to 2 M with 100 mM Tris–HCl, pH 8.5 and the proteins were digested overnight with 2 μg trypsin. The resulting peptides were desalted using a Sep‐Pak C18 column and analysed with an EASY 1200 nanoflow Liquid Chromatography (EASY‐nLC™ 1200) coupled to a Q Exactive HF‐X mass spectrometer. The analysis was performed with the MaxQuant (v.1.6.6) software using a database of Fasta sequences of *O. alismoides* that included all the isoform sequences of the candidate CCM proteins derived from our transcriptomic data. The MaxQuant output was expressed as iBAQ (Intensity = Based Absolute Quantification) that gives the absolute abundance of each protein within each sample. (4) Software that analyses the signal peptide sequence (targetP 2.0; https://services.healthtech.dtu.dk/services/TargetP‐2.0/) was also used to predict the location of the proteins.

### PEPC phosphorylation analysis using mass spectrometry

Leaves collected from plants grown at LC at 04:00 h, 07:30 h, 13:00 h and 21:30 h were used to follow the phosphorylation state of PEPC. Approximately 100 μg of total leaf proteins was reduced and alkylated with 10 mM tris(2‐carboxyethyl)phosphine and 40 mM 2‐chloroacetamide at 37°C for 1 h. Digestion occurred overnight at 37°C after the addition of 2 μg trypsin and was stopped by adding trifluoroacetic acid that reduced the pH to 6.0. After centrifugation (12 000 ***g***, 15 min), the peptides in the supernatant were purified using a self‐made styrene divinyl benzene‐reverse phase sulphonate desalting column. The peptides were vacuum‐dried and stored at −20°C. All peptides were analysed with a hybrid trapped ion mobility spectrometer (TIMS) quadrupole time‐of‐flight mass spectrometer (timsToF Pro; Bruker Daltonics, Coventry, UK). An UltiMate 3000 RSLCnano system (Thermo Fisher) was coupled to timsTOF Pro with a CaptiveSpray nano ion source (Bruker Daltonics). Peptide samples were injected into a C18 Trap column (75 μm × 2 cm, 3 μm particle size, 100 Å pore size; Thermo Fisher) and separated in a reversed‐phase C18 analytical column (75 μm × 15 cm, 1.7 μm particle size, 100 Å pore size; IonOpticks). Mobile phase A (0.1% formic acid in water) and mobile phase B (0.1% formic acid in acetonitrile) were used to establish the separation gradient at a flow rate of 300 nl min^−1^. The MS data were acquired in Parrallel Accumulation‐Serial Fragmentation (PASEF) mode. The capillary voltage was set to 1500 V. The MS and MS/MS spectra were acquired from 100 to 1700 *m/z*. The ion mobility was scanned from 0.75 to 1.4 Vs cm^−2^. The accumulation time and ramp time were set to 166 ms. The acquisition cycle of 1.88 s comprised one full MS scan and 10 PASEF MS/MS scans. Singly charged precursors were filtered out by ion mobility, and only precursor signals over an intensity threshold of 1000 were picked for fragmentation. Target intensity was set to 20 000. Precursors were dynamically excluded for 0.4 min. The quadrupole isolation width was set to 2.0 Da at *m*/*z* 700, 3.0 Da at *m*/*z* 800. The collision energy was ramped linearly as a function of the mobility from 59 eV at 1/K0 = 1.6 Vs cm^−2^ to 20 eV at 1/K0 = 0.6 Vs cm^−2^.

Raw MS data were analysed with FragPipe (FragPipe‐v.22.0) using the MSFragger database search algorithm. Spectra files were searched against the target protein sequence database of FASTA sequences of *O. alismoides* that included all the isoform sequences of the candidate CCM proteins derived from our transcriptomic data with the precursor and fragment mass tolerances set to 15 and 20 ppm. Oxidation of methionine, N‐terminal acetylation and Phospho (STY, 79.9663 Da) were set as variable modifications. Post‐Translational Modification (PTM) site localization with PTMProphet was enabled. A false discovery rate of 0.01 was set both at the peptide and the protein level.

### Chloroplast isolation

Leaves of *O. alismoides* were harvested from the LC treatment at 18:00 h and kept for 14 h in the dark in the LC water to exhaust any starch to minimize chloroplast rupture on isolation (Halliwell, 1981). The next morning, the leaves were washed, blotted and separated into two halves by removing the main vein. Approximately 10 g FW was used to isolate the intact chloroplasts after 2.5 to 4 h at *c*. 50 μmol m^−2^ s^−1^ according to a previous study (Kubis *et al*., 2008). Subsequently, the chloroplasts were purified in 30% Percoll (in a buffer with 0.4 M sucrose, 0.05 M Tris, 0.01 M NaCl, pH 7.6) at 11 000 ***g***, 4°C for 10 min. The purified chloroplasts were checked under a light microscope (Olympus BX53, Melville, NY, USA), then broken in 200 μl minute nondenatured protein solubilization reagent (Invent Biotechnologies, Eden Prairie, MN, USA) and centrifuged at 12 000 ***g***, and 4°C for 5 min.

### Mitochondrion isolation

Approximately 40 g leaves from the LC treatment at the start of the photoperiod was harvested, washed, blotted, weighed and cut into small segments. The intact mitochondria were isolated and purified according to a previous study (Kerbler & Taylor, 2017). The purified mitochondria were checked using the light microscope after staining with 10 μl 1% (w/v stock solution) Janus green B for 1 min. They were then broken in 100 μl minute nondenatured protein solubilization reagent, centrifuged at 12 000 ***g*** and 4°C for 5 min, and the mitochondria and the supernatant were collected.

### Immunogold labelling of Rubisco

Fresh leaves from LC were cut into 1 × 3 mm segments and fixed in 3% paraformaldehyde and 1% glutaraldehyde (0.1 M phosphate buffer, pH 7.4) at 4°C for 2–4 h. The segments were rinsed with 0.1 M phosphate buffer (pH 7.4) three times and once with 0.1 M phosphate buffer (pH 7.4) with 0.1 M glycine, 30 min for each. The leaf segments were dehydrated under a series of ethanol solutions (30, 50, 70, 90%) for 1 h, followed by dehydration in 100% ethanol three times for 1 h at −20°C. The dehydrated leaf segments were penetrated using 30, 70 and 100% Lowicryl K4M at −20°C for 2 h. They were then embedded in Lowicryl K4M at −20°C under UV light for 72 h, and at room temperature under UV light for 48 h. After cutting into 90‐nm transverse sections, the sections were placed in a blocking solution (1% BSA, 0.02% PEG20000, 100 mM NaCl and 0.1% NaN_3_ prepared in 50 mM phosphate buffer at pH 7.0) for 30 min at room temperature and then incubated with polyclonal antibody of rbcL (1: 1000) for 2 h at room temperature. After rinsing using 0.01 M phosphate buffer (pH 7.4) six times for 2 min each, the section was incubated with gold‐conjugated goat anti‐rabbit IgG (G7402, 1:200; Sigma) for 1 h. The residual gold‐labelled antibody was rinsed using 0.01 M phosphate buffer (pH 7.4) six times for 2 min each and pure water four times for 2 min each. The labelled sections were air‐dried, dyed using uranyl acetate and lead citrate, and then observed using a transmission electron microscope (Hitachi High‐Tech, Tokyo, Japan).

### Distance between chloroplasts and mitochondria

Leaves from HC and LC plants were collected 2.5 h into the photoperiod (10:30 h) and fixed in 2.5% glutaraldehyde (0.1 M phosphate buffer, pH 7.4). Ultrathin transverse sections were prepared as previously described (Han *et al*., 2020). Images were obtained with a transmission electron microscope (Hitachi High‐Tech, Tokyo, Japan), and the distance between chloroplasts and mitochondria was measured using the ImageJ software (NIH, Bethesda, MD, USA).

## Results

### Response of gene expression to light and inorganic carbon

RNA sequencing of *O. alismoides* grown in HC and LC and collected in the dark and light (D and L) generated 34 708 transcripts, of which 32 959 were annotated. We identified 13 610 differentially expressed genes (DEGs), and a principal component analysis (PCA) showed that transcript expression in the four conditions differed (Fig. S5a). The differences between light and dark explained 79% of the variance in the PCA and included 10 962 DEGs (Fig. S5b). The difference between LC and HC explained 7% of the variance in the PCA and included 2648 DEGs (Fig. S5c). In the dark, 757 DEGs were more expressed at LC than in HC. In the light, 627 DEGs were more expressed at LC than in HC. DEGs were classified into different categories using GO. Although our focus here is to analyse genes associated with C_4_ and CAM, other categories of GO were affected, including hormone signalling, amino acid and nitrogen metabolism, starch and sucrose synthesis, pigment biosynthesis, and lipid oxidation and transport, and are presented in Fig. S6 for general information.

### Carbon assimilation

**Fig. 1:**
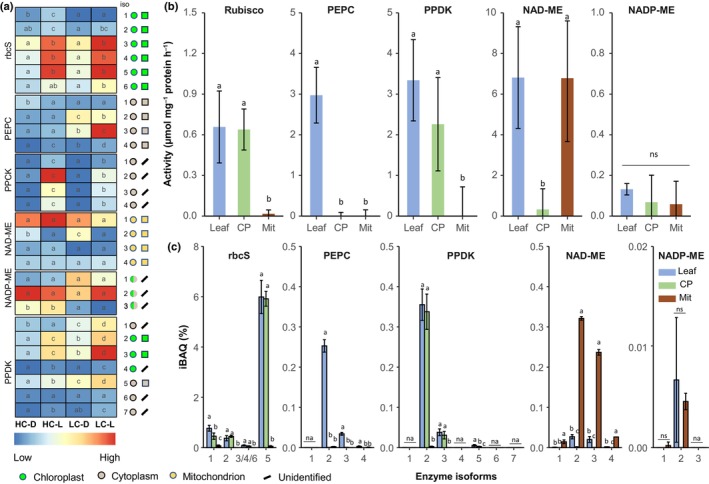
Enzymes involved in carbon assimilation in *Ottelia alismoides*. (a) Heatmap of protein transcript levels (RPKM) of plants grown at high carbon (HC) or low carbon (LC) and collected in the dark (D) or light (L). (b) Activity of key enzymes in leaf extracts, purified chloroplasts (CP) or purified mitochondria (Mit) from LC plants collected during the day. (c) Relative abundance of protein isoforms (Intensity = Based Absolute Quantification (iBAQ)) for key enzymes from leaf extracts, CP or Mit for LC plants collected during the day. The location of each isoform is shown based on prediction (coloured circles) or iBAQ measurements (coloured squares). Enzymes are: Ribulose bisphosphate carboxylase‐oxygenase (Rubisco), PEP carboxylase (PEPC), PEPC kinase (PPCK), pyruvate phosphate dikinase (PPDK), NAD(P) malic enzyme (NAD(P) ME), small subunit of Rubisco (rbcS). *n* = 3 with SD shown by error bars. Statistically significant differences (*P* < 0.05) among samples are indicated by different lower case letters, calculated with a Generalized Linear Model and an ANOVA for (a) and a one‐way ANOVA for (b, c). ns, no significant difference.

In *O. alismoides*, as expected, Rubisco, the primary carboxylase, was chloroplastic based on compartmentalized activity, subcellular proteome and western blots (Figs 1b,c, S4a). Immunogold labelling showed that Rubisco was present in the upper and lower epidermis and in the mesophyll cells (Fig. S4d). Analysis of transcript expression showed that Isoforms 3, 4 and 5 of the small subunit of Rubisco were more expressed in the light than the dark for HC and LC plants (Fig. 1a). The subcellular proteomic data showed that Isoform 5 was the most abundant isoform in plants collected during the day (Fig. 1c). As expected, isoforms of other key enzymes from the CBB cycle, phosphoribulokinase (PRK; Fig. S7) and the two subunits of the chloroplastic glyceraldehyde‐3‐phosphate dehydrogenase (GAPDH A_2_B_2_; Fig. S7) were also highly expressed in the light in both treatments.

*O. alismoides* has constitutive C_4_ and facultative CAM (Zhang *et al*., 2014; Shao *et al*., 2017; Han *et al*., 2020; Huang *et al*., 2020). Studies of the effect of inhibitors on oxygen evolution provided additional evidence for C_4_ metabolism in *O. alismoides*. The chalconoid okanin, an allosteric inhibitor of PEPC (Minges *et al*., 2019), inhibited oxygen evolution with an IC_50_ of 14 ± 2 μM at HC and 21 ± 2 μM at LC (not significantly different, *P* = 0.10) while in *C. caroliniana*, it was > 70 μM in both treatments (Fig. S8a). Similarly, BIM4, a competitive inhibitor of PPDK for ATP (Minges *et al*., 2019), had an IC_50_ of 50 ± 8 (SD) μM at HC and 56 ± 11 at LC (not significantly different, *P* = 0.62) while in *C. caroliniana*, it was > 70 μM in both treatments (Fig. S8a). At the highest concentration used, inhibition of *C. carolinian*a across the four treatments ranged between 20 and 44%, but in *O. alismoides*, it was higher and ranged between 68 and 87%. Dimethyl sulfoxide used to dissolve the inhibitors did not have a significant effect (Fig. S8b).

**Fig. 2:**
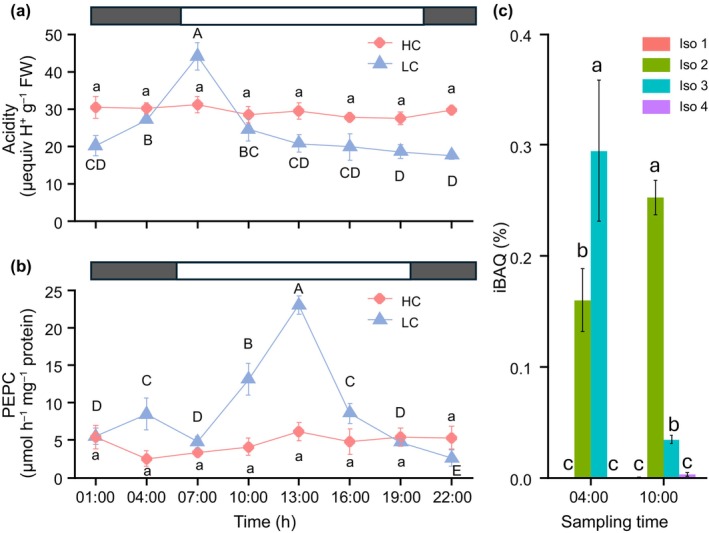
Temporal changes in acidity, phosphoenolpyruvate carboxylase (PEPC) activity, and PEPC isoform expression in *Ottelia alismoides* grown outside under natural irradiance at *c*. 30°C. (a) Changes in acidity in plants grown at high carbon (HC) or low carbon (LC). (b) Changes in PEPC activity in plants grown at HC or LC. (c) Relative abundance of PEPC isoforms (Intensity = Based Absolute Quantification) for leaves collected in the night (04:00 h) and day (10:00 h) and grown at LC. The photoperiod is shown by the white bars and extended from 05:58 to 19:14 h. *n* = 3 with SD shown by error bars. Statistically significant differences (*P* < 0.05) among samples are indicated by different letters calculated with a generalized linear model and an ANOVA for (a, d) and a one‐way ANOVA for (b, c, e–g).

The plants used here expressed CAM with a statistically significant diel variation of 10.7 μequiv H^+^ g^−1^ FW, only when grown at LC (Fig. S9). In the experiment with greater temporal resolution, there was no diel variation in acidity over 24 h in HC plants grown outside. By contrast, LC plants had a peak of acidity at 07:00 h, 1 h into the photoperiod (Fig. 2a). PEPC activity over 24 h for the plants grown outside showed two peaks, one during the night that is consistent with CAM activity consistent with the acidity increase, and one during the day that is consistent with C_4_ activity (Fig. 2b).

All four isoforms of PEPC were cytosolic (Figs 1a, S4b), and activity was also found in the cytosol (Fig. 1b). PEPC transcripts of Isoforms 2 and 3 were more expressed in light and dark in LC plants than in HC plants, and these isoforms are likely to be involved in C_4_ and CAM.

Based on the transcript levels, Isoform 3 was more abundant than the other isoforms, and its level was higher at 21:30 h (light) than at 07:30 h (dark) in LC plants (Fig. 1a). Isoform 2 was, by contrast, more abundant at the transcript level in the dark than in the light in LC plants. Isoforms 1 and 4 were less abundant than Isoforms 2 and 3 in LC plants. At the protein level, using iBAQ, for samples from LC plants, PEPC isoform 3 was the most expressed isoform during the night (04:00 h), and PEPC isoform 2 was the most expressed during the day (10:00 h) (Figs 1, 2c).

As phosphorylation plays a role in the regulation of PEPC, we analysed this modification by MS and confirmed that PEPC was phosphorylated during the day at 13:00 h and 21:30 h (Table S2). During the night, PEPC was phosphorylated at 04:00 h, at a time when acidity levels increased, but not at 07:30 h just before the start of the photoperiod (Fig. 2a; Table S2). It was impossible to attribute the individual phosphorylation state of Isoforms 2, 3 and 4 because Ser15 that is phosphorylated is present and identical in all these isoforms. By contrast, PEPC isoform 1, a bacterial type (Fig. S10), was not phosphorylated (Table S2) and had a constant low expression (Fig. 1a).

Isoforms 2 and 3 of PPDK were highly expressed and chloroplastic (Fig. 1a). The dual location of PPDK was confirmed by its activity in the cytosol and chloroplast (Fig. 1b). Previously, we found that the activity of NAD‐ME was much higher than that of NADP‐ME (Zhang *et al*., 2014), and here, we found a *c*. 40‐fold higher activity in NAD‐ME than in NADP‐ME (Fig. 1b). As expected, the four isoforms of NAD‐ME were mitochondrial (Figs 1, S4c), and quantitative proteomics showed that Isoforms 2 and 3 were the most abundant (Fig. 1c). Protein levels of NAD‐ME were *c*. 10‐fold higher than NADP‐ME (Fig. 1c). PEP carboxykinase transcripts were not found, and based on all lines of evidence, NAD‐ME appears to be the critical decarboxylase.

**Fig. 3:**
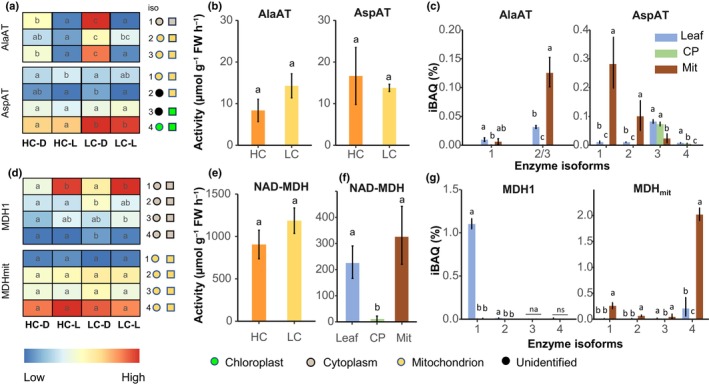
Enzymes involved in the production of malate in *Ottelia alismoides*. (a, d) Heatmap of protein transcript levels (Reads per Kilobase per Million) of plants grown at high carbon (HC) or low carbon (LC) and collected in the dark (D) or light (L). (b, e) Activity of transaminases from plants grown at HC or LC and collected during the day. (f) Activity of NAD‐dependent malate dehydrogenase (NAD‐MDH) in leaf extracts, purified chloroplasts (CP), or purified mitochondria (Mit). (c, g) Relative abundance of protein isoforms (Intensity = Based Absolute Quantification (iBAQ)) for key enzymes from leaf extracts, CP or Mit for material grown at LC and collected during the day, *n* = 3. The location of each isoform is shown based on prediction (coloured circles) or iBAQ measurements (coloured squares). Enzymes are as follows: aspartate aminotransferase (AspAT), alanine aminotransferase (AlaAT), NAD‐dependent malate dehydrogenase (NAD‐MDH). *n* = 3 with SD shown by error bars. Statistically significant differences (*P* < 0.05) among samples are indicated by different letters calculated from a one‐way ANOVA.

The product of the reaction of PEPC, oxaloacetate (OAA), can be catalysed either by AspAT into aspartate or by NAD‐MDH into malate. The C_4_ NAD‐ME subtype typically involves AspAT and AlaAT, including in single‐cell C_4_ terrestrial plants (Offermann *et al*., 2011). In *O. alismoides*, four isoforms of AspAT were expressed (Fig. 3a): two were mitochondrial and two were chloroplastic. Three isoforms of AlaAT were expressed (Fig. 3a): two were mitochondrial and one was cytosolic. At HC and LC, the activities of AspAT and AlaAT were not significantly different and were *c*. 15 and 11 μmol h^−1^ g^−1^ FW, respectively (Fig. 3b). The activity of NAD‐MDH was not significantly different at HC and LC but was 50‐ to 100‐fold higher than AspAT (Fig. 3b,e). Four isoforms of NAD‐MDH were cytosolic (MDH1), and four were mitochondrial (MDH_mit_) (Fig. 3d). Quantitative proteomic analysis showed that the protein levels of MDH_mit_ and MDH1 were two‐ to 10‐fold higher than AspAT (Fig. 3c,g). All the isoforms of MDH_mit_ were constitutively expressed regardless of the CO_2_ and light conditions (Fig. 3d). By contrast, Isoform 1 of MDH1 was highly expressed under HC‐L and LC‐L, and Isoform 2 was most highly expressed under LC‐D (Fig. 3d), suggesting that Isoform 1 is involved in C_4_ and Isoform 2 is involved in CAM.

### Metabolite pool sizes and ^13^C isotope labelling

**Fig. 4:**
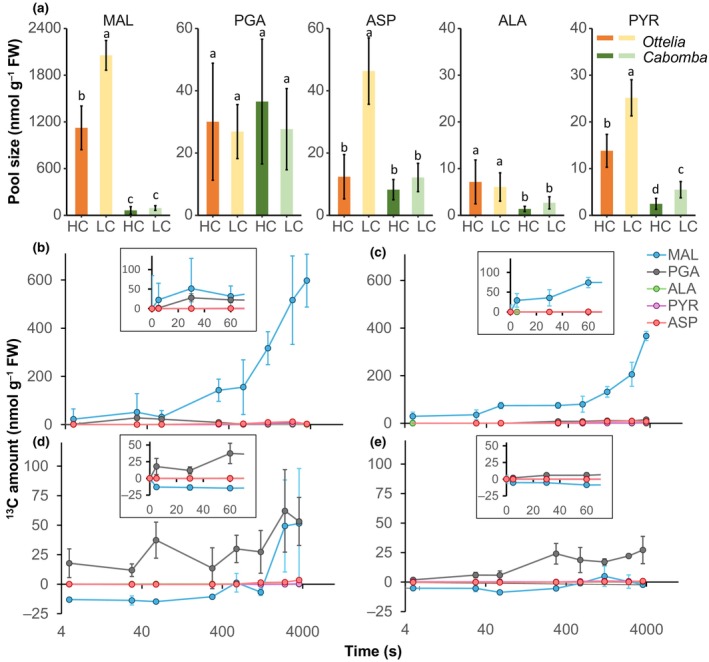
^13^C labelling kinetics for material collected during the day. (a) Content of metabolites, malate (MAL), phosphoglycerate (PGA), aspartate (ASP), alanine (ALA) and pyruvate (PYR) in *Ottelia alismoides* and *Cabomba caroliniana*. (b–e) Kinetics of ^13^C amount for different metabolites in *O. alismoides* grown at high carbon (HC) (b) or low carbon (LC) (c) and *C. caroliniana* grown at HC (d) or LC (e). The inserts show the ^13^C amount for the first 60 s. *n* = 3 with SD shown by error bars. Statistically significant differences (*P* < 0.05) among samples are indicated by different lower case letters calculated from a one‐way ANOVA.

The activity and location data suggest that MDH1 rather than AspAT was responsible for producing malate in *O. alismoides*. ^13^C isotope labelling and MS were used to analyse metabolite pool size and flux and test this hypothesis further. Five compounds were measured: PGA, malate, aspartate, alanine and pyruvate for samples collected at 14:00 h. The pool size of malate was the largest of these five compounds (Fig. 4a). It was *c*. 20‐fold greater in both HC and LC in *O. alismoides* than in the C_3_ plant *C. caroliniana* (Fig. 4a). In *O. alismoides*, the pool size of malate in LC was 1.8‐fold higher than in HC. The pool size of PGA, the first product of C_3_ photosynthesis, was not significantly different across species and treatments. The aspartate and pyruvate pool sizes were also higher in *O. alismoides* grown at LC vs HC and also higher than in *C. caroliniana*. Nonetheless, in *O. alismoides*, the aspartate and pyruvate pools were 45‐fold and 82‐fold smaller than malate. The pool size of alanine was the same across HC and LC for both species and was the smallest of the five compounds (Fig. 4a).

^13^C enrichment showed that malate was labelled within the first 5 s in HC and LC *O. alismoides*, but not in *C. caroliniana*, whereas PGA was labelled in both species (Fig. S11). The ^13^C amount, after correcting for the initial amount at 0 s in each compound, was calculated from the pool size (Fig. 4a) and ^13^C percent in each compound (Fig. S11). At HC, the proportion of ^13^C label in malate vs PGA was 93%, 65% and 59% at 5, 30 and 60 s, respectively (Fig. 4b). In LC *O. alismoides*, *c*. 100% of the ^13^C amount occurred in malate during the first 60 s (Fig. 4c). However, in *C. caroliniana* under both treatments, 100% of the ^13^C label was found in PGA in the first 60 s (Fig. 4d,e). The proportion of ^13^C amount in aspartate vs malate was 1–2% in the first 5–300 s in *O. alismoides* under both treatments. These results further support the contention that the C4 pathway in *O. alismoides* involves MDH rather than AspAT.

### Transporters involved in C_3_ , C_4_ and CAM

**Fig. 5:**
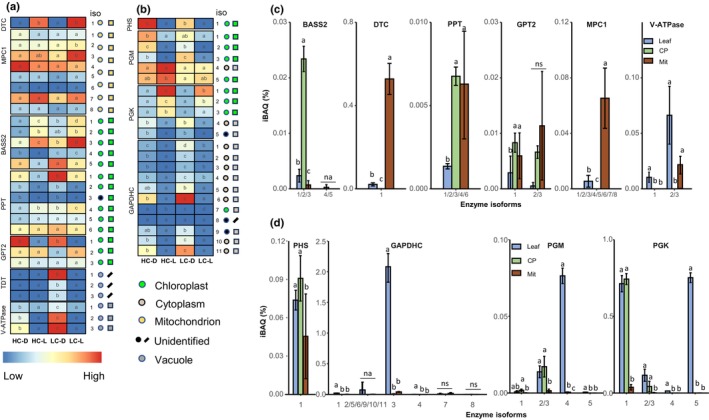
Enzymes involved in metabolite transport or glycolysis in *Ottelia alismoides*. (a, b) Heatmap of protein transcript levels (Reads per Kilobase per Million (RPKM)) and significant differences shown by letters (*P* < 0.05) of plants grown at high carbon (HC) or low carbon (LC) and collected in the dark (D) or light (L). (c, d) Relative abundance of protein isoforms (Intensity = Based Absolute Quantification (iBAQ)) for key transporters and enzymes from leaf extracts, purified chloroplasts (CP) or purified mitochondria (Mit) for material grown at LC and collected during the day. The location of each isoform is shown based on prediction (coloured circles) or iBAQ measurements (coloured squares). Enzyme and transporter names are as follows: dicarboxylate/tricarboxylate transporter (DTC), bile acid sodium symporter (BASS2), phosphoenolpyruvate transporter (PPT), glucose‐6‐phosphate translocator (GPT2), mitochondrial pyruvate carrier (MPC1), tonoplast dicarboxylate transporter (TDT), vacuolar‐ATPase (V‐ATPase), starch phosphorylase (PHS), phosphoglucomutase (PGM), phosphoglycerate kinase (PGK) glyceraldehyde‐3‐phosphate dehydrogenase (GAPDHC). *n* = 3 with SD shown by error bars. Statistically significant differences (*P* < 0.05) among samples are indicated by different lowercase letters calculated with a generalized linear model and an ANOVA for (a, b) and a one‐way ANOVA for (c, d).

Membrane transporters are required to exchange metabolites between compartments (Fig. 5). The single isoform of the dicarboxylate/tricarboxylate transporter (DTC) that can transport malate (Picault *et al*., 2004) was more expressed in the light than in the dark and was mitochondrial (Fig. 5a,c). Of the eight isoforms of the mitochondrial pyruvate carriers 1 (MPC1), two were highly expressed under LC‐L and were mitochondrial (Fig. 5a,c). Five isoforms of bile acid sodium symporter (BASS2), allowing pyruvate transport across the chloroplast membranes, were indeed chloroplastic; among them, two were most expressed in the light (Fig. 5a,c). Among the six isoforms of phosphoenolpyruvate transporter (PPT), a translocator for PEP and inorganic phosphate, Isoform 1 was the most highly expressed in LC‐D, while Isoform 4 was the most highly expressed in LC‐L; both were chloroplastic (Fig. 5a,c). The three isoforms of the tonoplast dicarboxylate transporter (TDT) that transport malate into the vacuole were only expressed in LC‐D (Fig. 5a), and Isoforms 2 and 3 of the vacuolar membrane proton pump V‐ATPase were most highly expressed in LC‐D (Fig. 5a,c), consistent with both being involved in CAM. Isoform 1 out of three isoforms of the translocator for glucose‐6‐phosphate translocator (GPT2) was most highly expressed in the dark in both LC and HC and was chloroplastic (Fig. 5a,c).

### Glycolysis

During the night, the degradation of starch and glycolysis is necessary to support CAM as well as background respiration. The single isoform of starch phosphorylase was more highly expressed in the dark than in the light (Fig. 5b) and was chloroplastic (Fig. 5d). Isoforms 1, 2, 3 and 5 of phosphoglucomutase (PGM) were chloroplastic (Fig. 5b,d). Isoforms 1, 2 and 3 were more expressed in the dark, while Isoform 4 was cytosolic and its expression was constant. Isoforms 1, 2 and 3 of phosphoglycerate kinase (PGK) were chloroplastic and more expressed in the light, and Isoforms 4 and 5 were cytosolic and more expressed in the dark (Fig. 5b,d). Most of the 11 isoforms of glyceraldehyde‐3‐phosphate dehydrogenase (GAPDHC) were cytosolic and more expressed in the dark, except Isoform 7, which was chloroplastic and slightly more expressed in the light (Fig. 5b,d).

### Expression levels and location of proteins involved in biophysical CCMs

The transcript expression of a carbonic anhydrase, αCA1 and of a SoLute Carrier, SLC4, was similar in HC and LC plants. As previously described (Huang *et al*., 2020), four isoforms of αCA1 were periplasmic (Fig. S12). In addition, all seven isoforms of SLC4 were predicted to be in the plasmalemma, but subcellular proteomics failed to find SLC4 as it is embedded in the plasmalemma. We also identified three isoforms of a CA2‐like protein that were either chloroplastic or cytosolic based on prediction and subcellular proteomics. Isoforms 2 and 3 of αCA1, Isoform 1 of CA2‐like and Isoforms 4 and 6 of SLC4 were highly upregulated in the light.

All the transcript levels reported here as total count (RPKM) are also expressed as fold‐change in Fig. S2 and show a similar pattern.

### Distance between chloroplasts and mitochondria

The median distance between mitochondria and chloroplasts was 0.38 μm in HC and even closer, at 0.21 μm in LC (significantly different, *P* = 0.008, Wilcoxon test) (Fig. S13).

## Discussion

The unique challenges faced by aquatic plants to acquire inorganic carbon are ameliorated in many species by a biophysical CCM that accesses bicarbonate and in a few species by biochemical CCMs involving C_4_ and/or CAM (Maberly & Gontero, 2017, 2018). In most terrestrial plants with C_4_ metabolism, two cell types in the canonical Kranz anatomy are involved, but a few species perform this pathway within a single cell such as in the eudicot genera *Bienertia* and *Suaeda* (Voznesenskaya *et al*., 2001, 2002; Edwards *et al*., 2004). Similarly, C_4_ metabolism and CAM are not usually found in the same plant (Sage, 2002), but there are a few known exceptions such as several species within the genus *Portulaca*, plus *Trianthema portulacastrum, Sesuvium sesuvioides* and *S. littoreus* (Koch & Kennedy, 1980; Ho *et al*., 2019; Winter *et al*., 2021; Siadjeu & Kadereit, 2024). In freshwater C_4_ plants such as *H. verticillata* and *E. densa* (Casati *et al*., 2000; Bowes, 2011), carboxylation and decarboxylation can occur within one type of cell, the epidermis, because they lack mesophyll cells (Pendland, 1979; Hara *et al*., 2015). Mature leaves of *O. alismoides* do have mesophyll cells, although the upper and lower epidermis comprise *c*. 80% of the leaf blade cell area (Han *et al*., 2020). In this species, chloroplasts with grana and starch are present in both mesophyll and epidermal cells, unlike in plants with Kranz anatomy (Kanai & Edwards, 1999). However, it was impossible to distinguish between a dual‐cell or single‐cell model (Han *et al*., 2020). Nonetheless, since we show here that Rubisco is present in both cell types, strengthening the likelihood of a single‐cell C_4_ system in *O. alismoides* as in the single‐cell C_4_ species mentioned above (Table S3).

In the terrestrial single‐cell C_4_ plants *B*. *sinuspersici* and *S. aralocaspica*, mitochondria and chloroplasts are nearby, either in the centre or at the proximal end of the cell (Voznesenskaya *et al*., 2002; Edwards *et al*., 2004). In *O. alismoides*, the median distance of mitochondria and chloroplasts was shorter in LC than in HC plants (Fig. S13). The proximity in a single cell of sites of decarboxylation in mitochondria and carboxylation by Rubisco in chloroplasts will facilitate the exchange of CO_2_ and metabolites (Raghavendra & Padmasree, 2003).

**Fig. 6:**
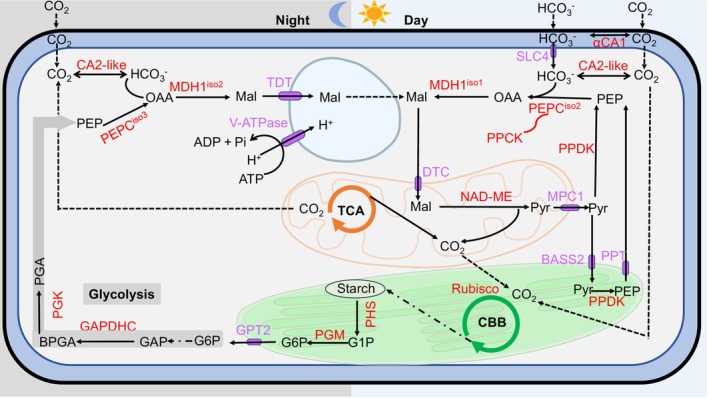
A model of the coexistence of C_4_, crassulacean acid metabolism (CAM), and bicarbonate use in a single cell of *Ottelia alismoides*. The model is for a plant grown at low CO_2_ in which CAM is activated and shows processes in the day and night. The cell wall and plasma membrane are shown by a black line and a blue line respectively, and the intervening periplasmic space is shown in blue. The tricarboxylic cycle is located in a mitochondrion (orange), the Calvin–Benson–Bassham (CBB) cycle in a chloroplast (green), and glycolysis in the cytoplasm (grey arrows). Reactions are shown by a solid line, diffusion between compartments by a dashed line, membrane transporters are in purple, enzymes are in red and chemicals are in black. Transporter and enzyme names are: bile acid sodium symporter (BASS2), carbonic anhydrase (CA), dicarboxylate/tricarboxylate transporter (DTC), glyceraldehyde‐3‐phosphate dehydrogenase (GAPDHC), glucose‐6‐phosphate translocator (GPT2), malate dehydrogenase (MDH), mitochondrial pyruvate carrier (MPC1), NAD malic enzyme (NAD‐ME), PEP carboxylase (PEPC), phosphoglucomutase (PGM), starch phosphorylase (PHS), PEPC kinase (PPCK), phosphoenolpyruvate transporter (PPT), phosphoglycerate kinase (PGK), pyruvate phosphate dikinase (PPDK), ribulose bisphosphate carboxylase‐oxygenase (Rubisco), solute carrier family 4 (SLC4), tonoplast dicarboxylate transporter (TDT), vacuolar‐ATPase (V‐ATPase). Metabolites are: Bisphosphoglycerate (BPGA), glyceraldehyde‐3‐phosphate (GAP), glucose‐1‐phosphate (G1P), glucose‐6‐phosphate (G6P), malate (Mal), oxaloacetate (OAA), phosphoenolpyruvate (PEP), phosphoglycerate (PGA), pyruvate (Pyr). Information for CA and SLC4 is based on previous data (Huang *et al*., 2020) and data in Supporting Information Fig. S12.

The data presented here and previously (Zhang *et al*., 2014; Shao *et al*., 2017; Han *et al*., 2020) were used to construct a model showing how C_4_ and bicarbonate use during the day, and CAM at night, could be integrated within a single cell (Fig. 6). Previous work showed that in the light, external bicarbonate was supplied to epidermal cells via a periplasmic αCA1 and SLC4 embedded in the plasma membrane (Fig. S12) (Huang *et al*., 2020). The uptake and assimilation of bicarbonate can increase cellular pH as hydroxide ions are formed when CO_2_ is fixed. In the seagrass *Posidonia oceanica*, bicarbonate uptake involves a symport with protons that maintains a pH balance (Rubio *et al*., 2017). In freshwater photoautotrophs, bicarbonate uptake and proton or hydroxide exchange are often spatially separated. For example, in the green macroalga *Chara braunii*, acid and alkaline bands occur around the large internodal cells (Heise *et al*., 2025), while many angiosperms that use bicarbonate possess ‘polar leaves’ with a high pH at the adaxial (upper) leaf surface and a low pH at the abaxial (lower) surface (Prins & Elzenga, 1989). We observed calcite precipitation on the adaxial leaf surface of *O. alismoides*, which is consistent with a polar leaf, but further work is needed. *O. alismoides* grown at LC generated a high pH in the culture medium and reduced the alkalinity. This is consistent with calcite precipitation that also has biogeochemical consequences including coprecipitation of phosphate (Otsuki & Wetzel, 1972).

In the C_4_ pathway of *O. alismoides*, the internal bicarbonate is converted to malate through the consecutive action of the cytosolic enzymes PEPC isoform 2 and MDH1 isoform 1. Our transcriptomic and transcriptomic data indeed indicate that the PEPC transcripts at the end of the day and the end of the night are produced in anticipation of the circadian cycle and that Isoform 2 is likely to be involved in C_4_ and Isoform 3 is likely to be involved in CAM. In terrestrial C_4_ plants, PEPC is phosphorylated in the the light to prevent malate inhibition (Jiao & Chollet, 1991; Chollet *et al*., 1996). In *O. alismoides*, phosphorylation of PEPC occurs, likely by PPCK, in both the day and the night (Table S2), thereby preventing malate inhibition during daytime C_4_ and night‐time CAM. However, PEPC was not phosphorylated at 07:30, 30 min before the end of the dark period and PEPC activity was low, suggesting that post‐translation control is one of the factors that regulate CAM activity as found for other CAM species (Theng *et al*., 2015). Furthermore, at night malate is moved effectively from the cytoplasm into the vacuole by a tonoplast TDT (Wai *et al*., 2017; Ceusters *et al*., 2021) that, along with a V‐ATPase, is strongly upregulated in LC plants at night and is extremely low in HC plants (Fig. 6). In addition, the amount of malate produced in the night by *O. alismoides* is lower than in many other CAM plants (Gontero & Maberly, 2022) minimizing possible inhibition. Finally, GPT2 can activate PEPC (Munoz‐Clares *et al*., 2020) and the concentration of this metabolite in *O. alismoides* was high in LC plants at night (Huang *et al*., 2022).

The PEPC that performs CAM in the freshwater lycopod *Isoetes taiwanensis* (Wickell *et al*., 2021) is similar to the bacterial‐type ppc4 found in *Arabidopsis thaliana* (Sanchez & Cejudo, 2003). PEPC isoform 1 from *O. alismoides* (Fig. S10; Table S1) is also similar to the bacterial type, but is weakly expressed, not phosphorylated (Table S2), and is unlikely to be involved in CAM or C_4_, while PEPC isoform 3 is involved in CAM, as mentioned above.

The substrate of PEPC, PEP, is produced by different pathways in C_4_ and CAM. In C_4_ metabolism during the day, PEP is provided by PPDK (Kanai & Edwards, 1999). In *O. alismoides*, PPDK is present in both the chloroplast and cytosol (Fig. 6) as has been described previously (Kondo *et al*., 2000; Moreno‐Villena *et al*., 2022). Thus, the pyruvate produced by NAD‐ME can be exported by MPC1 to the cytosol, in which it can be converted to PEP by the cytosolic PPDK. Additionally, the cytosolic pyruvate can be imported into the chloroplast through the highly expressed BASS2 (Furumoto, 2016) and converted by the chloroplastic PPDK into PEP. This is exported to the cytosol by the highly expressed Isoform 4 of PPT (Weber & von Caemmerer, 2010), in which it can be utilized by PEPC (Fig. 6). In CAM plants at night, PEP can be provided by the phosphorylytic degradation of starch by a chloroplastic phosphorylase (Ceusters *et al*., 2019). This involves PGM, GPT2 and two cytosolic glycolytic enzymes: PGK and GAPDHC (Fig. 6). Our results are consistent with this pathway in *O. alismoides* since all these enzymes are most highly expressed at night in LC plants, but this proposed route needs to be confirmed experimentally.

In the canonical C_4_ NAD‐ME subtype, aspartate produced from OAA by AspAT is the main initial product (Kanai & Edwards, 1999). In NAD‐ME C_4_ plants with Kranz anatomy, AspAT and AlaAT are the major components of a shuttle linking the metabolism between bundle sheath and mesophyll cells in order to balance nitrogen and reducing power (Weber & von Caemmerer, 2010; Furbank, 2011; Rao & Dixon, 2016; Moreno‐Villena *et al*., 2022). This shuttle may not be necessary in single‐cell C_4_ systems. Although *O. alismoides* belongs to the NAD‐ME subtype, ^13^C labelling and activity measurements showed, unexpectedly, that malate, not aspartate, was the main initial product. OAA can also be converted into malate by MDH1 that occurred in the cytosol in which PEPC is located, while AspAT only occurred in the mitochondria and chloroplasts (Fig. 3). MDH1 isoform 1 could convert OAA into malate for C_4_ photosynthesis in the light, while Isoform 2 could be the counterpart for CAM in the dark. The ^13^C labelling, enzyme activity, and location all suggest that aspartate does not play a major role in C_4_ photosynthesis in *O. alismoides*. The malate produced by MDH1 isoform 1 is transported into the mitochondria by DTC that is highly expressed during the day and is then decarboxylated by NAD‐ME. Thus, the co‐occurrence of C_4_ and CAM within the same cell in *O. alismoides* is enabled by the opposite temporal expression of TDT and V‐ATPase at night and DTC during the day and the temporal expression and regulation of different isoforms.

From an ecological viewpoint, the presence of three CCMs in *O. alismoides* provides the flexibility to maximize inorganic carbon acquisition in an environment where this resource is limiting and/or fluctuating. During the day, the reserves of bicarbonate can be accessed, allowing photosynthesis to continue when the external concentration of CO_2_ is very low. C_4_ metabolism, initiated by the fixation of bicarbonate by PEPC, concentrates CO_2_ around Rubisco, minimizing photorespiration (Monson *et al*., 2025), especially relevant since high‐oxygen concentrations are associated with low CO_2_ concentrations. During the night, CAM reduces the loss of respiratory CO_2_ by temporarily fixing it into malate and storing it in the vacuole. It also has the potential to take up external CO_2_ when concentrations are high because of community respiration, as occurs in the freshwater CAM plant *Crassula helmsii* (Klavsen & Maberly, 2009). Bicarbonate use is likely to occur mainly or uniquely in epidermal cells, but the contribution of the two other CCMs to carbon fixation in epidermal and mesophyll cells is a challenge for planned future work.

From a diversity viewpoint, knowledge on the variety of structures and biochemistry involved in CCMs has steadily increased. C_4_ photosynthesis is not restricted to plants with Kranz anatomy, with the discovery of single‐cell C_4_ in terrestrial (Edwards *et al*., 2004) and freshwater plants (Bowes, 2011) and possibly in the marine diatom *Thalassiosira weissflogii* (Reinfelder *et al*., 2000). Furthermore, carbon fixation by PEP carboxykinase occurs in the marine macroalga *Udotea flabellum* (Reiskind & Bowes, 1991). Despite it being thought to be impossible for biochemical, anatomical, and evolutionary reasons (Sage, 2002), C_4_ and CAM co‐occur in the same tissue in some species as mentioned previously (Guralnick *et al*., 2002; Ho *et al*., 2019; Winter *et al*., 2021; Siadjeu & Kadereit, 2024; Huang *et al*., 2025). *O. alismoides* increases the known types of C_4_ further since it operates a single‐cell NAD‐ME subtype relying on malate rather than aspartate and combines this with CAM and bicarbonate use (Fig. 6). The possibility that NAD‐ME relies on malate rather than aspartate in terrestrial single‐cell C_4_ plants has not been rigorously checked. The appreciation of the diversity of ways in which plants increase their productivity provides more options for the design of engineered crop plants to increase food security.

## Competing interests

None declared.

## Author contributions

HSJ, WH, JD, BG, SCM and WL conceived and designed the study; HSJ, WH, SH, PL, ZL, LW, SG and LZ performed the experiments; HSJ, BG and SCM analysed the data and produced the figures with help from all the authors; HSJ, BG and SCM led the writing of the manuscript with contributions from all the authors. HSJ and WH contributed equally to this work.

## Disclaimer

The New Phytologist Foundation remains neutral with regard to jurisdictional claims in maps and in any institutional affiliations.

## Supporting information


**Fig. S1** Inorganic carbon conditions in the culture medium.
**Fig. S2** Transcript levels expressed as change‐fold.
**Fig. S3** Photomicrographs of purified organelles from *Ottelia alismoides*.
**Fig. S4** Location of carboxylating and decarboxylating enzymes in *Ottelia alismoide*s grown at low carbon.
**Fig. S5** Effects of light and CO_2_ concentration on differential gene expression in *Ottelia alismoides*.
**Fig. S6** Gene Ontology enrichment of differentially expressed genes (DEGs).
**Fig. S7** Expression level of transcripts of isoforms of PRK and the subunits A and B of the chloroplastic GAPDH (A_2_B_2_).
**Fig. S8** Effects of inhibitors on photosynthesis in *Ottelia alismoides* and *Cabomba caroliniana* grown at low (LC) and high (HC) inorganic carbon.
**Fig. S9** Diel acidity change in *Ottelia alismoides*.
**Fig. S10** Phylogenetic tree of PEPC sequences from *Arabidopsis thaliana* and *Ottelia alismoides*.
**Fig. S11** Time course of ^13^C enrichment (%) for key C_3_ and C_4_ metabolites in *Cabomba caroliniana* and *Ottelia alismoides* grown at low (LC) and high (HC) inorganic carbon.
**Fig. S12** Expression levels and location of proteins involved in inorganic carbon uptake in *Ottelia alismoides*.
**Fig. S13** Distance between chloroplasts and mitochondria in *Ottelia alismoides*.
**Table S1** Amino acid sequences of proteins derived from transcriptomic data used in this paper on *Ottelia alismoides*.

**Table S2** Phosphorylation states of PEPC isoforms at different times in *Ottelia alismoides*.
**Table S3** Comparison of biochemical CO_2_ concentrating mechanisms in leaves of aquatic and terrestrial plants.
Please note: Wiley is not responsible for the content or functionality of any Supporting Information supplied by the authors. Any queries (other than missing material) should be directed to the *New Phytologist* Central Office.

## Data Availability

All data are available in the main text, the Supporting Information or in the CNGB (https://db.cngb.org/): CNP0007412, CNP0007421.
