# Supplementary material for: A novel single‐cell NAD‐ME C4 subtype integrated with CAM and bicarbonate use in an aquatic plant
Source: New Phytol. 2025 Oct 21;249(5):2386–401. doi: 10.1111/nph.70673 (PMC12873514; doi:10.1111/nph.70673)
Supplement: Supplementary file 1 — Fig. S1 Inorganic carbon conditions in the culture medium. Fig. S2 Transcript levels expressed as change‐fold. Fig. S3 Photomicrographs of purified organelles from Ottelia alismoides. Fig. S4 Location of carboxylating and decarboxylating enzymes in Ottelia alismoides grown at low carbon. Fig. S5 Effects of light and CO2 concentration on differential gene expression in Ottelia alismoides. Fig. S6 Gene Ontology enrichment of differentially expressed genes (DEGs). Fig. S7 Expression level of transcripts of isoforms of PRK and the subunits A and B of the chloroplastic GAPDH (A2B2). Fig. S8 Effects of inhibitors on photosynthesis in Ottelia alismoides and Cabomba caroliniana grown at low (LC) and high (HC) inorganic carbon. Fig. S9 Diel acidity change in Ottelia alismoides. Fig. S10 Phylogenetic tree of PEPC sequences from Arabidopsis thaliana and Ottelia alismoides. Fig. S11 Time course of 13C enrichment (%) for key C3 and C4 metabolites in Cabomba caroliniana and Ottelia alismoides grown at low (LC) and high (HC) inorganic carbon. Fig. S12 Expression levels and location of proteins involved in inorganic carbon uptake in Ottelia alismoides. Fig. S13 Distance between chloroplasts and mitochondria in Ottelia alismoides. Table S1 Amino acid sequences of proteins derived from transcriptomic data used in this paper on Ottelia alismoides. [file NPH-249-2386-s001.docx]

## *New Phytologist* Supporting Information

Article title: **A novel single-cell NAD-ME C_4_ subtype integrated with CAM and bicarbonate use in an aquatic plant**

Authors: Hong Sheng Jiang, Wenmin Huang, Shijuan Han, Pengpeng Li, Zuying Liao, Liyuan Wei, Lei Zhao, Shuping Gu, Jun Ding, Brigitte Gontero, Stephen C. Maberly, Wei Li

Article acceptance date: 30 September 2025

The following Supporting Information is available for this article:

**Fig. S1 Inorganic carbon conditions in the culture medium.**

**Fig. S2 Transcript levels expressed as change-fold.**

**Fig. S3. Photomicrographs of purified organelles from *Ottelia alismoides*.**

**Fig. S4 Location of carboxylating and decarboxylating enzymes in *Ottelia alismoide*s grown at low carbon.**

**Fig. S5 Effects of light and CO_2_ concentration on differential gene expression in *Ottelia alismoides*.**

**Fig. S6 Gene Ontology enrichment of Differentially Expressed Genes (DEGs).**

**Fig. S7 Expression level of transcripts of isoforms of PRK and the subunits A and B of the chloroplastic GAPDH (A_2_B_2_).**

**Fig. S8 Effects of inhibitors on photosynthesis in *Ottelia alismoides* and *Cabomba caroliniana* grown at low (LC) and high (HC) inorganic carbon.**

**Fig. S9 Diel acidity change in *Ottelia alismoides*.**

**Fig. S10 Phylogenetic tree of PEPC sequences from *Arabidopsis thaliana* and *Ottelia alismoides*.**

**Fig. S11 Time course of ^13^C enrichment (%) for key C_3_ and C_4_ metabolites in *Cabomba caroliniana* and *Ottelia alismoides* grown at low (LC) and high (HC) inorganic carbon.**

**Fig. S12 Expression levels and location of proteins involved in inorganic carbon uptake in *Ottelia alismoides*.**

**Fig. S13 Distance between chloroplasts and mitochondria in *Ottelia alismoides*.**

**Table S1. Amino acid sequences of proteins derived from transcriptomic data used in this paper on *Ottelia alismoides.***

**Table S2.** P**hosphorylation states of PEPC isoforms at different times in *Ottelia alismoides*.**

**Table S3. Comparison of biochemical CO_2_ concentrating mechanisms in leaves of aquatic and terrestrial plants.**

**Fig. S1. Inorganic carbon conditions in the culture medium.** (**a**), pH; (**b**), Alkalinity; (**c**), Concentration of CO_2_. Samples were collected in the dark at the end of the night (07:45) and in the light at the end of the day (21:40). Error bars represent the standard deviation for n = 5 for low inorganic carbon (LC) and n= 6 for high inorganic carbon (HC).

**
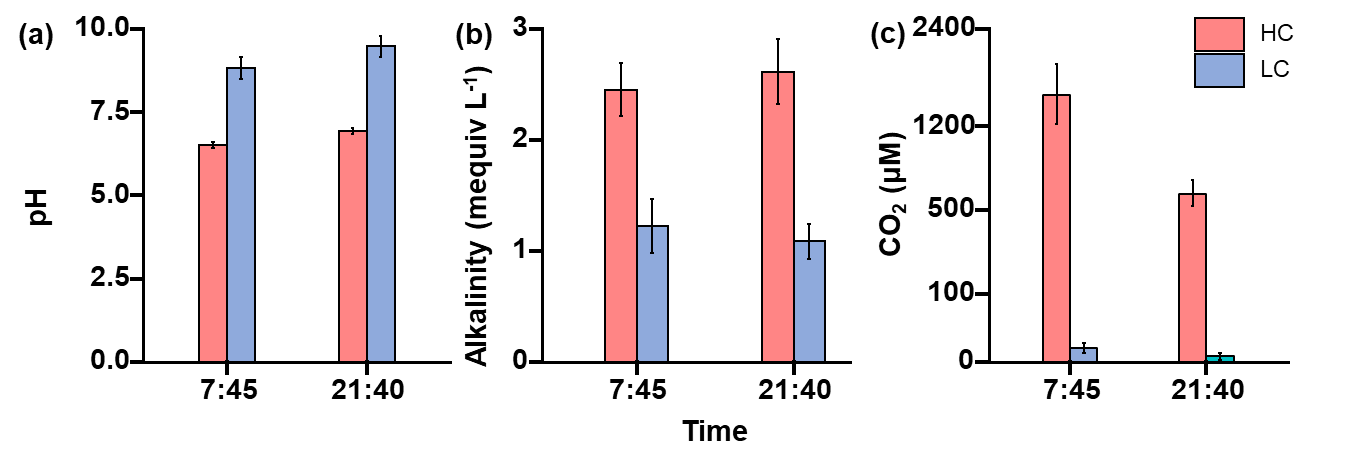
**

**Fig. S2. Transcript levels expressed as change fold.** Heatmap of transcript levels (change-fold) of plants grown at high carbon (HC) or low carbon (LC) and collected in the dark (D) or light (L). Statistically significant differences using a Generalised Linear Model and an ANOVA (p< 0.05) among samples are indicated by different letters.

**
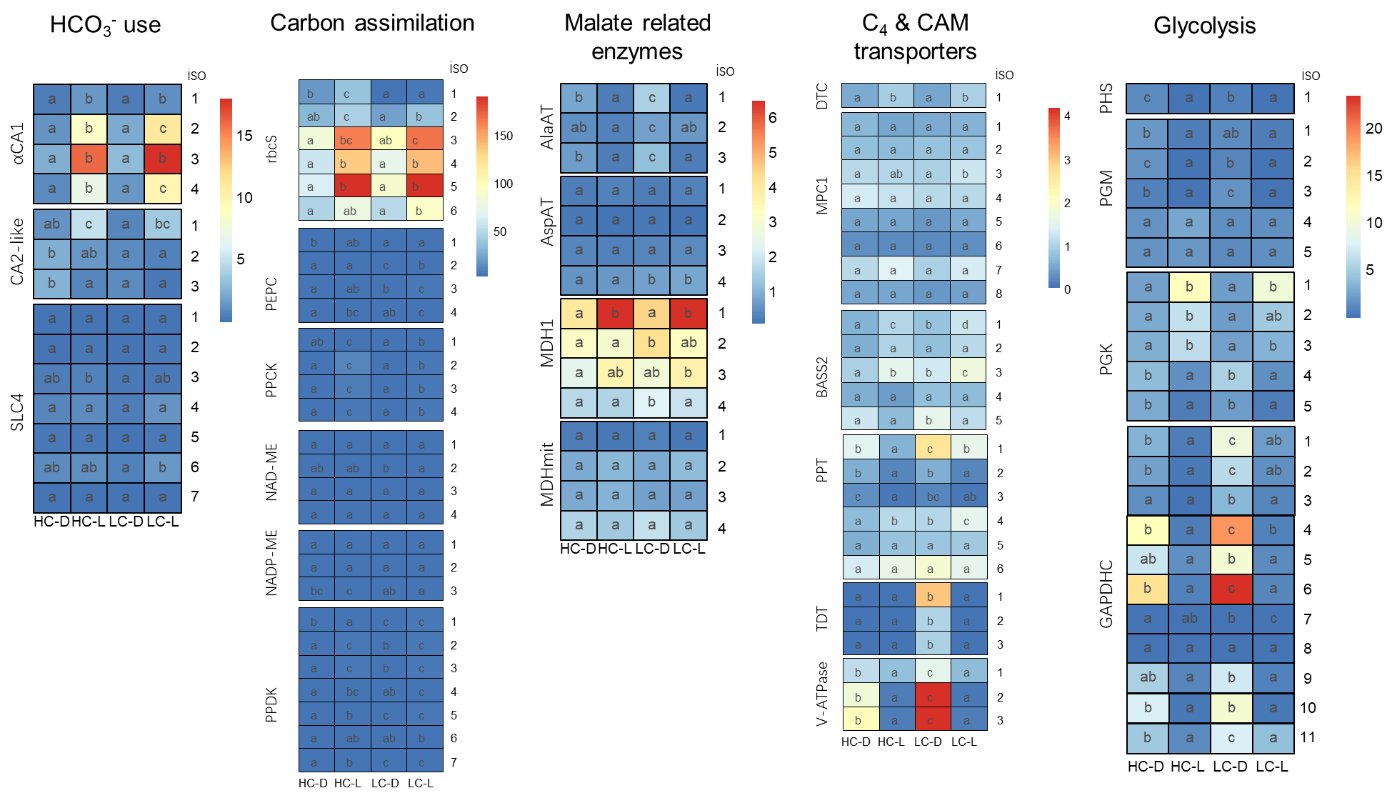
**

**Fig. S3. Photomicrographs of purified organelles from *Ottelia alismoides***. (**a**) chloroplasts and (**b**), mitochondria. Plant material was that used in the measurements in Figs **1,3,5**, and Fig. S4 and S12. Mitochondria were stained with Janus green B. The black scale bars are 50 µm and the red scale bars in the insets are 5 µm for plants grown at low inorganic carbon.


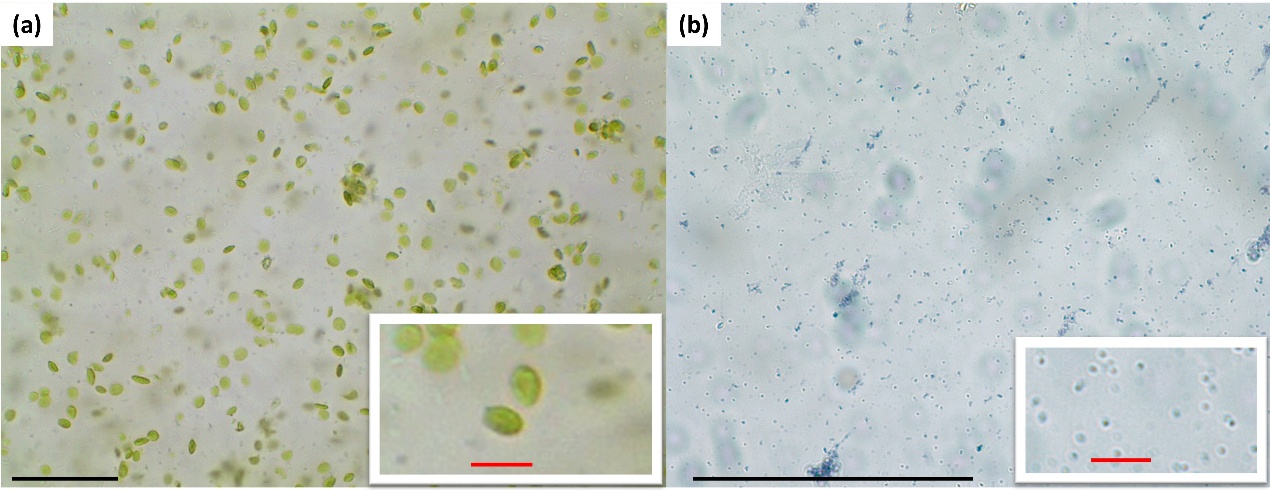


**Fig. S4. Location of carboxylating and decarboxylating enzymes in *Ottelia alismoide*s grown at inorganic carbon**. (**a**) Western blot of the large subunit of Rubisco (molecular mass ~55 kDa); (**b**) Western blot of PEPC (molecular mass ~110 kDa; (**c**) Western blot of NAD-ME (molecular mass ~70 kDa); m1 to 6, c1 to 5 and L1 to 3 are extracts from purified mitochondria, purified chloroplasts and leaves, respectively; (**d**) Transmission electronic microscopy images of chloroplasts from the lower epidermal cells, mesophyll cells and upper epidermal cells, labelled with immunogold with an antibody raised against the large subunit of Rubisco (black dots). The scale bar is 1 µm. The purified chloroplasts from c5 were not used for further experiments as they appeared to be contaminated with PEPC.


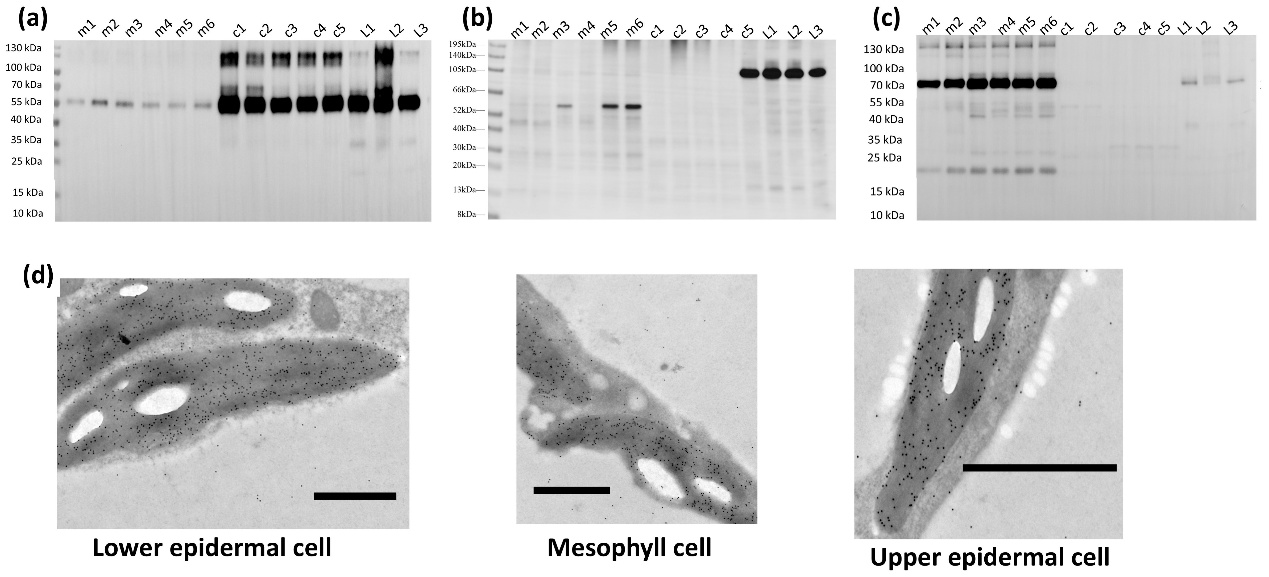


**Fig. S5.** **Effects of light and CO_2_ concentration on differential gene expression in *Ottelia alismoides*.** (**a**) PCA of the number of differentially expressed genes (DEGs). **(b)** DEGs in light (L) *vs* dark (D) in plants grown in low (LC) or high (HC) carbon, **(c)** DEGs in LC *vs* HC in plants collected in L or D.


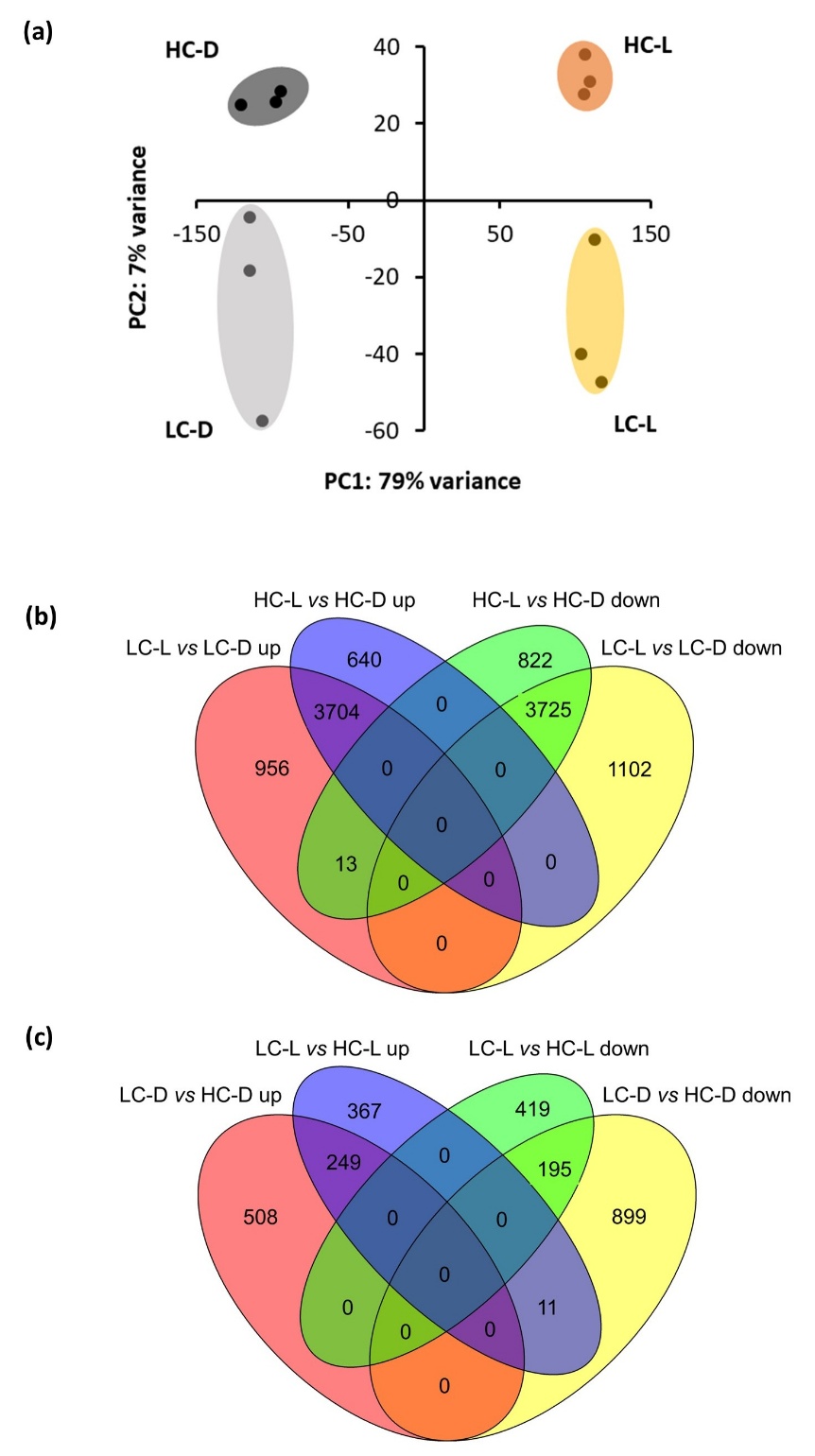


**Fig. S6. Gene Ontology enrichment of Differentially Expressed Genes (DEGs)**. (**a**) Down regulation of DEGs in HC-L compared to HC-D; (**b**) Up regulation of DEGs in HC-L compared to HC-D; (**c**) Down regulation of DEGs in LC-D compared to HC-D; (**d**) Up regulation of DEGs in LC-D compared to HC-D; (**e**) Down regulation of DEGs in LC-L compared to HC-L; (**f**) Up regulation of DEGs in LC-L compared to HC-L; (**g**) Down regulation of DEGs in LC-L compared to LC-D; (**h**) Up regulation of DEGs in LC-L compared to LC-D. BP: Biological Process; CC: Cellular Component; MF: Molecular Function. The pale green items indicate C_4_ photosynthesis and orange pale items indicate CAM.


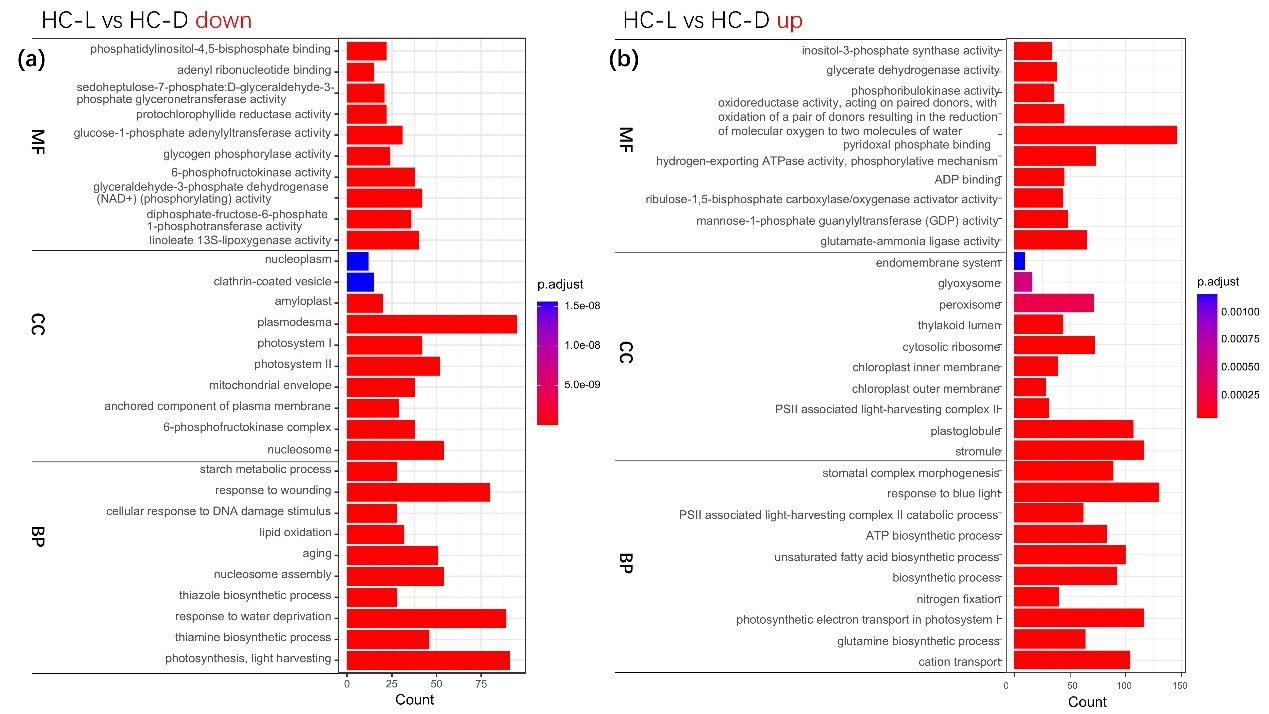


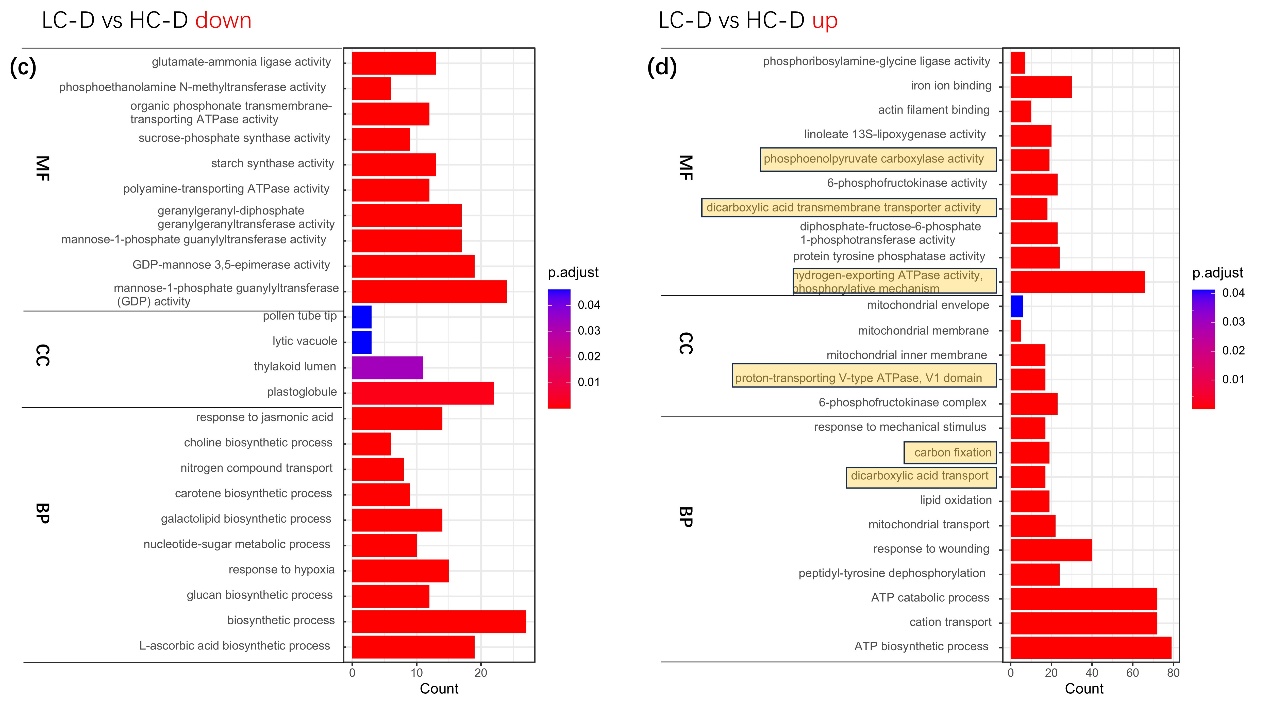


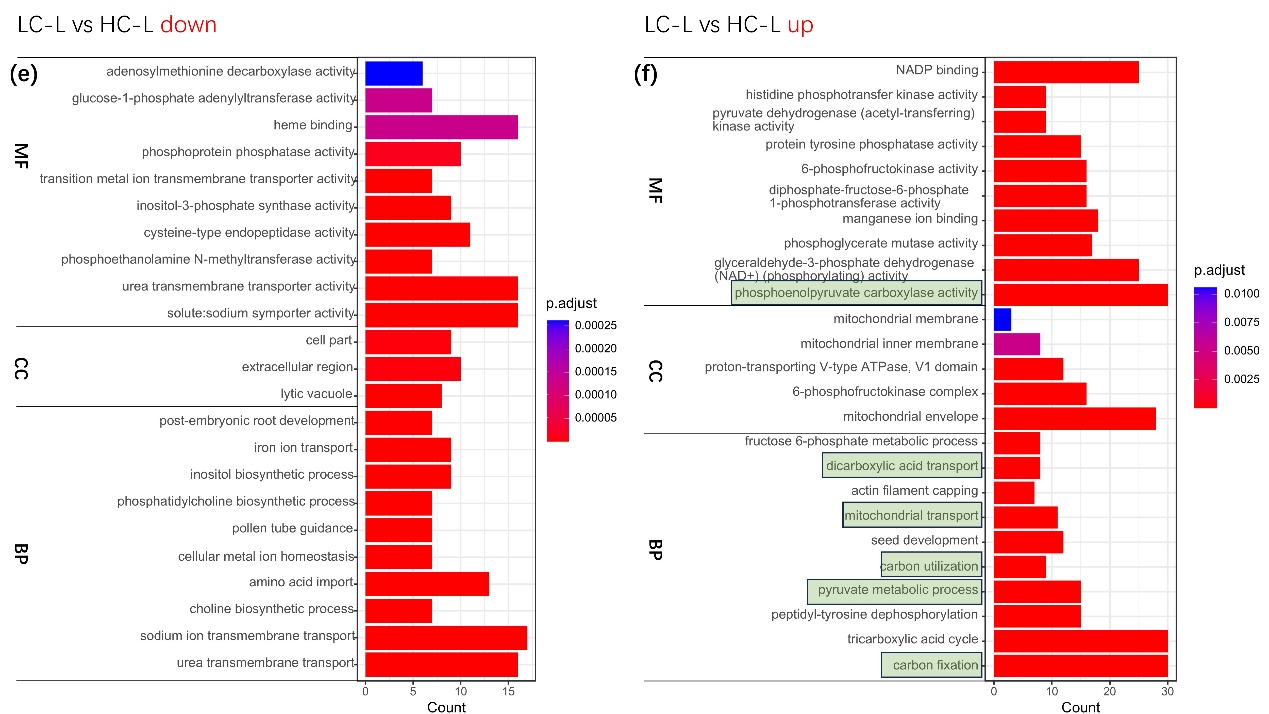


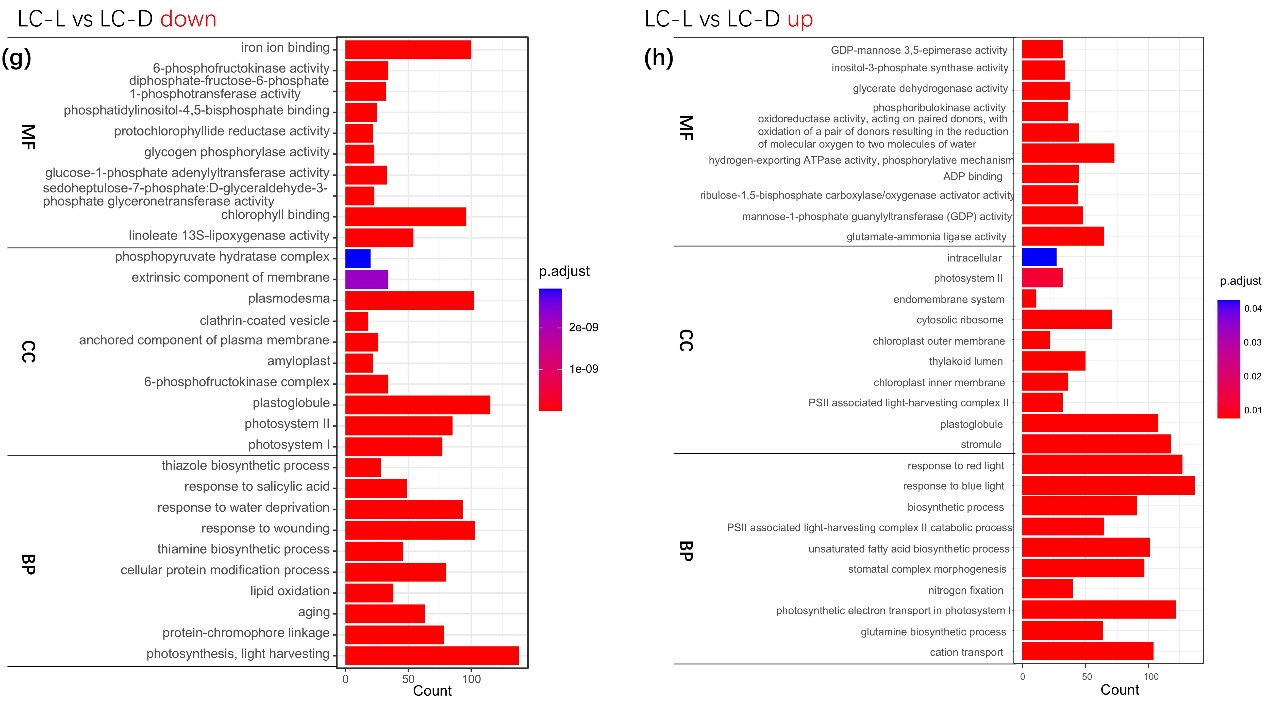


**Fig. S7. Expression** **level of transcripts of isoforms of PRK and the subunits A and B of the chloroplastic GAPDH (A_2_B_2_).** *Ottelia alismoides* was grown at high inorganic carbon (HC) or low inorganic carbon (LC) and material was collected in the dark (D) or light (L). The error bars show one standard deviation and statistically significant differences using a Generalised Linear Model and an ANOVA are shown by letters (p<0.05).


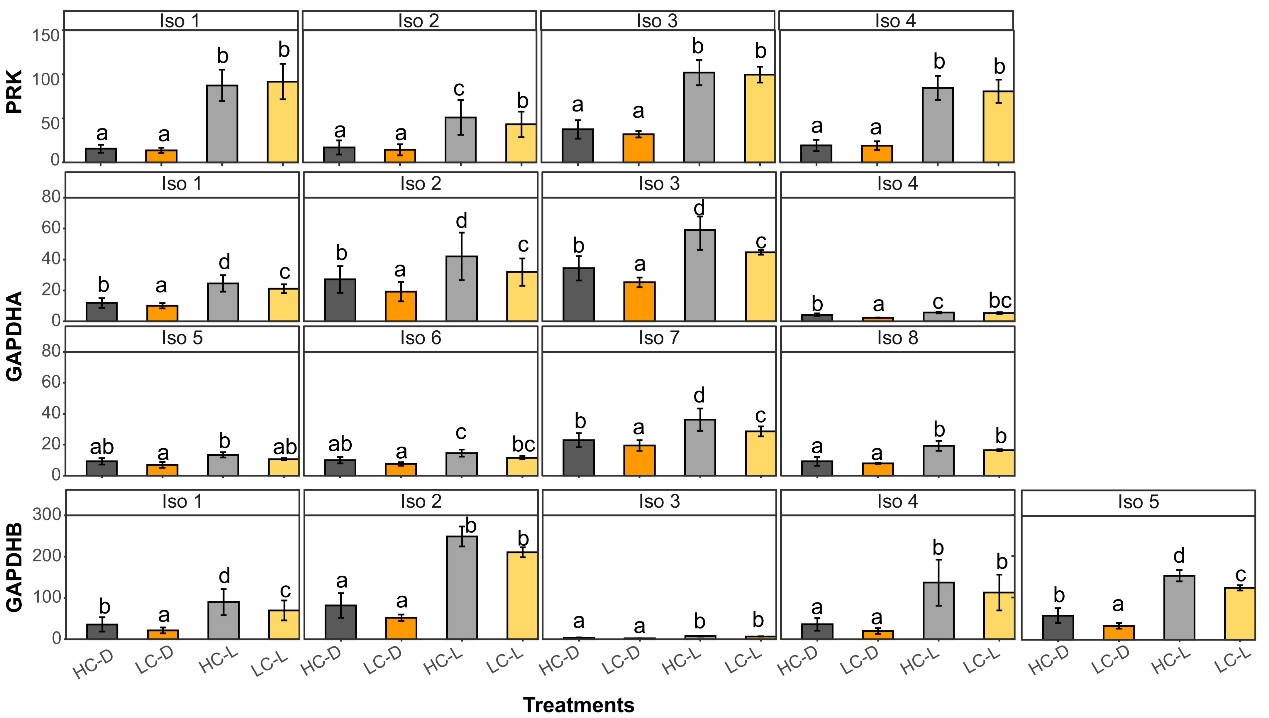


**Fig. S8. Effects of inhibitors on photosynthesis in *Ottelia alismoides* and** ***Cabomba caroliniana* grown at low (LC) and high (HC) inorganic carbon**. (a) Effect of BIM4 (bisindolylmaleimide IV), a PPDK inhibitor, and okanin, a PEPC inhibitor, relative to the control; For (**a**), the absolute rates ranged between 48 and 69 µmol g^-1^ fresh weight h^-1^ and each point was the mean of 3 to 6 values shown with one standard deviation; (**b**) The concentration of DMSO used (0.2%) corresponded to the DMSO concentration at the highest inhibitor concentration. Results are the mean and standard deviation of three replicates. Statistical significance *vs* control are calculated using a t-test. The R^2^ values for the fit to the data were between 0.75 and 0.94 for *C. caroliniana* and between 0.92 and 0.98 for *O. alismoides*.


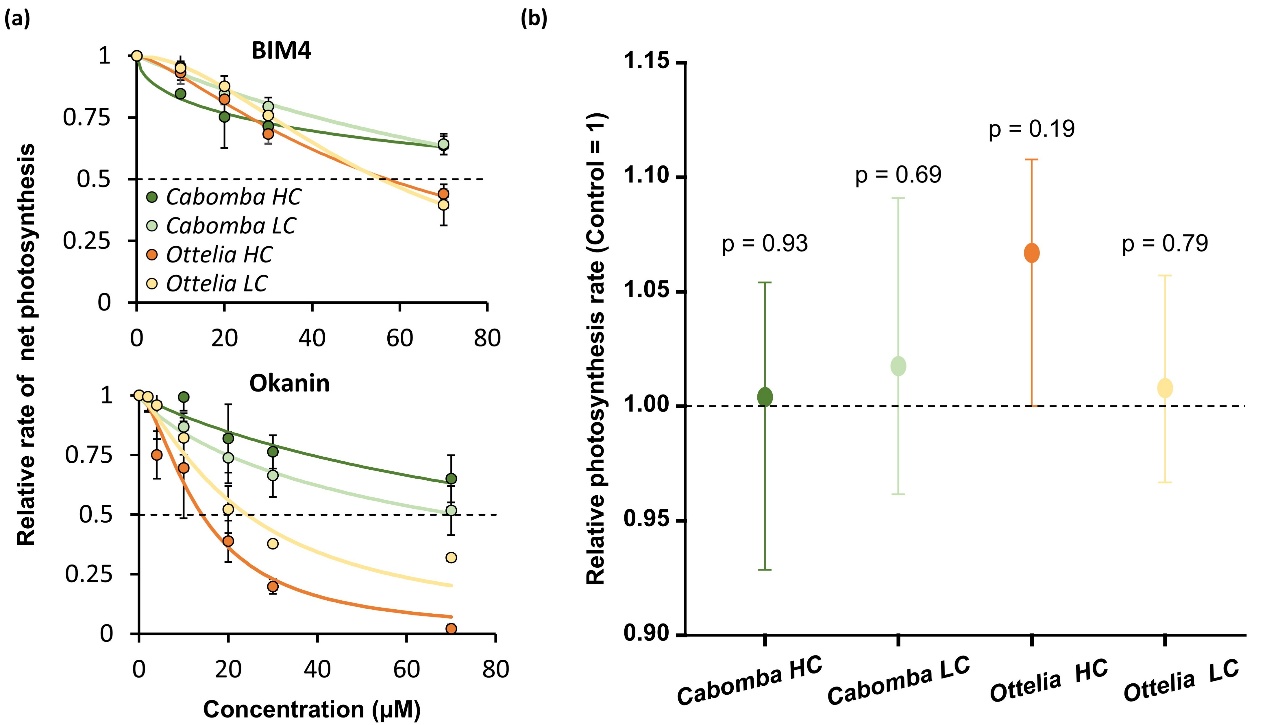


**Fig. S9. Diel acidity change in *Ottelia alismoides*.** The growth conditions are high inorganic carbon in the dark (HC-D), high inorganic carbon in the light (HC-L), low inorganic carbon in the dark (LC-D) and low inorganic carbon in the light (LC-L). Error bars represent the standard deviation for n = 4 - 6. Statistical differences were calculated using a linear mixed model. ns, not significant; * = p<0.05, ** = p<0.01.


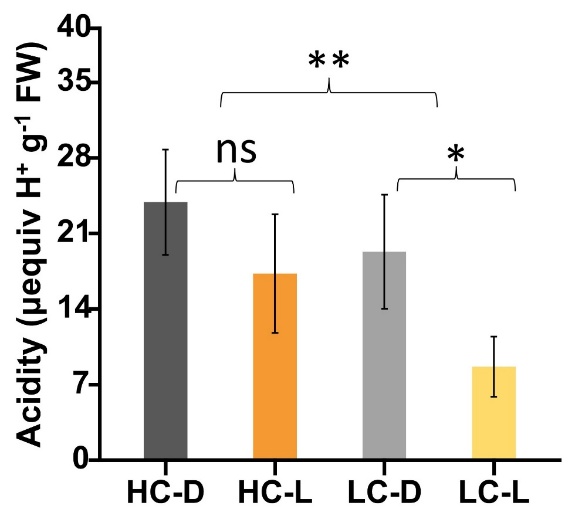


**Fig. 10 Phylogenetic tree of PEPC sequences from *Arabidopsis thaliana* and *Ottelia alismoides*.** PEPC1-4 (AtPPC1-4 accession number: sp|Q8GVE8.1, sp|Q5GM68.2, sp|Q84VW9.2, sp|Q9MAH0.1) sequences of *A. thaliana* were obtained from the NCBI database and PEPC1-4 sequences of *O. alismoides* (Ott_ali_PPC1-4) were obtained in this work (Table S1). Eight sequences were aligned using MUSCLE software (https://www.ebi.ac.uk/Tools/msa/muscle/) and a Neighbor-Joining phylogenetic tree was constructed using a p-distance model. The robustness of each branch was estimated using 1000 bootstrap analysis.


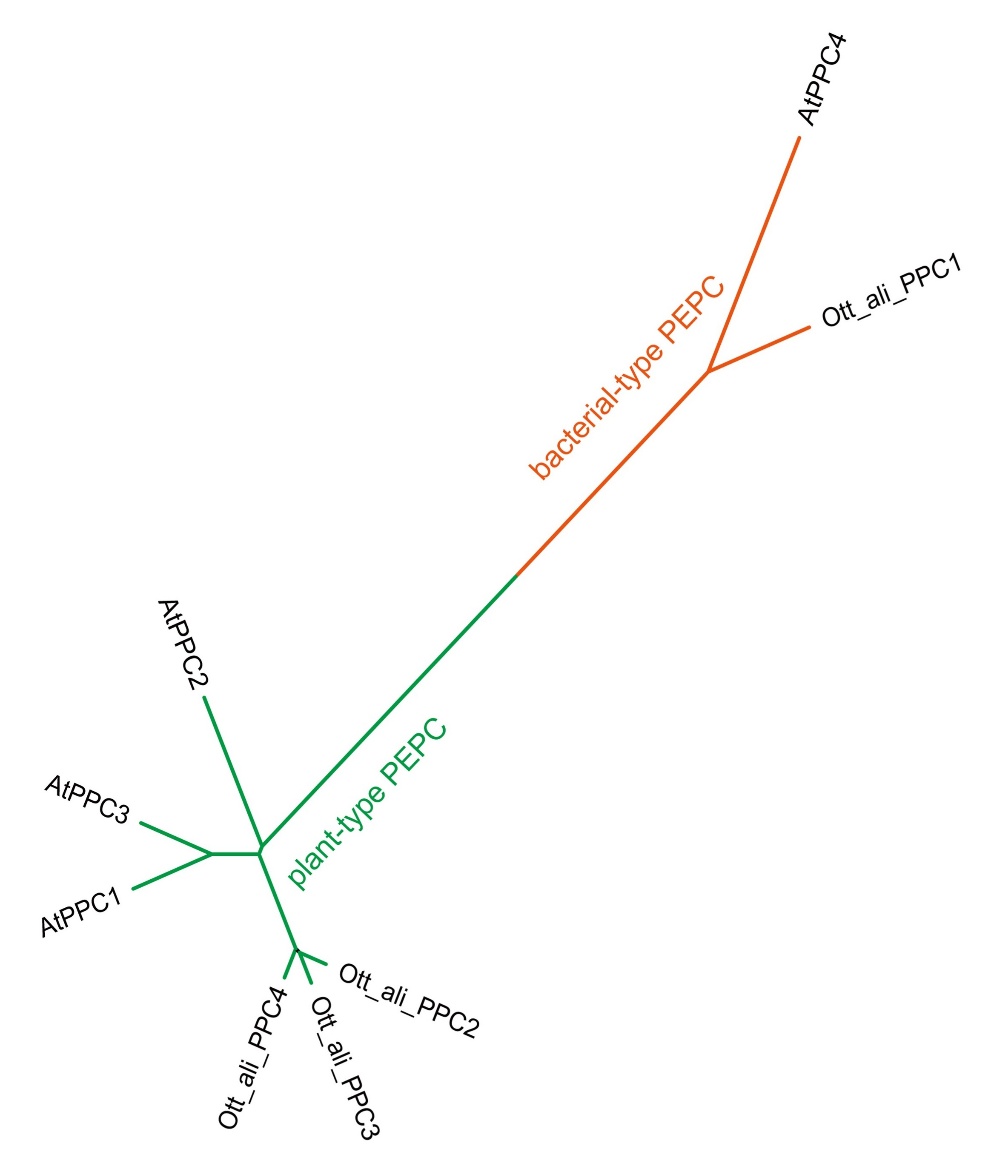


**Fig. S11. Time course of ^13^C enrichment (%) for key C_3_ and C_4_ metabolites in *Cabomba caroliniana* and *Ottelia alismoides* grown at low (LC) and high (HC) inorganic carbon**. Values are the mean of three replicates with one standard deviation. MAL (Malate), PGA (3-phosphoglycerate), ASP (Aspartate), ALA (Alanine), PYR (Pyruvate).


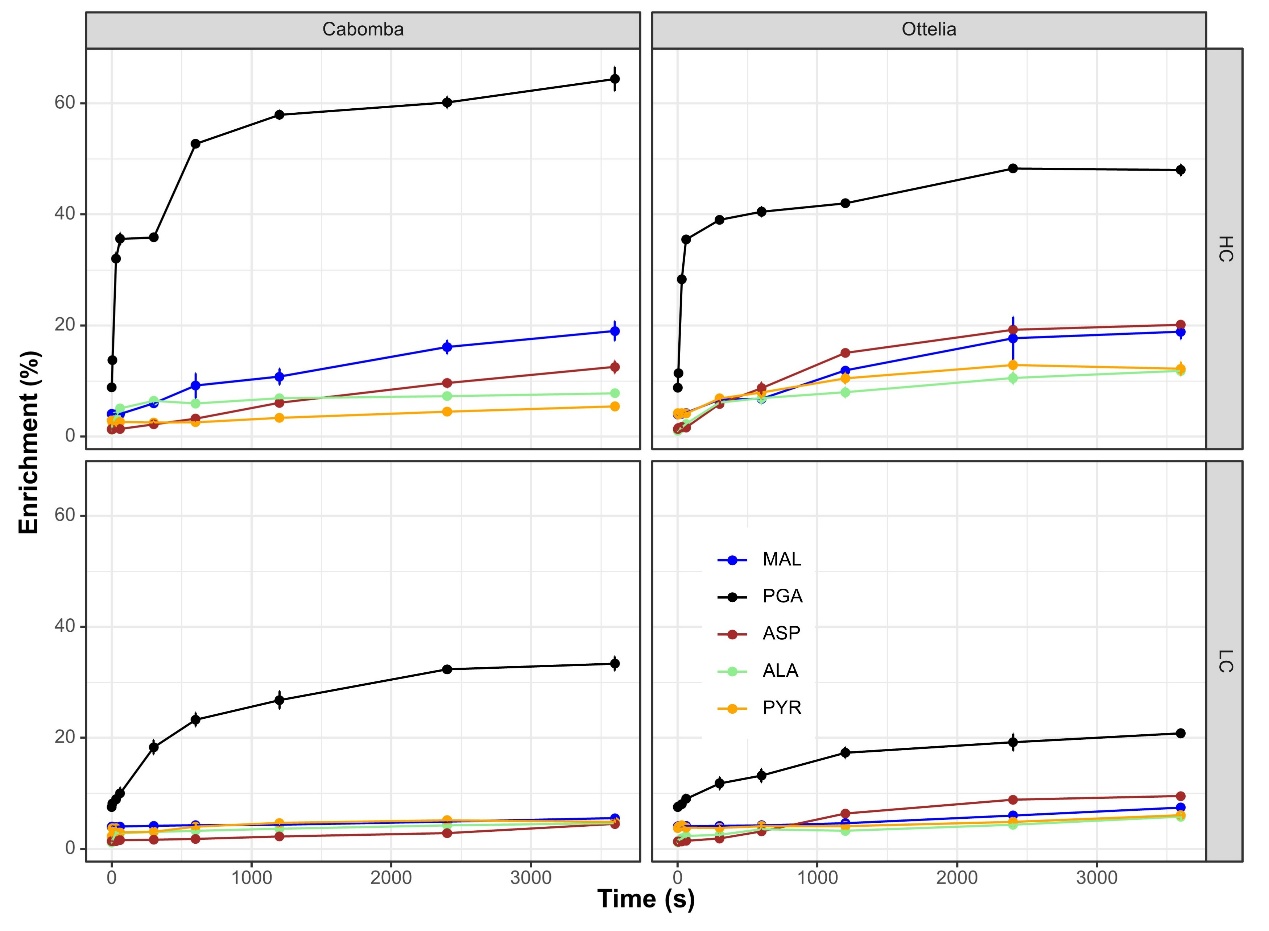


**Fig. S12. Expression levels and location of proteins involved in inorganic carbon uptake in *Ottelia alismoides*.** (**a**) Heatmap of protein transcript levels with significant differences shown by letters (P <0.05) of plants gown at high inorganic carbon (HC) or low inorganic carbon (LC) and collected in the dark (D) or light (L); (**b**) Relative abundance of αCA1 and CA2-like isoforms (iBAQ) from leaf extracts, purified chloroplasts (CP) and mitochondria (Mit). The insert corresponds to a zoom of the relative abundance of isoform 3. Values are the mean of three replicates with one standard deviation. Different lowercase letters in panels b and c indicate significance calculated using a Generalised Linear Model and an ANOVA for (a) and a one-way ANOVA for (b) at p <0.05.


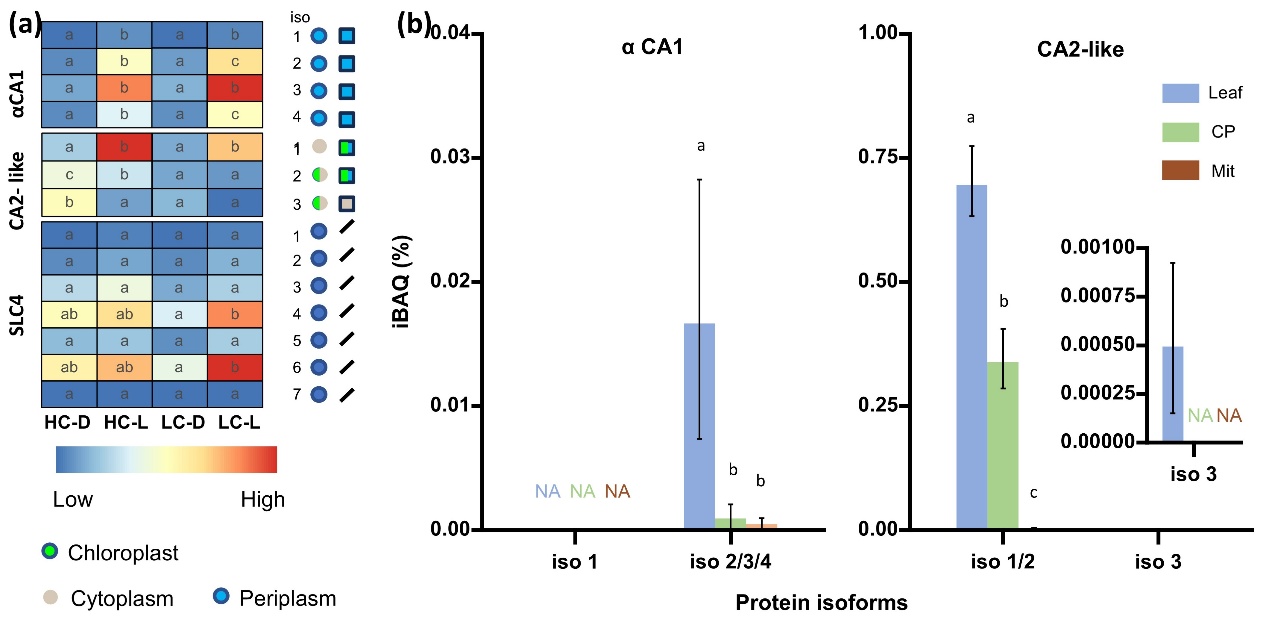


**Fig. S13. Distance between chloroplasts and mitochondria for *Ottelia alismoides*.** Plants were grown at high (HC) and low (LC) inorganic carbon. Median distances (μm) are shown and the p value calculated from a Wilcoxon test is the significance of the difference between the two treatments with n = 86 for HC and n = 103 for LC.


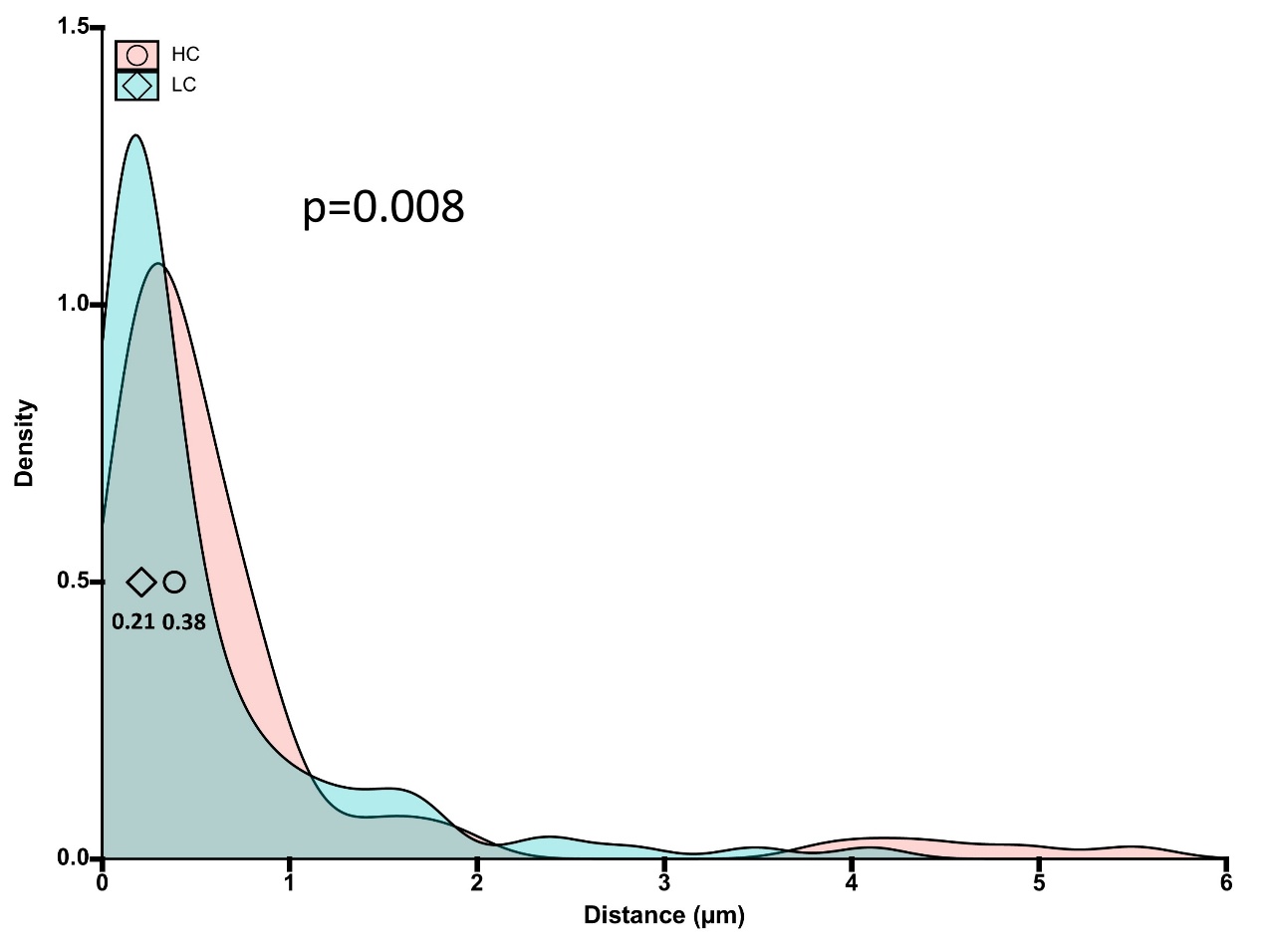


**Table S1** **Amino acid sequences of proteins derived from transcriptomic data used
in this paper on *Ottelia*** ***alismoides*. Abbreviations are: alanine aminotransferase (AlaAT), aspartate aminotransferase (AspAT), bile acid sodium symporter (BASS2), carbonic anhydrase (CA), dicarboxylate/tricarboxylate transporter (DTC), glyceraldehyde-3-phosphate dehydrogenase (GAPDH), glucose-6-phosphate translocator (GPT2), malate dehydrogenase (MDH), mitochondrial pyruvate carrier (MPC1), NAD(P) malic enzyme (NAD(P)-ME), PEP carboxylase (PEPC), phosphoglucomutase (PGM), starch phosphorylase (PHS), PEPC kinase (PPCK), phosphoenolpyruvate transporter (PPT), phosphoglycerate kinase (PGK), pyruvate phosphate dikinase (PPDK), phosphoribulokinase (PRK), ribulose bisphosphate carboxylase-oxygenase small subunit (rbcS), solute carrier family 4 (SLC4), tonoplast dicarboxylate transporter (TDT), vacuolar-ATPase (V-ATPase).**

>αCA1 iso1a

MGSSSFFALGMAFLVLSCSNAQSIVKFGYSGQIGPMRWGSLCPSYSSCSQGRHQSPIDIAKLEAEFDPSLHPLLRDYASSNATLIDNGFNIMVKYEGNVGCVTVEGKNYTLKQMHWHSPSEHTIDGERFPMELHLVHMCDDGNVTVVAILFKYGTPNPFIAKLMGKVEEMTKDVHAGDKGAHIPIGLLNTKAMRHSAHGYYRYVGSLTTPPCTENVVWNVVSKVRDISKEQAEALMAPLEGEYRHNARPTQPLNGRRVRYYAQGPLQMPNKTK

>αCA1 iso2

MVNTIMIIVLAPLMFFMASAYKDQDLARFGYMGSLGPSNWGSLRRDYYLCANGTRQSPINIDTRKVVYDPTLRSLDRDYHVTNGTLFDNGFNVELRYPQTVGTLLVEGRNYTLKQMHWHLNSEHTIDGERFPVELHLVHTFKEELFTVVSILFKYGKPDPLVAKLLPRVDELTRQVKSGNTDAVIPVGFFDTSSLKRHTNKYYRYIGSVSTPPCTENVIWNILGKVRTMSLAQAEKLRAPLPEGFHVNARPTQPLNGRVVRLYREK

>αCA1 iso3

MANTIMIIVLAPLMFLMASAYKDQDLARFGYMGSLGPSNWGSLRRDYYLCANGTRQSPINIDTKKAVYDPTLRSLDRDYHVTNGTLFDNGFNVELRYPQTVGTLLVEGRNYTLKQMHWHFNSEHTINGERFPVELHLVHTFKEELFTVVSILFKYGKPDPLVAKLLPRVDELTRQVKSGNTDAVIPVGFFDTSSLERHTNKYYRYIGSVSTPPCTENVIWNVLGKVRTMSLAQAEKLRAPLPEGFHVNARPTQPLNGRVVRLYREN

>αCA1 iso4

MVNTIMIIVLAPLMFFMASAYKDQDLARFGYMGSLGPSNWGSLRRDYYLCANGTRQSPINIDTRKVVYDPTLRSLDRDYHVTNGTLFDNGFNVELRYPQTVGTLLVEGRNYTLKQMHWHLNSEHTIDGERFPVELHLVHTFKEELFTVVSILFKYGKPDPLVAKLLPRVDELTRQVKSGNTDAVIPVGFFDTSSLKRHTNKYYRYIGSVSTPPCTENVIWNILGKVSKYSAQYT

>SLC4 iso1

MEETFVPFQGIKNDLEARLKCYKQDWTGGFKAGFRILAPTTYIFFASAIPVISFGEQLERNTDGVLTAVQTLASTALCGVIHSILGGQPLLILGVAEPTVLMYTFMFNFVKDRADLGRRLFLAWTGWVCVWTALLLFLLAILGACSIINRFTRIAGELFGLLIAMLFMQEAIKGVIDEFHVPQRENRKLPEFELPWRFANGMFALVLSFGLILTSLKSRRARSWRFGSGWMRSLIADYGVPLMVLIWTAVSYLPYGTIPQGIPRRLFSPNPWSPGAYENWTVVKEMLEVPVLYIFGAFIPATMIAVLYYFDHSVASQLAQQKEFNLRKPPSFHYDLLLLGFLTLLCGLIGIPPANGVIPQSPMHTKSLATLKHQLLRNRLVATARKSMNGNSNLGQLYENMQEAYHQMQTPLVYQKPSTISGLKELKETSIQLASTMDSIDAPLDETVFDIEKEIDDLLPVEVKEQRLSNLLQALMVGSCLAAMPFLRKIPTSVLWGYFAFMAIESLPGNQFWERILLLFTAPSRRYKVLEECHATFVETVPFRTIAAFTAFQTAYLLVCFGITWIPIAGVLFPLMIMLLVPARQYLLPKLFKGAHLTDLDAAEYEESLAIKYHPEAASFHEIDQGKLGGSFKEAGEAMDEMITRSRGEVRRFSSPRVNSSPASRAYELNGILSPRFLVFSPRISELRSSQSPAADVQFGSRTASERRASNLGDAARKQMKRCESH

>SLC4 iso2

IESLPGNQFWERILLLFTAPNRRYKILEKYHVTFVESVPFKAIASFTIFQTVYLLLCFGITWIPIAGVLFPLLIMLLVPVRQYVLPKFFKGAHLTDLDAAEYEEILALPADFNDHCHHDDAEIMDGIVTRSRGEVRHLHSPKITSSTPSRNNTPKATPSPLGPERALSPHLKEITTARSATFVRTIPEADEEGSWSFKMGLNPHGSRKN

>SLC4 iso3

IESLPGNQFWERILLLFTAPNRRYKILEKYHVTFVESVPFKAIASFTIFQTVYLLLCFGITWIPIAGVLFPLLIMLLVPVRQYVLPKFFKGAHLTDLDAAEYEEILALPADFNDHGHHDDGEIMDGIVTRSRGEVRHLHSPKITSSTPSRHNTPKATPSPLGPERALSPHVMEITRARSAKFVRTIPETDEEGSWSFKMGQNPHGSSKN

>SLC4 iso4

MLSFLSYFFDSNCFIAKNRILEKYHVTFVESVPFKAIASFTIFQTVYLLLCFGITWIPIAGVLFPLLIMLLVPVRQYVLPKFFKGAHLTDLDAAEYEEILALPADFNDHRHHDDGEIMDGIVTRSRGEVRHLHSPKITSSTPSRHNTPKATPSPLGPERDLSPHVKEITRARSATFVRMIPEADEEGSWSFKMGQNPHGSHKN

>SLC4 iso5

IESLPGNQFWERILLLFTAPNRRYKILEKYHVTFVESVPFKAIASFTIFQTVYLLLCFGITWIPIAGVLFPLLIMLLVPVRQYVLPKFFKGAHLTDLDAAEYEEILALPADFNVHGHHDDGEIMDGIVTRSRGEVRHLHSPKITSSTPSRHNTPKATPSPLGPERALSPHVVEITRARSAKFVRTIPEADEEGSWSFKMGQNPHGSSKN

>SLC4 iso6

IESLPGNQFWERILLLFTAPNRRYKILEKYHVTFVESVPFKAIASFTIFQTVYLLLCFGITWIPIAGVLFPLLIMLLVPVRQYVLPKFFKGAHLTDLDAAEYEEILALPADFNDHRHHDDGEIMDGIVTRSRGEVRHLHSPKITSSTPSRHNTPKATPSPLGPERDLSPHVKEITRARSATFVRMIPEADEEGSWSFKMGQNPHGSHKN

>SLC4 iso7

VKTAKEGIRNQATNSEIYGKIEEVFIEMDKCETPVSAASELKDLKDAVMKDDKDNGTAEGSLDLEKHIDAHLPVRVNEQRVSNLLQSLLVGACVAFMPIIKQIPSSVLWGYFAYMAIDSLPGNQFWERILLLFVTPRRRYKILEGDHASFVESVPFKYIAAFTLFQFAYFLMCYGVTWIPIAGIMFPMPFFLLICIRQHILPKLCHPQYLYELDAAEYEEVIGAPHLPASFAFMGCEGSAIDSEDNSGHDTDQFGAELLEEFTTHRGEIKLRTRSYKEERFSPIYPENVNHQ

>CA2-like iso1

MPRSDPVLLQRHLSSHNFLQTNEDLHIRDITEEGAMEAIDRLNAGFAHFKSEVYEKKSEEFSKLAETQTPKFMVFACSDSRVCPSHVLNFQPGEAFMVRNIASMVGPYDPTKYSGTGAAVEYAVLHLKVENIVVVGHSRCGGIKGLMSIKEGSPLGSEFIEEWVKICTPAKLKVESECADLPFSEQCTKCEKEAVNVSLQNLLSYPFVKAGLENKTLSVIGAYYDFVNGNFEAWPLEA

>CA2-like iso2

MEAIDRLNAGFAHFKSEVYEKEPELFSRLAETQTPKFMVFACSDSRVCPSHILNFQPGEAFMVRNIANMVGPYDPTKYSGVGAAVEYAVLHLKVENIVVVGHSRCGGIKGLMSIKEGSSHATDFIEEWVKICEPAKLKVESECADLPFSEQCTKCEKEAVNVSLQNLLSYPFVKAGVESKTISVIGAYYDFVNGNFEAWPLEA

>CA2-like iso3

MNQEGTMEAIDRLNAGFAHFKTEVYEKNPEEFSRLAETQTPKFMVFACSDSRVCPSHILNFQPGEAFMVRNIANMVGPYDPTKYSGVGAAVEYAVLHLKVENIVVVGHSRCGGIKGLMSIKEGSSHGTDFIEEWVKICEPAKLKVESECADLPFSEQCTKCEKEAVNVSLQNLLSYPFVKAGVESKTISVIGAYYDFVNGSFEAWPLEA

>rbcS iso1

MASSMMVSSAAAVSRTAPAQTTMVAPFSGLKSVATFPVTRKANADLTALPSNGGRVQCIQVWPPEGVKKFETLSYLPPLSNESLAKEIDYLLNNGWVPCLEFSRVGFVYREHDKSPGYYDGRYWTMWKLPMFGCTDSAQVLAELEEVKKAYPDHFVRIIGFNNQKQVQCVSFIAYKPQANY

>rbcS iso2

MDQLAVVGEPVLDLYRGGLNAIPNPSSSPLPREEGSSMASSMTLSAATASAAVPVAQSNMVAPFTGLKSMASFPNRKANADMSALPNNGGRVQCIQVWPPEGKKKFETLSYLPPLTTEQLIKEVEYLLRSGWIPCLEFSKEGFVYRENHSSPGYYDGRYWTMWKLPMFGCTDAAQVLAEVEEAKKAYPNYFVRIIGFDNKRQVQCISFIAYKPPGN

>rbcS iso3

MASSMMLSAATASAAVPVAQSNMVAPFTGLKSMASFPNRKANADMSALPNNGGRVQCIQVWPPEGKKKFETLSYLPPLTTEQLIKEVEYLLRSGWIPCVEFSKEGFVYRENHRSPGYYDGRYWTMWKLPLFGCTDATQVVAEIEEVKKAYPDYFVRIIGFDNKRQVQCISFIAYKPPGN

>rbcS iso4

MASSTMLSAATTTAAVSVAQSNMVAPFTGLKSMASFPNRKANADMSSLPSNGGRVQCIQVWPPEGKKKFETLSYLPPLTTEQLIKEVEYLLRSGWIPCVEFSQEGFVYRENHRSPGYYDGRYWTMWKLPMFGCTDATQVVAEVEEVKKAYPGYFVRIIGFDNKRQVQCISFIAYKPPGN

>rbcS iso5

MSALPSNGGRVQCIQVWPPEGKKKFETLSYLPPLTTEQLIKEVEYLLRSGWIPCVEFSKEGFVYRENHRSPGYYDGRYWTMWKLPMFGCTDATQVVAEVEEVKKAYPGYFVRIIGFDNKRQVQCISFIAYKPPGN

>rbcS iso6

MPPSRWPNQTWSPPFTGLKSMASFPNRKANADMSALPNNGGRVQCIQVWPPEGKKKFETLSYLPPLTTEQLIKEVEYLLRSGWIPCVEFSKEGFVYRENHRSPGYYDGRYWTMWKLPLFGCTDATQVVAEIEEVKKAYPDYFVRIIGFDNKRQVQCISFIAYKPPGN

>PRK iso1

MAISTAYTTQALNFASSISTPSKTHLAHGNRHVIVYTTYDRRTRRSSGVTCSAESKTVVIGLAADSGCGKSTFMRRLTSVFGGAASPPKGGNPDSNTLISDTTTVICLDDYHSLDRHGRKEKGVTALDPRANNFDLMYEQVKALKDGVAVEKPIYNHVTGLLDPPELIQPPKILVIEGLHPMFDPRVRDLLDFSIYLDISNEVKFAWKIQRDMAERGHSLESIKASIQARKPDFDAYIDPQKQYSDAVIEVLPTQLIPDDNEGKVLRVRLIMKEGVKYFSPVYLFDEGSTIDWIPCGRKLTCSYPGIKFAYAPDTYFSNEVSILEMDGQFDRLDELIYVESHLSNLSTKFYGEVTQQMLKHSEFPGSNNGTGFFQTIVGLKIRDLYEQIVAEKAKTTLEAAKI

>PRK iso2

MCGTRREMAISTAYTTQALNFASSISTPSKTHLAHGNRHVIVYTTYDRKTRRSSGVTCSAESKTVVIGLAADSGCGKSTFMRRLTSVFGGAASPPKGGNPDSNTLISDTTTVICLDDYHSLDRHGRKEKGVTALDPRANNFDLMYEQVKALKDGVAVEKPIYNHVTGLLDPPELIQPPKILVIEGLHPMFDPRVRDLLDFSIYLDISNEVKFAWKIQRDMAERGHSLESIKASIQARKPDFDAYIDPQKQYSDAVIEVLPTRLIPDDNEGKVLRVRLIMKEGVKYFSPVYLFDEGSTIDWIPCGRKLTCSYPGIKFAYAPDTYFSNEVSILEMDGQFDRLDELIYVESHLSNLSTKFYGEVTQQMLKHSEFPGSNNGTGFFQTIVGLKIRDLYEQIVAEKANTSLEAAKI

>PRK iso3

MAISTAYTTQALNFASSISTPSKTHLAHGNRHVIVYTTYDRKTRRSSGVTCSAESKTVVIGLAADSGCGKSTFMRRLTSVFGGAASPPKGGNPDSNTLISDTTTVICLDDYHSLDRHGRKEKGVTALDPRANNFDLMYEQVKALKDGVAVEKPIYNHVTGLLDPPELIQPPKILVIEGLHPMFDPRVRDLLDFSIYLDISNEVKFAWKIQRDMAERGHSLESIKASIQARKPDFDAYIDPQKQYSDAVIEVLPTRLIPDDNEGKVLRVRLIMKEGVKYFSPVYLFDEGSTIDWIPCGRKLTCSYPGIKFAYAPDTYFSNEVSILEMDGQFDRLDELIYVESHLSNLSTKFYGEVTQQMLKHSEFPGSNNGTGFFQTIVGLKIRDLYEQIIAEKANTSLEAAKI

>PRK iso4

MAISTAYTTQALNFASSISTPSKTHLAHGNRHVIVYTTYDRRMRRSSGVTCSAESKTVVIGLAADSGCGKSTFMRRLTSVFGGAASPPKGGNPDSNTLISDTTTVICLDDYHSLDRHGRKEKGVTALDPRANNFDLMYEQVKALKDGVAVEKPIYNHVTGLLDPPELIQPPKILVIEGLHPMFDPRVRDLLDFSIYLDISNEVKFAWKIQRDMAERGHSLESIKASIQARKPDFDAYIDPQKQYSDAVIEVLPTRLIPDDNEGKVLRVRLIMKEGVKYFSPVYLFDEGSTIDWIPCGRKLTCSYPGIKFAYAPDTYFSNEVSILEMDGQFDRLDELIYVESHLSNLSTKFYGEVTQQMLKHSEFPGSNNGTGFFQTIVGLKIRDLYEQIVAEKAKTSLEAAKI

>PEPC iso1

GSVIRKLLSIDWYREHVVKNHNAHQEVMVGYSDSGKDAGRFTAAWELYKAQEDVVAACNEYGIKVTLFHGRGGSIGRGGGPTHLAIQSQPPGSVMGSLRSTEQGEMVQAKFGLPQTAVRQLEIYTTAVLLATLRPPLPPRKEKWRHVMEEISKTGCECYRSTVYENPDFLGYFQEATPQSELGFLNIGSRPTRRKASTGIGHLRAIPWIFAWTQTRFVLPAWLGVGKGLKDACEKGYSKDLRAMYKEWPFFQSTIDLIEMVLGKADIPIAKHYDEVLVSEHRRELGAELRREFLTTEKYVLIVSGHEKLSENNRSLRRLIESRLPYLNPINMLQVEILRRLRQDPDNTKLRDALLISINGIAAGMRNTG

> PEPC iso2

MSAMAPNPSLEKMASIDAQLRLLAPSKVSEDDKLVEYDALLGDRFLDILQDLHGEDLRETVQRCYELSAEYEGKLDPQKLEELGNVLTSLDPGDSIVVSSSFSHMLNLANLAEEVQIAFRRRAKLKKGDFADENSAATESDIEETLKRLVVQLKKSPQEVFDALKSQTVDLVFTAHPTQSIRRSLLQKHARIRNCLAQLYAKDITPDDKQELDEALQREIQAAFRTDEIRRTPPTPQDEMRAGMSYFHETIWKGVPKFLRRVDTALKNIGIDERVPYNAPLIQFSSWMGGDRDGNPRVTPEVTRDVCLLARMMAANLYYSQIEDLMFELSMWRCSDELRGRAVELHQSSRSVAKHYIEFWKHIPPNEPYRVILGEVRDKLYNTRERARHLLSNGMSDIPEEATFTNVEQFLEPLELCYRSLCSCGDQPIADGSLLDFLRQVSTFGLSLVRLDIRQESDRHTDVIDAITNHLGIGSYREWSEEQRQEWLLSELRGKRPLFGPDLPKTEEIADVLDTLHVISELPSDNFGAYIISMATAPSDVLAVELLQRECHVKKPLRVVPLFEKLADLEAAPAAVARLFSVDWYRNRIDGKQEVMIGYSDSGKDAGRLSAAWQLYKAQEELIKVAKQYGVKLTMFHGRGGTVGRGGGPTHLAILSQPPETIHGSLRVTVQGEVIEQSFGEEHLCFRTLQRFTAATLEHGMRPPVSPKPEWRALMDAMAVVATEEYRSIVFKEPRFVEYFRLATPEMEYGRMNIGSRPSKRKPSGGIESLRAIPWIFAWTQTRFHLPVWLGFGAAFKHVMEKDIRNLLVLQEMYNDWPFFRVTIDLVEMVFAKGDPNIASLYDKLLVSSDLLPFGEHLRKKYIEAKNLLLQVAGHKDLLEGDPYLKQRLRIRDSYITALNVCQAYTLKRIRDPSFDVKLRPHLSKEIVDSSKSAAELVKLNPESEYAPGLEDTLILTMKGIAAGMQNTG

> PEPC iso3

MLAMAPNPNLEKMASIDAQLRLLAPSKLSEDDKLVEYDALLGDRFLDILQDLHGEDLRETVQRCYELSAEYEGKLDPQKLDELGNVLTSLDPGDSIVVSSSFSHMLNLANLAEEVQIAFRRRPKVKKGDFADENSATTESDIEETLKRLVVQLKKSPQEVFDALKSQTVDLVFTAHPTQSIRRSLLQKHARIRNCLAQLYAKDITPDDKQELDEALQREIQAAFRTDEIRRTPPTPQDEMRAGMSYFHETIWKGVPKFLRRVDTALKNIGIDERVPYNAPLIQFSSWMGGDRDGNPRVTPEVTRDVCLLARMMAANMYYSQIEDLMFELSMWRCSDELRGRALELQQSSRSVAKHYIEFWKQIPPNEPYRVLLGEVRDKLYNTRERARHLLSNGTSDIPEEATFTNVEQFLEPLELCYRSLCSCGDQPIADGSLLDFLRQVSTFGLSLVRLDIRQESDRHTDVIDAITNHLGIGSYREWSEEQRQEWLLSELRGKRPLLGPDLPKTEEIADVLDTLHVISELPSDNFGAYIISMATAPSDVLAVELLQRECRVKKPLRVVPLFEKLADLEAAPAAVARLFSVDWYRNRIDGKQEVMIGYSDSGKDAGRLSAAWQLYKAQEELIKVAQQYGVKLTMFHGRGGTVGRGGGPTHLAILSQPPDTIRGSLRVTVQGEVIEQSFGEEHLCFRTLQRFTAATLEHGMRPPISPKPEWRALMDAMAVVATEEYRSIVFKEPRFVEYFRLATPEMEYGRMNIGSRPSKRKPSGGIESLRAIPWIFAWTQTRFHLPVWLGFGAAFKHVMEKDIRNLLVLQEMYNEWPFFRVTIDLVEMVFAKGDPSIASLYDKLLVSSDLWSFGERLREKYIETKNLLLQVAGHKDLLEGDPYLRQRLRIRDSYITTLNVCQAYTLKRIRDPSFNVKLRPHLSKEISAAELVKLNPESEYAPGLEDTLILTMKGIAAGMQNTG

> PEPC iso4

MLPMAPNPNLEKMASIDAQLRLLAPSKVSEDDKLVEYDALLLDRFLDILQDLHGEDLRETVQRCYELSAEYEGKLDPQKLEELGNVLTSLDPGDSIVVSSSFSHMLNLANLAEEVQIAFRRRAKLKKGDFADENSATTESDIEETLKRLVVQLKKSPQEVFDALKSQTVDLVFTAHPTQSIRRSLLQKHARIRNCLAQLYAKDITPDDKQEIDEALQREIQAAFRTDEIRRTPPTPQDEMRAGMSYFHETIWKGVPKFLRRVDTALKNIGINERVPYNAPLIQFSSWMGGDRDGNPRVTPEVTRDVCLLARMMAANLYYSQIEDLMFELSMWRCSDELRGRAIELHRSSRSVAKHYIEFWKQIPPNEPYRVILGDVRDKLYNTRERARHLLSNGMSDIPEEATFTNVEQFLEPLELCYRSLCSCGDQPIADGSLLDFLRQVSTFGLSLVRLDIRQESDRHTDVIDAITNHLGIGSYREWSEEQRQEWLLSELRGKRPLFGPDLPKTEEIADVLDTLHVISELPSDNFGAYIISMATAPSDVLAVELLQRECRVKKPLRVVPLFEKLADLEAAPAAVARLFSVDWYRNRIDGKQEVMIGYSDSGKDAGRLSAAWQLFKAQEELIKVAKQYGVKLTMFHGRGGTVGRGGGPTHLAILSQPPDTIHGSLRVTVQGEVIEQSFGEEHLCFRTLQRFTAATLEHGMHPPVSPKPEWRALMDEMAVAATEEYRSIVFEEPRFVEYFRLATPEMEYGRMNIGSRPSKRKPSGGIESLRAIPWIFAWTQTRFHLPVWLGFGASFKHVMEMDIRNLHVLQEMYNEWPFFRVTIDLVEMVFAKGDPSIASLYDKLLVSSDLWPFGEHLREKYIEAKNLLLQVAGHKDLLEGDPYLKQRLRLRDSYITTLNVCQAYTLKRIRDPSFNVKVRPHLSKEIVVSSKSAAELVKLNPESEYAPGLEDTLILTMKGIAAGMQNTG

>PPCK iso1

MSKELNKDYLVGDEIGRGRFGVVSHCTSAATGESYALKSVDKSAIVDAIDRECLDREAKFTALVTGHPHVVSLHDVYEDDTSLHLVLDLCSGADLYDRITGRSGGSGAPMSEAEAAEVMRPLMEAIAHCHRCGVAHRDVKPDNVLFDRSGRLVLADLGSAGWFGDGRAMDGLFGTPYYVAPEIIAGRPYGEKVDVWSAGVILYIMLSGMPPFYGESALEIFQAVLRDSVRFPSSVFGDVSPAAKDLIRRMLTKDVQRRLSAEQVLRHRWMASGMESRAEA

>PPCK iso2

MSKELNKDYLVGEEIGRGRFGVVSHCTSAATGESYALKSVDKSAVVDAVDRECLDREAKFTALVTGHPHVVSLHDVYEDDASLHLVLDLCSGADLYDRITGRSGGSGAPMSEAEAAEVMRPLMEAIAHCHRCGVAHRDVKPDNVLFDGMGRLVLADLGSAGWFGDGRAMDGLFGTPYYVAPEIVAGRPYGEKVDVWSAGVILYIMLSGMPPFYGESAVEIFQAVLRDNVRFPSSVFGDVSPAAKDLIRRMLSRDVHRRLSAEQVLRHRWMATGMESRAEA

>PPCK iso3

MSKELNKDYLVGDEIGRGRFGVVSHCTSAATGECYALKSVDKSAIVDAVDRECLDREAKFTALVTGHPHVVSLHDVYEDDASLHLVLDLCSGPDLYDRITGRSGGSGAPMTEAEAAEIMRPLMEAIAHCHRCGVAHRDVKPDNVLFDGMGRLVLADLGSAGWFGDGRAMDGLYGTPYYVAPEIVAGRLYGEKVDVWSAGVILYIMLSGMPPFYGESAVEIFQAVLRDSVRFPSSVFGDVSPAAKDLIRRMLSKDVHRRLSAEQVLRHRWMATGMESMAEA

>PPCK iso4

MSKELNKDYLVGDEIGRGRFGVVSHCTSAATGECYALKSVDKSAIVDAVDRECLDREAKFTALVTGHPHVVSLHDVYEDDASLHLVLDLCSGPDLYDRITGRSGGSGAPMTEAEAAEIMRPLMEAIAHCHRCGVAHRDVKPDNVLFDGMGRLVLADLGSAGWFGDGRAMDGLYGTPYYVAPEIVAGRLYGEKVDVWSAGVILYIMLSGMPPFYGESAVEIFQAVLRDSVRFPSSVFGDVSPAAKDLIRRMLSKDVHRRLSAEQVLRKFSAPPSIYFLRIC

>MDH1 iso1

MAKDPVRVLVTGAAGQIGYALVPMIARGVMLGSDQPVILHMLDIPPAAEALNGVKMELVDAAFPLLKGVVATTDVVEACTGVNIAVMVGGFPRKEGMERKDVMSKNVSIYKSQASALEKHAAPNCKVLVVANPANTNALILKEFAPSIPEKNITCLTRLDHNRALGQISERLGVQVSDVKNVIIWGNHSSTQYPDVSHATVNTSGGEKSVKELVGDDEWLKSEFITTVQQRGAAIIKARKLSSALSAASSACDHIRDWVLGTPEGTWVSMGVYSDGSYSVPAGLIYSFPMTCKSGEWSIVQGLSIDEFSRKKLDATAAELSEEKSLAYSCLS

> MDH1 iso2

MAKDPVRVLVTGAAGQIGYALVPMIARGVMLGPDQPVILHMLDIPPAAEALNGVKMELVDAAFPLLKGVVATTDVVEACTGVTVAVMVGGFPRKEGMERKDVMSKNVSIYKSQASALEKHAAPNCKVLVVANPANTNALILKEFAPSIPEKNITCLTRLDHNRALGQISERLGVQVSDVKNVIIWGNHSSTQYPDVSHATVNTSGGEKSVKELVGDDEWLKSEFITTVQQRGAAIIKARKFSSALSAASSACDHIRDWALGTPEGTWVSMGVYSDGSYGVPAGLIYSFPVTCKSGEWSIVQGLSIDEFSRKKLDATAAELSEEKSLAYSCLS

> MDH1 iso3

MAKDPVRVLVTGAAGQIGYALVPMIARGVMLGPDQPVILHMLDIPPAAEALNGVKMELVDAAFPLLKGVVATTDAIEACTGVNVAVMVGGFPRKEGMERKDVMSKNVSIYKSQASALEKHAAPNCKVLVVANPANTNALILKEFAPSIPEKNITCLTRLDHNRALGQISERLGVQVSDVKNVIIWGNHSSTQYPDVSHATVNTSGGEKSVKELVGDDEWLKSEFITTVQQRGAAIIKARKLSSALSAASSACDHIRDWVLGTPEGTWVSMGVYSDGSYSVPAGLIYSFPVTCKSGEWSIVQGLSIDEFSRKKLDATAAELSEEKSLAYSCLS

> MDH1 iso4

MAKDPVRVLVTGAAGQIGYALVPMIARGVMLGPDQPVILHMLDIPPAAEALNGVKMELVDAAFPLLKGVVATTDVVEACTGVTVAVMVGGFPRKEGMERKDVMSKNVSIYKSQASALEKHAAPNCKVLVVANPANTNALILKEFAPSIPEKNITCLTRLDHNRALGQISERLGVQVSDVKNVIIWGNHSSTQYPDVSHATVNTSGGDKSVKELVGDDEWLKSEFITTVQQRGAAIIKARKFSSALSAASSACDHIRDWVLGTPEGTWVSMGVYSDGSYGVPAGLIYSFPVTCKSGEWFIVQGLSIDEFSRKKLDATAAELSEEKSLAYSCLS

>MDHmit iso1

MSSSLLRSAFRQSSAAVRRRGFASGAIPERKVVVLGAAGGIGQPLSLLMKLNPLVSDLALYDIAGTSGVAADVSHVNTRAQVKGYAAEEQLGEALKGADVVIIPAGVPRKPGMTRDDLFNINAGIVKSLCTAIAKHCPHAIVNMISNPVNSTVPIASEVFKKAGTYDEKKLFGVTTLDVVRAKTFYAGKANVSVADVNVPVVGGHAGITILPLFSQATPSTNALSDGDVKALTKRTQDGGTEVVEAKAGKGSATLSMAYAGALFADACLKGLNGVPDVVECTFVQSSITELPFFASKVRLGKNGVEEVLGLGSLSDFEKQGLESLKPELKASIEKGIKFAKEN

>MDHmit iso2

MRAYVIRSAVRALRNGTSAGAHLRRSFATDALPERKVTILGAAGGIGQPLALLMKLNPLVSSLSLYDIAGTPGVAADVSHINTRAQVKGYAAEEQLGQALEGSDIVIIPAGVPRKPGMTRDDLFNINAGIVKSLSAAIAKYCPNALVNMISNPVNSTVPIAAEVFKKAGTYDEKKLFGVTTLDVVRAKTFYAGKANIPVAGVNVPVVGGHAGITILPLFSQATPATNALSDEDIKSLTKRTQDGGTEVVEAKAGKGSATLSMAYAGAVFADACLKGLNGELNIVECSFVQSTITELPFFASKVKLGKNGVEEVLGLGPLSDFEKAGLESLKPELKASIEKGIKFANGS

>MDHmit iso3

MRAYVIRSAARALRSGTSAGPHLRRSFASDALPERKVTILGAAGGIGQPLALLMKLNPLVSSLSLYDIAGTPGVAADVSHINTRAQVKGYAAEEQLGQALEGSDIVIIPAGVPRKPGMTRDDLFNINAGIVKSLSTAIAKYCPNALVNMISNPVNSTVPIAAEVFKKAGTYDEKKLFGVTTLDVVRAKTFYAGKANIPVAGVNVPVVGGHAGITILPLFSQATPATNALSDEDIKALTKRTQDGGTEVVEAKAGKGSATLSMAYAGAVFADACLKGLNGELNIIECSFVQSTITELPFFASKVKLGKNGVEEVLGLGPLSDFEKEGLESLKPELKASIEKGIKFANGN

>MDHmit iso4

MRAYVIRSAARALRNATSAGVHLRRSFASDALPERKVTILGAAGGIGQPLALLMKLNPLVSSLSLYDIAGTPGVAADVSHINTRAQVKGYAAEEQLGQALEGSDIVIIPAGVPRKPGMTRDDLFNINAGIVKSLSAAIAKYCPNALVNMISNPVNSTVPIAAEVFKKAGTYDEKKLFGVTTLDVVRAKTFYAGKANIPVAGVNVPVVGGHAGITILPLFSQATPATNALSDEDIKALTKRTQDGGTEVVEAKAGKGSATLSMAYAGAVFADACLKGLNGELNIVECSFVQSTVTELPFFASKVKLGKNGVEEVLGLGPLSDFEKEGLEGLKPELKASIEKGIKFANGN

>NAD-ME iso1 MGRMWSLARSAWNLRLARRFSAAIPGPCIVYKRGTDILHDPWFNKDTGFPLTERDRLGLRGLLPPRIISFSQQYDRFMESYQSLEKNTRGEPADVVALAKWRILNRLHDRNETLYYRVLIDNIKDFAPVIYTPTVGLVCQNYGGLYRRPRGMYFSAKDKGEMMSMIYNWPAKEVDMIVLTDGSRILGLGDLGVQGIGIPIGKLDMYVAAAGINPQRILPIMLDVGTNNEKLLGDNLYLGLRQPRLEGDEYLSIVDEFMEAAFARWPKAVIQFEDFQMKWAFETLHRYRKRYCMFNDDIQGTAGVALAGLLGAVRTQGRPLEEFVNQKIVVVGAGSAGIGVLKTAKQALMKMMGNTEKSEAKNPFWLLDKNGLITKKREDLDPTAAPFARGCGPEEPEGLGEGASLLEVVEKVRPHVLLGLSGVGGIFNEQILKAMKKSDSPRPAIFAMSNPTMNAECTAEEAFNHAGKNIIFASGSPFQDVDLGNGTVGHVNQANNMYLFPGIGLGTLLSGARHISDGMLQAAAECLASYITDDEVQKGIIFPSISSIRQITAQVGAAVVHAAVDEGLAEGYQLVGYRDLKCMTKEEIVDYVSRNMWFPVYSPLVQEK

>NAD-ME iso2 MGRMWSLARSAWNLRHARRFSAAIPGPCIVHKRGTDILHDPWFNKDTGFPLTERDRLGLRGLLPPRIISFSQQYDRFMESYHSLEKNTRGEPADVVALAKWRILNRLHDRNETLYYRVLIDNIKDFAPIIYTPTVGLVCQNYGGLYRRPRGMYFSAKDKGEMMSMIYNWPAKEVDMIVLTDGSRILGLGDLGVQGIGIPIGKLDMYVAAAGINPQRILPIMLDVGTNNQKLLGDNLYLGLRQPRLEGDEYLSIVDEFMEAAFARWPKAVIQFEDFQMKWAFETLDRYRKRYCMFNDDIQGTAGVALAGLLGAVRTQGRPLEEFVNQKIVVVGAGSAGIGVLKTAKQALMKMMGNTENSEAKNPFWLLDKNGLITKKREDLDPTAAPFARGCGPEEPEGLGEGASLLEVVEKVRPHVLLGLSGVGGIFNEQILKAMKKSDSPRPAIFAMSNPTMNAECTAEDAFNYAGKNIIFASGSPFQDVDLGNGTVGHVNQANNMYLFPGIGLGTLLSGARHISDGMLQAAAECLASYITDDEVQKGIIFPSISSIRQITTQVGAAVVHAAVDEGLAEGYQLVGYRDLKCMTKEEIIDYVSRNMWFPVYSPLVQEK

>NAD-ME iso3 MAILASRQLRSALIRRLAAPLAGWGSRSFATSEGSRPTILDKRSIDILHDPWFNKGTAFSMTERNRLDLRGLLPPCVMTPRQQIQRFMADLKRLEVYARDGKMDTLALAKWRILNRLHDRNETMYYKFLIENIEEYAPIVYTPTVGLVCQNYSGLFRRPRGMYFSAEDRGEMMSMVYNWPAEQVDMIVVTDGSRILGLGDLGIQGIGICIGKLDLYVAAAGINPQRVLPVMIDVGTNNEKLLKDPLYLGLQEHRLDGEEYISIIDEFMEAVFTRWPNVIVQFEDFQSKWAFKLLQRYRNTYRMFNDDVQGTAGVAIAGLLGAVRAQGRPMIDFPKQKIVVAGAGSAGIGVLNAARKTMSRMLGHNEYAFESATSQFWVVDALGLLTDGRADIDQDALPFARKSKEAEHQGLREGASLVEVVKKVKPDVLLGLSAVGGLFSSEVLAALKESTSTRPAIFAMSNPTKNAECTPEQAFSIVGDHIVFASGSPFSDVDLGNGKLGHCNQGNNMYLFPGIGLGTLLSGARVISDGMLQAAAECLAAYMKEEEVLNGIIYPSISKIRDITKEVAAAVVREAVADDLAEGYRKMDARDLQRLSPDEIKEYVKYNMWNPIYPTLVYKD

>NAD-ME iso4

MAILASRQLRSSLIRRLAAPLAGWGSRSFATAECSRPTIVDKRSIDILHDPWFNKGTAFSMTERNRLDLRGLLPPNVMTPRQQIQRFMADLKRLEVYARDGKMDTLALAKWRILNRLHDRNETMYYKFLIENIEEYAPIVYTPTVGLVCQNYSGLFRRPRGMYFSAEDRGEMMSMVYNWPAEQVDMIVVTDGSRILGLGDLGIQGIGISIGKLDLYVAAAGINPQRVLPVMIDVGTNNEKLLKDPLYLGLQEHRLDGEEYISIIDEFMEAVFTRWPNVIVQFEDFQSKWAFKLLQRYRNTYRMFNDDVQGTAGVAIAGLLGAVRAQGRPMIDFPKQKIVVAGAGSAGIGVLNAARKTMSRMLGNNEYAFESATSQFWVVDALGLLTDGRAEINPDALPFARKSKEAEHQGLREGASLVEVVQKVKPDVLLGLSAVGGLFSTEVLAALKESTSTRPAIFAMSNPTNNAECTPEQAFSIVGDHIVFASGSPFSDVDLGNGKLGHCNQGNNMYLFPGIGLGTLLSGARVISDGMLQAAAECLAAYMKEEEVLNGIIYPSISMIRDITKEVAAAVVREAVAEDLAEGYRKMDARDLQRLSPDEIKAYVKYNMWNPIYPTLVYKD

> NADP-ME iso1

MKHRGRKAVAVCSGWNRRMGSCDNNEVVVLERENGSVDRAILDQPITPWTISVASGYTLLRDPRHNKGLAFTEKERDAHYLRGLLPPVCISQELQEKKLMHNLREYKIPLQRYMALMDLQERNQRLFYKLLIDNVEELLPVVYTPTVGEACEKYGSIFRRPQGLYISLKEKGKVLEVLKNWPERNIQVIVVTDGERILGLGDLGCQGMGIPVGKLALYTALGGIRPSSCLPVTIDVGTNNEKLLKDEFYIGLRRKRATGEEYAALLEEFMTAVKQNYGERVLVQFEDFANHNAFRLLSTYSKTHLVFNDDIQGTASVVLAGIIAALKLVGGSLAEHTFLFLGAGEAGTGIAELIALEVSKQTKAPVEESRKKIWLVDSKGLIIRSRMESLQHFKKPWAHKHEPIKGLLEAVNSIKPTMLIGSSGVGQTFTQEVVEAMASFNEKPIILALSNPTSQSECTAEQAYTWSKGRVIFASGSPFDPVEYEGKTFLSGQANNAYIFPGFGLGLVMSGAIRVHDDMLLAASHALAQQVTEENFEKGLIYPPFSNIRKISAQIAANVATKAYELGLASHLPRPQNLVKYAEKCMYSPVYRSYL

> NADP-ME iso2

MEGGSGAFSVANVGVGDAYGEDRATEEQLVTPWTVSVASGYTLLRDPRHNKGLAFTEKERDAHYLRGLLPPACITQELQEKKLMHSLRQYQVPLMRYMAMMDLQERNQRLFYKLLIDNVEELLPVVYTPTVGEACQKYGCIYRRPQGLYISLKEKGKVLEVLKNWPERNIQVIVVTDGERILGLGDLGCQGMGIPVGKLALYTALGGIRPSSCLPITIDVGTNNEQLLNDDFYIGLKQKRATGKDYEDLLHEFMTAVKQNYGERVLIQFEDFANHNAFELLSKYSETHLVFNDDIQGTASVVLAGVVAALKLVGGSLAEHTFLFLGAGEAGTGIAELIALELSKQTKVPIKETRKKIWLVDSKGLIVSSRKESLQHFKKPWAHDHEPVKYLVDAVKAVKPTVLIGASGVGRTFTKEVIEAMASINEKPIILALSNPTSQSECTAEEAYTWSQGRAIFASGSPFDPVEYEGKKFIPGQANNAYIFPGFGLGLVMSGAIRVHNDMLIAASEALANQVTEEDFEKGLIYPPFSNIRKISANIAANVAAKAYDLGLASHLPRPADLVKYAESCMYTPNYRSYR

> NADP-ME iso3

MLTFWIRETVAGSKRRSRSSCESAMEGSSGASAVANVGVGDAYGEDRATEEQLVTPWTVSVASGYTLLRDPRHNKGLAFTEKERDAHYLRGLLPPACITQELQEKKLMHSLRQYQVPLMRYMAMMDLQERNQRLFYKLLIDNVEELLPVVYTPTVGEACQKYGCIYRRPQGLYISLKEKGKVLEVLKNWPERNIQVIVVTDGERILGLGDLGCQGMGIPVGKLALYTALGGIRPSSCLPITIDVGTNNEQLLNDDFYIGLKQKRATGKDYEDLLHEFMTAVKQNYGERVLIQFEDFANHNAFELLSKYSETHLVFNDDIQGTASVVLAGVVAALKLVGGSLAQHTFLFLGAGEAGTGIAELIALELSKQTKVPIEGTRKKIWLVDSKGLIVSSRKESLQHFKKPWAHDHEPVKYLVDAVKAVKPTVLIGASGVGRTFTKEIIEAMTSINEKPIILALSNPTSQSECTAEEAYTWSQGRAIFASGSPFDPVEYEGRKFIPGQANNAYIFPGFGLGLVMSGAIRVHNDMLIAASEALANQVTEEDFEKGLIYPPFSNIRKISANIAANVAAKAYDLALASHLPRPADLVKYAESCMYSPNYRSYR

>PPDK iso1

MAGVITGLPSSSTTTATATASASILVKGGDEVSNGTSGCEIGFPGMSRCSSDPALQSLCGKPAAPIHAVVAQPPPTTKRVFNFGKGKSEGNKDMKNLLGGKGANLAEMASIGLSVPPGLTVSTEACDEYQKNDHKLPSGLWEEILEGLRAVEADMGCSLGDPSKPLLVSVRSGAAISMPGMMDTVLNLGLNDEVVEGLAGKSGERFAYDSYRRFLDMFGDVVLGISHSLFEEKLERLKAAKGVSLDTDLTAADLKELVGLYKQVYVDAKGEQFPSDPERQLYLAVLAVFDSWDSARAIKYRSINQITGLKGTAVNIQCMVFGNMGDTSGTGVLFTRNPSTGEKKLYGEFLINAQGEDVVAGIRTPEDLDSMKQHMPDAYRELVENCDILESHYKDMMDIEFTVQDNRLWMLQCRVGKRTGKGAVKIAVDMVAEGLIDTRAAIKRVEPGHLDQLLHPQFENPSAYKDQVLAVGLPASPGAALGQIVFNAEDAESWHAQGKSVILVRTETSPEDVGGMHAAAGILTARGGMTSHAAVVARGWGKCCVCGCSDIRVNDAEKVVVIGSNVLHEGDWISLNGSTGEVILGKQPLIPPALSGDLGTFMSWVDEVRQIKVMANADAPDDALTARNNGAQGIGLCRTEHMFFASDERIKAVRQMIMAVTLEQRKAALDLLLPYQRSDFEGIFRAMDGLPVTIRLLDPPLHEFLPEGEIADIVAKLTKDTDMTEEQIISRIEKLSEVNPMLGFRGCRLGISYPELTEMQARAIFEAAISMTNLGFKVFPEIMVPLVGTPEELKHQVGVIRKIANEVFSSMGTSIGYKVGTMIEIPRAALIADEIAEHAEFFSFGTNDLTQMTFGYSRDDVGKFLPIYLSQGLLQNDPFEVLDQKGVGQLVKIATKRGRAVRPDLKIGICGEHGGEASSVAFFVETGLDYVSCSPFRVPIARLAAAQAAL

>PPDK iso2

MKGVITGLPSSSSTATTTASARRLVKGGDEVSNGTSGCRIGRPGMSRCSSDPMLQSLSRKSAGPIRAVVAQPPATTKRVFNFGKGKSEGNKDMKTLLGGKGANLAEMASIGLSVPPGLTVSTEACDEYQKNGHKLPSGLWEEILEGLRAVEADMGFSLGDPSKPLLLSVRSGAAISMPGMMDTVLNLGLNDEVVEGLAAKSGERFAYDSYRRFLDMFGDVVMGISHSLFEEKLERLKAAKGVSLDTDLTAADLKELVGLYKQVYVDAKGEQFPSDPERQLYLAVLAVFDSWDSARAIKYRSINQITGLKGTAVNVQCMVFGNMGDTSGTGVLFTRNPSTGEKKLYGEFLINAQGEDVVAGIRTPEDLDSMKRHMPDAYRELVENCDILESHYKDMMDIEFTVQDNRLWMLQCRVGKRTGKGAVKIAVDMVTEGLIDARAAIKRVEPGHLDQLLHPQFENPSAYKDQVMAVGLPASPGAALGQIVFNAEDAESWHAQGKSVILVRTETSPEDVGGMHAAAGILTARGGMTSHAAVVARGWGKCCVCGCSDVRVNDAEKVVVIGSKVLQEGDWISLNGSTGEVILGKQPLSPPALSGDLGTFMSWVDEVRQIKVMANADAPDDALTARNNGAQGIGLCRTEHMFFASDERIKAVRQMIMAVTLEQRKAALDLLLPYQRSDFEGIFRAMDGLPVTIRLLDPPLHEFLPEGEIEDIVAKLTKDTDMTEEQIISRIEKLSEVNPMLGFRGCRLGISYPELTEMQARAIFEAAISMTNQGFKVFPEIMVPLVGTPEELKHQVGVIRKIAKEVFGSMGTSIGYKVGTMIEIPRAALIADEIAEHAEFFSFGTNDLTQMTFGYSRDDVGKFLPIYLSQGLLQNDPFEVLDQKGVGQLVKIATKRGRAVRPDLKVGICGEHGGEASSVAFFVEAGLDYVSCSPFRVPIARLAAAQAAL

>PPDK iso3

MLWIEVEREGEEYPRSTMKGVITGLPSSSSTATTTASARRLVKGGDEVSNGTSGCRIGRAGMSRCSSDPMLQSLCRKSAAPIRAVVAQPPPTTKRVFNFGKGKSEGNKDMKTLLGGKGANLAEMASIGLSVPPGLTVSTEACDEYQKNGHKLPGGLWEEILEGLRAVEADMGCSLGDPSKPLLLSVRSGAAISMPGMMDTVLNLGLNDEVVEGLAAKSGDRFAYDSYRRFLDMFGDVVMGISHSLFEEKLERLKAAKGVSLDTDLTAADLKELVGLYKQVYVDAKGEQFPSDPERQLYLAVLAVFDSWDSARAIKYRSINQITGLKGTAVNVQCMVFGNMGDTSGTGVLFTRNPSTGEKKLYGEFLINAQGEDVVAGIRTPEDLDSMKQHMPDAYRELVENCDILESHYKDMMDIEFTVQDNRLWMLQCRVGKRTGKGAVKIAVDMVTEGLIDTRAAIKRVEPGHLDQLLHPQFENPSAYKDQVLAVGLPASPGAALGQIVFNAEDAESWHAQGKSVILVRTETSPEDVGGMHAAAGILTARGGMTSHAAVVARGWGKCCVCGCSDVRVNDAEKVVVIGSKVLQEGDWISLNGSTGEVILGKQPLSPPALSGDLGTFMSWVDEVRQIKVMANADAPDDALTARNNGAQGIGLCRTEHMFFASDERIKAVRQMIMAVTLEQRKAALDLLLPYQRSDFEGIFRAMDGLPVTIRLLDPPLHEFLPEGEIEDIVAKLTKDTDMTEEQIISRIEKLSEVNPMLGFRGCRLGISYPELTEMQARAIFEAAISMTNQGFKVFPEIMVPLVGTPEELKHQVGVIRKIAKEVFGSMGTSIGYKVGTMIEIPRAALIADEIAEHAEFFSFGTNDLTQMTFGYSRDDVGKFLPIYLSQGLLQNDPFEVLDQKGVGQLVKIATKRGRAVRPDLKIGICGEHGGEASSVAFFVEAGLDYVSCSPFRVPIARLAAAQAAL

>PPDK iso4

MNGSTGEVILGKQPLSPPALSGDLGTFMSWVDEVRQIKVMANADAPDDALTARNNGAQGIGLCRTEHMFFASDERIKAVRQMIMAVTLEQRKAALDLLLPYQRSDFEGIFRAMDGLPVTIRLLDPPLHEFLPEGEIEDIVAKLTKDTDMTEEQIISRIEKLSEVNPMLGFRGCRLGISYPELTEMQARAIFEAGISMTNQGFKVFPEIMVPLVGTPEELKHQVGVIRKIAKEVFGSMGTSIGYKVGTMIEIPRAALIADEIAEHAEFFSFGTNDLTQMTFGYSRDDVGKFLPIYLSQGLLQNDPFEVLDQKGVGQLVKIATKRGRAVRPDLKIGICGEHGGEASSVAFFVEAGLDYVSCSPFRVPIARLAAAQAAL

>PPDK iso5

MKGVITGLPSSSCTTTATTTASTRLLVKGGDEVSNGTSGCKIGHPGMSRCSSDPMLQSLCRKPAAPIRAVVIAQPPATTKRVFNFGKGKSEGNKDMKTLLGGKGANLAEMASIGLSVPPGLTVSTEACDEYQKNGHKLPSGLWEEILEGLRAVEADMGCSLGDPSKPLLLSVRSGAAISMPGMMDTVLNLGLNDDVVEGLAAKSGERFAYDSYRRFLDMFGDVVMGISHFLFEEKLERLKAAKWVSLDTDLTAADLRELVGLYKQVYVDAKGEQFPSDPERQLYLAVLAVFDSWDSARAIKYRSINQITGLKGTAVNVQCMVFGNMGDTSGTGVLFTRNPSTGEKKLYGEFLINAQGEDVVAGIRTPEDLDSMKQHMPDAYRELVENCDILESHYKDMMDIEFTVQDNRLWMLQCRVGKRTGKGAVKIAVDMVAEGLIDTRAAIKRVEPGHLDQLLHPQFENPSAYKDQVLAVGLPASPGAALGQIVFNAEDAESWHAQGKSVILVRTETSPEDVGGMHAAAGILTARGGMTSHAAVVARGWGKCCVCGCSDIRVNDAEKVVVIGSKVLHEGDWISLNGSTGEVILGKQPLSPPALSGDLGTFMSWVDEVRQIKVMANADAPDDALTARNNGAQGIGLCRTEHMFFASDERIKAVRQMIMAVTLEQRKAALDLLLPYQRSDFEGIFRAMDGLPVTIRLLDPPLHEFLPEGEIEDIVAKLTKDTDMTEEQIISRIEKLSEVNPMLGFRGCRLGISYPELTEMQARAIFEAGISMTNQGFKVFPEIMVPLVGTPEELKHQVGVIRKIAKEVFSSMGTSIGYKVGTMIEIPRAALIADEIAEHAEFFSFGTNDLTQMTFGYSRDDVGKFLPIYLSQGLLQNDPFEVLDQKGVGQLVKIATKRGRAVRPDLKIGICGEHGGEASSVAFFVETGLDYVSCSPFRVPIARLAAAQAAL

>PPDK iso6

MGKQPLIPPALSGDLGTFMSWVDEVRQIKVMANADAPDDALTARNNGAQGIGLCRTEHMFFASDERIKAVRQMIMAVTLEQRKAALDLLLPYQRSDFEGIFRAMDGLPVTIRLLDPPLHEFLPEGEIEDIVAKLTKDTDMTEEQIISRIEKLSEVNPMLGFRGCRLGISYPELTEMQARAIFEAGISMTNQGFKVFPEIMVPLVGTPEELKHQVGVIRKIANEVFSSMGTSIGYKVGTMIEIPRAALIADEIAEHAEFFSFGTNDLTQMTFGYSRDDVGKFLPIYLSQGLLQNDPFEVLDQKGVGQLVKIATKRGRAVRPDLKIGICGEHGGEASSVAFFVETGLDYVSCSPFRVPIARLAAAQAAL

>PPDK iso7

MGDTSGTGVLFTRNPSTGEKKLYGEFLINAQGEDVVAGIRTPEDLDSMKQHMPDAYRELVENCDILESHYKDMMDIEFTVQDNRLWMLQCRVGKRTGKGAVKIAVDMVTEGLIDTRAAIKRVEPGHLDQLLHPQFENPSAYKDQVLAVGLPASPGAALGQIVFNAEDAESWHAQGKSVILVRTETSPEDVGGMHAAAGILTARGGMTSHAAVVARGWGKCCVCGCSDVRVNDAEKVVVIGSKVLQEGDWISLNGSTGEVILGKQPLSPPALSGDLGTFMSWVDEVRQIKVMANADAPDDALTARNNGAQGIGLCRTEHMFFASDERIKAVRQMIMAVTLEQRKAALDLLLPYQRSDFEGIFRAMDGLPVTIRLLDPPLHEFLPEGEIEDIVAKLTKDTDMTEEQIISRIEKLSEVNPMLGFRGCRLGISYPELTEMQARAIFEAGISMTNQGFKVFPEIMVPLVGTPEELKHQVGVIRKIAKEVFSSMGTSIGYKVGTMIEIPRAALIADEIAEHAEFFSFGTNDLTQMTFGYSRDDVGKFLPIYLSQGLLQNDPF

>PPT iso1

MPTFSSALSPSSAPFLARLSRRIIKPTPISARFFSNLHAPPSVNGLSALSPFPASLAHRRTSALVRATSAVPGESGGGRLDDGSVGEALATKAPGELYRVVELGILFGLWYLFNIYFNIYNKQILEVFPFPFTISASQFIFGSLFSIFMWLTGLYKRPKISMSMVLAILPLAAVHSVGNFCTNISLGKVSVSFTHTIKAMEPFFTVVLSAMFVGEMPSFLVLSSLVPIVGGVALASLTEASFNWYGFWSAMGSNLSNQYRNVLSKKLMVNNEESLDNITMFSIITIMSLILSAPVAYIVEGIKFTPTYLQSMGLNVQDIYVRALLAGISFHAYQQISYMILAKVSPVTHSVGNCVKRVVVIVSSVIFFRTPVSLVNSLGTGVALVGVFLYSRAKRIKEKAA

>PPT iso2

MQSSAFGFSAALAAPSSRLLRRPNPSTLSSAAPRVSLCLPVKPLRHLLAVPRSGSRWLSNPLLLTDREAGDVRASASAVPESAGAAEAKEGLWRTLQLGSLFGMWYLFNIYFNIYNKQVLKVFPFPITITTLQFSIGTLLVLFMWATNLHKRPKISGSQLVAILPLALVHTLGNLFTNMSLGKVAVSFTHTIKAMEPFFSVLLSAMFLGEMPSPLVVASLLPIVGGVALASFTESSFNWAGFWSAMASNVTFQSRNVLSKKFMVKKEESLDNINLFSIITILSLFLLAPVTLFVEGVKLAPSTMQAAGLNVQQVYTRALLASLCFHAYQQVSYMILARVSPVTHSVGNCVKRVVVIVTSVLFFRTPVSLINSVGTGIALLGVFLYSRVKKLKPKTA

>PPT iso3

MWATNLHKRPRISGSQLVAILPLALVHTLGNLFTNMSLGKVAVSFTHTIKAMEPFFSVLLSAMFLGEMPSPLVVASLLPIVGGVALASFTESSFNWAGFWSAMASNVTFQSRNVLSKKFMVKKEESLDNINLFSIITILSLFLLAPVTLFVEGVKLAPSTMQAAGLNVQQVYTRALLASLCFHAYQQVSYMILARVSPVTHSVGNCVKRVVVIVTSVLFFRTPVSLINSVGTGIALLGVFLYSRVKKLKPKTA

>PPT iso4

MQSSAFGISATLTAPSSRLLRRPNPSPSPSAAPRISLCLPAKPLRRLTAAPSSGSRWLSNPLLLADQAAGDVRARASAVPESADAAEAKDGLWRTLQLGALFGLWYLFNIYFNIYNKQVLKVFPFPITITTLQFFIGTLLVLFMWATNLHKRPRISGSQLVAILPLALVHTLGNLFTNMSLGKVAVSFTHTIKAMEPFFSVLLSAMFLGEMPSPLVVASLLPIVGGVALASFTESSFNWAGFWSAMASNVTFQSRNVLSKKFMVKKEESLDNINLFSIITILSLFLLAPVTLFVEGVKLAPSTMQAAGLNVQQVYTRALLASLCFHAYQQVSYMILAKVSPVTHSVGNCVKRVVVIVTSVLFFRTPVSLVNSVGTGVALLGVFLYSRVKKPKPKTA

>PPT iso5

MQSSAFGISATLTAPSSRLLRRPNPSPSPSAAPRISLCLPAKPLRRLTAAPSSGSRWLPNPLLLVDQSAGDVRARASAVPESADAAEAKDGLWRTLQLGSLFGMWYLFNIYFNIYNKQVLKVFPFPITITTLQFSIGTLLVLFMWATNLHKRPRISGSQLVAILPLALVHTLGNLFTNMSLGKVAVSFTHTIKAMEPFFSVLLSAIFLGELPSPLVVASLLPIVGGVALASFTESSFNWAGFWSAMASNVTFQSRNVLSKKLMVKKEESLDNINLFSIITILSLFLLAPVTLFVEGVKLAPSTMQAAGLNVQQVYTRALLASLCFHAYQQVSYMILAKVSPVTHSVGNCVKRVVVIVTSVLFFRTPVSLVNSVGTGVALLGVFLYSRVKKPKPKTA

>PPT iso6

MQSSAFGISATLAAPSSRLLRRQNPNPSPSAAPRISLCLPAKPLRRLTAAPSSGSRWLPNPLLLVDQSAGDVRARASAVPESADAAEAKDGLWRTLQLGSLFGMWYLFNIYFNIYNKQVLKVFPFPITITTLQFSIGTLLVLFMWATNLHKRPRISGSQLVAILPLALVHTLGNLFTNMSLGKVAVSFTHTIKAMEPFFSVLLSAIFLGELPSPLVVASLLPIVGGVALASFTESSFNWAGFWSAMASNVTFQSRNVLSKKLMVKKEESLDNINLFSIITILSLFLLAPVTLFVEGVKLAPSTMQAAGLNVQQVYTRALLAGLCFHAYQQVSYMILAKVSPVTHSVGNCVKRVVVIVTSVLFFRTPVSLVNSVGTGVALLGVFLYSRVKKPKPKTA

>TDT iso1

MESTREPLLPLHGTNFRLHPSLRSLLSKSTLQIALGPLSCILICFSGILDGHGASRNMLGITAWIFAWWLTGAVPAAITALVPLYLFPTLGIAPADVVAKSYMSDGIALLLGSFILVIAVEHYNIHQRLALNVALLFCGDPMSPRMLLMGISGTTAFISFWMQNTAAAVMMMPVATGIINRFPTGVEGNPDIDRYSKAVIMGVTYASEIGGVATLTGAAANMVAAGLWASHFPEERPIGFTSWSMFGLPLAVILFFSMWAILCLYYVPKSAGDVLSTHLDRSHLRRELDLLGIYLGVCVGRVPDPFYRDPPHAIVTGSRIDPTPSNGYPVQTDTRKFSMNHGYPRVPGKYCEHIDLDKIR

>TDT iso2

MESSREPLLPLHSTNFKLHPSLNSLLSKSTLHIALGPLSCILICLSGILDGHGASRNMLGITAWIFAWWLTGAVPVAITALAPLYLFPILGIAPADVVAKSYMSDVIALLLGSFILVIAVEHYNIHQRLALNVKPPKLSF

>TDT iso3

MESSREPLLPLHSTNFKLHPSLNSLLSKSTLHIALGPLSCILICLSGILDGHGASRNMLGITAWIFAWWLTGAVPVAITALAPLYLFPILGIAPADVVAKSYMSDVIALLLGSFILVIAVEHYNIHQRLALNVALLFCGDPMSPRMLLMGISGTTAFISFWMQNTAAAVMMMPVAMGIINRFPIGVEGNPDIDRYSKAVIMGVTYASEIGGIATLTGAAANMVAAALWASHFPEERPIGFTSWSMFGLPLAMILFFSMWAILCLYYVPKSAGDVLAAHLDRSHLRRELELLGIYVLISDLISLTNI

>V-ATPase iso1

MGFLLAANGLLVLYIAINLFKIYYGDDWEGLFEAITGYGLGGSSMALFGRVGGGIYTKAADVGADLVGKVERNIPEDDPRNPAVIADNVGDNVGDIAGMGSDLFGSYAESSCAALVVASISSFGINHDLTAMCYPLLISSMGIIVCLLTTLFATDLTEIKGVKQIEPALKWQLIISTILMTGGIAIVSWIALPSSFTIFNFGAQKVVTQWELFFCVAVGLWAGLVIGFVTEYFTSNAYSPVQDVADSCRTGAATNVIFGLALGYKSVIIPIFAIAVSIFVSFSLAAMYGIAVAALGMLSTIATGLAIDAYGPISDNAGGIAEMAGMSHRIRERTDALDAAGNTTAAIGKGFAIGSAALVSLALFGAFVSRAAISTVDVLTPKVFIGLIVGAMLPYWFSAMTMKSVGSAALKMVEEVRRQFNTIPGLMEGITKPDYATCVKISTDASIKEMIPPGALVMLTPLIVGILFGVETLSGVLAGALVSGVQIAISASNTGGAWDNAKKYIEAGASEHARSLGPKGSDPHKAAVIGDTIGDPLKDTSGPSLNILIKLMAVESLVFAPFFATHGGILFKIF

>V-ATPase iso2

MGAAILTDLLSEILIPVAAVIGIVFALLQWYLVSQVKLSAEYLIEEEEGLHDHSVVEKCAEIQSAIAEGATSFLFTEYKYVGLFMSAFAVLIFLFLGSVEGFSTENQPCTYSKDICKPALANAAFSTVSFILGAGTSLFSGFLGMKIATYANARTTLEARKGVGKAFITAFRSGAVMGFLLAANGLLVLYIAINLFKLYYGDDWEGLFEAITGYGLGGSSMALFGRVGGGIYTKAADVGADLVGKVERNIPEDDPRNPAVIADNVGDNVGDIAGMGSDLFGSYAESSCAALVVASISSFGINHDLTAMCYPLLISSMGIIVCLLTTLFATDLTEIKGVKQIEPALKWQLIISTILMTGGIAIVSWVALPSSFTIFNFGAQKVVTQWELFFCVAVGLWAGLVIGFVTEYFTSNAYSPVQDVADSCRTGAATNVIFGLALGYKSVIIPIFAIAISIFVSFSLAAMYGIAVAALGMLSTIATGLAIDAYGPISDNAGGIAEMAGMSNRIRQRTDALDAAGNTTAAIGKGFAIGSAALVSLALFGAFVSRAAISTVDVLTPKVFIGLIVGAMLPYWFSAMTMKSVGSAALKMVEEVRRQFNTIPGLMEGTTKPDYATCVKISTDASIKEMIPPGALVMLTPLIVGILFGVETLSGVLAGALVSGVQIAISASNTGGAWDNAKKYIEAGASEHAKSLGPKGSDPHKAAVIGDTIGDPLKDTSGPSLNILIKLMAVESLVFAPFFATHGGILFKIF

>V-ATPase iso3

MGAAILTDLLSEILIPVAAVIGIVFALLQWYLVSQVKLSAEGHGGSNNGAAEYLIEEEEGLHDHSVVEKCAEIQSAIAEGATSFLFTEYKYVGLFMSAFAVLIFLFLGSVEGFSTENQPCTYSKDICKPALANAAFSTVSFILGAGTSLFSGFLGMKIATYANARTTLEARKGVGKAFITAFRSGAVMGFLLAANGLLVLYIAINLFKLYYGDDWEGLFEAITGYGLGGSSMALFGRVGGGIYTKAADVGADLVGKVERNIPEDDPRNPAVIADNVGDNVGDIAGMGSDLFGSYAESSCAALVVASISSFGINHDLTAMCYPLLISSMGIIVCLLTTLFATDLTEIKGVKQIEPALKWQLIISTILMTGGIAIVSWVALPSSFTIFNFGAQKVVTQWELFFCVAVGLWAGLVIGFVTEYFTSNAYSPVQDVADSCRTGAATNVIFGLALGYKSVIIPIFAIAISIFVSFSLAAMYGIAVAALGMLSTIATGLAIDAYGPISDNAGGIAEMAGMSNRIRQRTDALDAAGNTTAAIGKGFAIGSAALVSLALFGAFVSRAAISTVDVLTPKVFIGLIVGAMLPYWFSAMTMKSVGSAALKMVEEVRRQFNTIPGLMEGTTKPDYATCVKISTDASIKEMIPPGALVMLTPLIVGILFGVETLSGVLAGALVSGVQIAISASNTGGAWDNAKKYIEAGASEHAKSLGPKGSDPHKAAVIGDTIGDPLKDTSGPSLNILIKLMAVESLVFAPFFATHGGILFKIF

>GPT2 iso1

MLCSAKHAAAPPLSVSSDLKRPRSPLTTTRPLSLPLSRSLGAAATAGGGNVSAEKPLYLDPRIRARRPVAKVRAYGADKAEGAAEGNAKAATAQRVKIGIYFATWWALNVVFNIYNKKVLNAFPYPWLTSTLSLATGSLMMLISWGIRIAETPKTDLNFWKALAPVAIAHTIGHVAATVSMSKVAVSFTHIIKSGEPAFSVIVSRLLLGETFPLPVYLSLIPIIGGCALAAVTELNFNMIGFMGAMISNVAFVFRNIFSKRGMDGLSVSGMNYYACLSMLSLVILTPFAVAVEGPQMWAAGWQKAVAEIGPHFVWWVAAQSVFYHLYNQVSYMSLDQISPLTFSIGNTMKRISVIVSSIIIFHTPVQPVNALGAAIAILGTFLYSQAKA

> GPT2 iso2

MATTALFSLKPAIPQPRIFPSLRPKSLPPLHLSFPSSPSLSLARPLHVSAAAPLRQCRLVARASEDAASVPIGAGHEEAPGAAAQKVKIGIYFATWWALNVVFNIYNKKVLNAFPFPWLTSTLSLATGSLIMLATWGTRIAEAPRTDLNFWKALAPVAVAHTIGHVAATVSMSKVAVSFTHIIKSGEPAFSVLVSRLLLGETFPVPVYLSLIPIIGGCALAAVTELNFNMTGTYFPRGG

> GPT2 iso3

MATTALFSLKPAIPQPRIFPSLRPKSLPPLHLSFPSSPSLSLARPLHVSAAAPLRQCRLVARASEDAASVPIGAGHEEAPGAAAQKVKIGIYFATWWALNVVFNIYNKKVLNAFPFPWLTSTLSLATGSLIMLATWGTRIAEAPRTDLNFWKALAPVAVAHTIGHVAATVSMSKVAVSFTHIIKSGEPAFSVLVSRLLLGETFPVPVYLSLIPIIGGCALAAVTELNFNMTGFMGAMISNVAFVFRNIFSKRGMTGKSVSGMNYYACLSMLSLLILTPFALAVEGPQMWAAGWQKAISEIGPHFVWWVAAQSVFYHLYNQVSYMSLDEISPLTFSIGNTMKRISVIVSSIIIFHTPVQPINALGAAIAILGTFLYSQAKA

>BASS2 iso1

MASISGLLLRPPAAAGWTIMSRRARPQRIEANPVFHRPITDPPPNLTSSRLLPRKPYLLRAAADPTDAGGGGITVGEAVTRAGEVLSMAFPVWISLACLAGLWRPSLFYWADRRWQIVGLTLTMLGMGMTLTLDDLKGALLMPKELLTGFVLQYTVMPLSGFFISQLLKLPSYYAAGLILVSCCPGGTASNIVTYLARGNVALSVLMTAASTFSAVVMTPLLTSKLAGQYVAVDPMGLFTSTVQVVLAPVLVGAVLNQYCNQLVKLVSPIMPLVAVACVAILCGSAISQNASAILSSGLQVVLASCALHASGFSLGYLLSRMLGVNVSSSRTISIEVGMQNSVLGVVLAGQHFSNPLTAVPCAVSSVCHSIYGSILAGIWRCMPPPAVQKKD

>BASS2 iso2

VNALRNDGQRKANVAVTCAKPFSVAPLSRKCQFVCKAEAEVSSNTPKEGSLYEKTVELLTTLFPVWVILGTIIGIYKPSAVTWLETDLFTLGLGFLMLSMGLTLTFEDFRRCLRNPWTVGVGFVAQYFIKPLLGFIIAMALKLPAPLATGLILVSCCPGGQASNVATYISRGNVALSVLMTTCSTIGAIIMTPLLTKLLAGQLVPVDAAGLAISTFQVVLVPTILGVLAHEYFPKFTEKIITVTPLIGVLLTTLLCASPIGQVSEVLKAQGAQLILPVAALHVAAFALGYWMSRFSSFGESTSRTVSIECGMQSSALGFLLAQKHFTNPLVAVPSAVSVVCMALGGSALAVFWRNRPIPSDDKDDFKE

>BASS2 iso3

MACSSSSMSRFLHGDAVLGGRSGFCRQRFGLSPARRGLQTTHLGIVNAPRNDGERKANVAITCAKPFSVAPLSRKCQFVCKAEVEVSSNTPEEGSLYVKTVELLTTLFPVWVILGTIIGIYKPSAVTWLETDLFTLGLGFLMLSMGLTLTFEDFRRCLRNPWTVGVGFVAQYFIKPLLGFIIAMALKLPAPLATGLILVSCCPGGQASNVATYISRGNVALSVLMTTCSTIGAIIMTPLLTKLLAGQLVPVDAAGLAISTFQVVLVPTILGVLAHEYFPKFTEKIITVTPLIGVLLTTLLCASPIGQVSEVLKAQGAQLILPVAALHVAAFALGYWMSRFSSFGESTSRTVSIECGMQSSALGFLLAQKHFTNPLVAVPSAVSVVCMALGGSALAVFWRNRPIPSDDKDDFKE

>BASS2 iso4

MSSSLASLSRFLLGDSMSQPSSTSFRKISPFASASMVRRRTHLAGDHELTVRRDGEGRQCPAIVWKTPFLPIVPITRKDSLLCKAEANISSVAPVEVSPYERIVELLTTLFPVWVILGTLVGIYKPSAVTWLETDLFTVGLGFLMLSMGLTLTFEDFRRCMRNPWTVGVGFLAQYLVKPLLGFVIAMALKLSAPLATGLILVSCCPGGQASNVATYISKGNVALSVLMTTCSTIGAIIMTPLLTKLLAGQLVPVDAAGLAISTFQVVLLPTIVGVLAHEYFPKFTEKLITVTPLIGVILTTLLCASPIGQVSEVLKSQGAQLILPVAALHIAAFALGYWFSRFSRFGESTSRTISIECGMQSSALGFLLAQKHFSNPLVAVPSAVSVVCMALGGSALAVFWRNVPIPFNDKDDFQE

>BASS2 iso5

MSSSSASISRFLIGDTKSQPCSTSFRKISSFASASMVRRRTHLAGDHELKVRRDGEGRQCPAIVCKTPFLSIVPIARKDSLSCKAEANISSVAPVEVSLYERIVELLTTLFPVWVILGTIVGIYKPSAVTWLETDLFTVGLGFLMLSMGLTLTFEDFRRCMRNPWTVGVGFLAQYLVKPLLGFVIAMALKLSAPLATGLILVSCCPGGQASNVATYISKGNVALSVLMTTCSTIGAIIMTPLLTKLLAGQLVPVDAAGLAISTFQVVLLPTIVGVLAHEYFPKFTEKLITVTPLIGVILTTLLCASPIGQVSEVLKSQGAQLILPVAALHIAAFALGYWFSRFSRFGESTSRTISIECGMQSSALGFLLAQKHFSNPLVAVPSAVSVVCMALGGSALAVFWRNVPIPVNDKDDFQE

>PHS iso1

MIINWNATYDYYNKVNPKQAYYLSMEFLQGRALLNAIGNLKLTGEYAAALEKLGHRLEDVAREEPDAALGNGGLGRLASCFLDSLATLDYPAWGYGLRYRYGLFQQLITKDGQEELAESWLEKGNPWEIVRHDVVYPVKFYGKVIVGSDGKKHWTGGENIQAVAYDVPIPGYNTRTTINLRLWSTKVPPRDFDLPAFNAGEHTKAVEGQINAEKICYILYPGDDSLEGKTLRLKQQYTLCSASLQDIIARFERRSGNVVNWEELPNKVAVQMNDTHPTLCIPELIRILVDEKGLSFQEAWKITQRTVAYTNHTVLPEALEKWSYDLMQKLLPRHVEIIELIDEELIQTIISQHGSADMDILQKKLKDMRVLENFEFPESVQKLFVKRKKVAPVKSKASLLVKDVEVSTKPSKDEEVEPEEEEIIEEEPTPKKSKLPKLVRMANLCVVGGHAVNGVAEIHSDIVKNDVFNEFYKLWPEKFQNKTNGVTPRRWIRFCNPELSKIITKWTGSEDWVVHTEKLAELRKYADNEDLQREWKDAKQANKMKVASFIKDRTGYDVSADAMFDIQVKRIHEYKRQLLNILGIVYRYKKMKEVSAEKRTSKFVPRVCIFGGKAFATYVQAKRIVKFITDVAATINHDPEIGDLLKVIFVPDYNVSVAELLIPASELSQHISTAGMEASGTSNMKFAMNGCVLIGTLDGANVEIRDEVGEDNFFLFGARAHEIAGLRKERVEGKFVPDPRFEEVKKFIRTGIFGGSSYNELLGSLEGNEGFGRGDYFLVGKDFPSYIECQEEVDKAYRDQKRWTKMSILNTAGSPKFSSDRTINDYAKDIWGIEPLPLP

>PGM iso1

MAIAASAAAIESSIALLSSQKLRSLALPSPSVAVGASTAGWRRVTPSLVVRRRGSVRISPSSAAVSSTVVEPQGPKVVSLPTKPYEGQKTGTSGLRKKVKVFQQENYLANWIQALFNSLPPEDYKDGLLVLGGDGRYFNREAAQIIIKIAAGNGVGKIIVGRDGILSTPAVSAIIRKWKANGGFIMSASHNPGGPEYDWGIKFNYNSGQPAPETITDKIYGNTLSISEIKLAETPDIDLSRLGTVEFGNFQVEVIDSVSDYLELMESVFDFDLIRNLLSRSDYRFVFDAMHAVTGAYAKPIFVDRLGADSGSILNGIPLEDFGHGHPDPNLTYAKELVDIMFAKNAPDFGAASDGDGDRNMILGKGFFITPSDSVAMIAANAQEAIPYFSSGPKGLARSMPTSGALDRVAAQLNLPFFEVPTGWKFFGNLMDVGKLSICGEESFGTGSDHIREKDGIWAVLAWLSIIAYRNKDKRIGEKLVSVADVAKEHWKKYGRNFFSRYDYEECESEGANKMIAHLRDLISKSKEGDTYGSYTLQFADDFTYTDPVDGSVASKQGIRLVFTDGSRIIYRLSGTGSAGATIRIYIEQYEPDASKHDADAQVALKPLIDLALSVSKLKEFTGREKPTVIT

>PGM iso2

MAIAASAAALESSIALLSSQKLRSLALPSPSVAVGASTAGGWRITPSLVARRRGSVRISPSSAAVSSTVVEPQGPKVASLPTKPYEGQKTGTSGLRKKVKVFQQENYLANWIQALFNSLPPEDYKDGLLVLGGDGRYFNREAAQIIIKIAAGNGVGKIIVGRDGILSTPAVSAIIRKWKANGGFIMSASHNPGGPEYDWGIKFNYNSGQPAPETITDKIYGNTLSISEIKLAETPDIDLSRLGTVEFGNFQVEVIDSVSDYLELMESVFDFDLIRNLLSRSDYRFVFDAMHAVTGAYAKPIFVDRLGADSGSILNGIPLEDFGHGHPDPNLTYAKELVDIMFAENAPDFGAASDGDGDRNMILGKGFFVTPSDSVAMIAANAQEAIPYFSSGPKGLARSMPTSGALDRVAAQLNLPFFEVPTGWKFFGNLMDVGKLSICGEESFGTGSDHIREKDGIWAVLAWLSIIAYRNKDKRIGEKLVSVADVAKEHWKKYGRNFFSRYDYEECESEGANKMIAHLRDLISKSKAGDTYGSYTLRFADDFTYTDPVDGSVASKQGIRLVFTDGSRIIYRLSGTGSAGATIRIYIEQYEPDASKHDANAQVALKPLIDLALSVSKLKEFTGREKPTVIT

>PGM iso3

MAIAASAAALESSIALLSSQKLRSLTLPSPSVAVGASTAGGWRATPYLVARRQGSVRVPPLSVAISATVVEPQGPKVVSVPTKPYEGQKTGTSGLRKKVKVFQQENYLANWIQALFSSLPPEDYKDGLLVLGGDGRYFNREATQIIIKIAAGNGVGKIIVGRDGILSTPAVSAIIRKWKANGGFIMSASHNPGGPEYDWGIKFNYNSGQPAPETITDKIYGNTLSISEIKLAETPDIDLSRLGTVEFGNFKVEVIDSVSDYLELMESVFDFDLIRNLLSRSDYRFVFDAMHAVTGAYAKPIFVDSLGADSGSILNGIPLEDFGHGHPDPNLTYAKELVDIMFAETAPDLGAASDGDGDRNMILGKGFFVTPSDSVAMIAANAQEAIPYFRSGPKGLARSMPTSGALDRVAAQLNLPFFEVPTGWKFFGNLMDDGKLSICGEESFGTGSDHIREKDGIWAVLAWLSIIAYRNKDKKIGEKPVSVADIAKEHRKKYGRNFFSRYDYEECESEGANKMITHLRDLISKSKAGDTYGSYTLRFADDFTYTDPVDGSIASKQGIRLVFTDGSRIIYRLSGTGSAGATIRIYIEQYEPDASKHDADAQVALKPLIDLALSISKLKEFTGREKPTVIT

>PGM iso4

MVLFTIARKETTPFLDQKPGTSGLRKKVTVFQQPNYLHNFVQSTFNALSAEKVKGATIVVSGDGRYFSKDAIQIIIKMAAANGVRRVWVGQNGLLSTPAVSAVIRERVGLDGSKSTGAFILTASHNPGGPHEDFGIKYNMENGGPAPEGITDKIYANTKTIKEYVIAEDLPDVDISSIGVTSFRGPGGDFDVDVFDSTTDYVKLMKSIFDFEAIKKLLASPKFSFCYDALHGVAGTYAKRIFVEELGADERSLLNCVPKEDFGGGHPDPNLTYAKELVARMGLGKSAPQEDPPEFGAAADGDADRNMVLGKRFFVTPSDSVAIIAANAVQSIPYFSSGLKGVARSMPTSAALDVVAKNLNLKFFEVPTGWKFFGNLMDAGVCSICGEESFGTGSDHIREKDGIWAVLAWLSILAHRNKDSLASEKLVSVEEIVLQHWASYGRHYYTRYDYENVDADGAKDLMANLVKLQSSLSDVNKIIKEIRPDVSDVVEADEFEYKDPVDGSVSKHQGIRYLFGDGSRLVFRLSGTGSVGATIRVYIEQYEKDPMKTGRDSQDALAPLVEVAIKLSKIQEFTGRSAPTVIT

>PGM iso5

MVLFTITRKETTPFLDQKPGTSGLRKKVTVFQQPNYLHNFVQSTFNALSAEKVKGATIVVSGDGRYFSKDAIQIIVKMAAANGVRCVWVGQNGLLSTPAVSAVIRERVGLDGSKSTGAFILTASHNPGGPHEDFGIKYNMENGGPAPEGITDKIYANTKTIKEYFIAEDLPDVDISSIGVTSFRGQGGDFDVDVFDSTTDYVKLMKSIFDFEAIKKLLASPKFSFCYDALHGVAGIYAKRIFVEELGADERSLLNCVPKEDFGGGHPDPNLTYAKELVARMGLGKSAPQEEPPEFGAAADGDADRNMVLGKRFFVTPSDSVAIIAANAVQSIPYFSSGLKGVARSMPTSAALDVVAKNLNLKFFEVPTGWKFFGNLMDAGVCSICGEESFGTGSDHIREKDGIWAVLAWLSILAHRNKDSLASEKLVSVEEIVLQHWASYGRHYYTRYDYENVDADGAKDLMANLVKLQSSLSDVNKIIKEIRPDVSDVVEADEFEYKDPVDGSVSKHQGIRYLFGDGSRLVFRLSGTGSVGATIRVYIEQYEKDPMKTGRDSQDALAPLVEVAIKLSKIQEFTGRSAPTVIT

>PGK iso1

MASAASSSALSLLPSSSRRRVAAAAATASLSLKTPIRRLGFAGAAADGSLALRVAESVRAAAGAARKGPRGVAAMAKRSVGDLTAADLKGKKVFVRADLNVPLDGDLNITDDTRIRAAIPTIQYLIKNGAKVILSSHLGRPKGVTPKYSLSPLVPRLSELLGITVQKADDCIGPDVEKLVAALPEGGVLLLENVRFYKEEEKNEPEFAQKLASVADLYVNDAFGTAHRAHASTEGVTKFLRPSVAGFLLQKELDYLVGAVSEPKRPFAAIVGGSKVSSKIGVIESLLEKVDILLLGGGMIFTFYKAQGLSVGSSLVEEDKLDLATSLLAKAKAKGVQLLLPTDVVIADKFAADANSQIVPASAIPDGWMGLDIGPDSIKTFNEALDTTKTVIWNGPMGVFEFEKFAVGTEAIANKLAELSSKDVVTIIGGGDSVAAVEKVGVAEKMSHISTGGGASLELLEGKELPGVIALDEALVAA

>PGK iso2

MRDHHINISSHQYYHHHSLCLSHPLTPSLSRMASAASSSALSLLPSSSRRRVAAAAATASLSLKTPVRRLGFVGAAADGSLALRVAESVRAAAGVARKGPRGVAAMAKRSVGDLTAADLKGKKVFVRADLNVPLDGDLNITDDTRIRAAIPTIQYLIKNGAKVILSSHLGRPKGVTPKYSLSPLVPRLSELLGITVQKADDCIGPDVENLVAALPEGGVLLLENVRFYKEEEKNEPEFAQKLASIADLYVNDAFGTAHRAHASTEGVTKFLRPSVAGFLLQKELDYLVGAVSEPKRPFAAIVGGSKVSSKIGVIESLLEKVDILLLGGGMIFTFYKAQGLSVGSSLVEEDKLDLATSLLAKAKAKGVQLLLPTDVVIADKFAADANSQIVPASAIPDGWMGLDIGPDSIKTFNEALDTTKTVIWNGPMGVFEFEKFAVGTEAIANKLAELSSKDVVTIIGGGDSVAAVEKVGVAEKMSHISTGGGASLELLEGKELPGVIALDEALVAA

>PGK iso3

MSLSSTHTPSLSRMASAASSTLSLLPSSSRRRVAAAAATASLSLKTPVRRLGFVGAAADGSLALRVAESVRAAAGVARKGPRGVAAMAKRSVGDLTAADLKGKKVFVRADLNVPLDGDLNITDDTRIRAAIPTIQYLIKNGAKVILSSHLGRPKGVTPKYSLSPLVPRLSELLGITVQKADDCIGPDVENLVAALPEGGVLLLENVRFYKEEEKNEPEFAQKLASIADLYVNDAFGTAHRAHASTEGVTKFLRPSVAGFLLQKELDYLVGAVSEPKRPFAAIVGGSKVSSKIGVIESLLEKVDILLLGGGMIFTFYKAQGLSVGSSLVEEDKLDLATSLLAKAKAKGVQLLLPTDVVIADKFAADANSQIVPASAIPDGWMGLDIGPDSIKTFNEALDTTKTVIWNGPMGVFEFEKFAVGTEAIANKLAELSSKDVVTIIGGGDSVAAVEKVGVAEKMSHISTGGGASLELLEGKELPGVIALDEALVAA

>PGK iso4

MDLFAHPRGIPAIGAEMATKKSVGDLKEVDLKGKKVFVRVDLNVPLDENQKITDDTRVRAAVPTIKYLRDNGAKVILCSHLGRPKGVTPKYSLKPLVPRLSELLGINVEIANDCIGEEVQKLVSALPDGGVLLLENVRFYKEEEKNVPEFAQKLASLAEVYVNDAFGTAHRAHASTEGVTKFLKPSVAGFLMQKELDYLVGAVANPKRPFAAIVGGSKVSTKIGVIESLFEKVNILLLGGGMIFTFYKAQGYPVGSSLVEEDKLDLAKGLIEKAKAKGVSLLLPTDVVIADKFDANANSEIVPASGIPDGWMGLDIGPDSIRTFSETLDSAKTIIWNGPMGVFEFEKFAAGTDAIAKKLAELTDKGATTIIGGGDSVAAVEKAGLASKMSHISTGGGASLELLEGKPLPGVLALDDA

>PGK iso5

MDENQKITDDTRVRAAVPTIKYLRDNGAKVILCSHLGRPKGVTPKYSLKPLVPRLSELLGINVEIANDSIGEEVQKLVSALPDGGVLLLENVRFYKEEEKNVPEFAQKLASLAEVYVNDAFGTAHRAHASTEGVTKFLKPSVAGFLMQKELDYLVGAVANPQRPFAAIVGGSKVSTKIGVIESLFEKVNILLLGGGMIFTFYKAQGYPVGSSLVEEDKLDLAKGLIEKAKAKGVSLLLPTDVVIADKFDANANSETVPASGIPDGWMGLDIGPDSIRTFSETLDTAKTIIWNGPMGVFEFEKFAAGTDAIAKKLAELTDKGATTIIGGGDSVAAVEKAGLASKMSHISTGGGASLELLEGKPLPGVLALDDA

>GAPDHC iso1

MSHTPHLLSSSSPPPPNLLSVSRALISTSMASKIKIGINGFGRIGRLVARVALLSDDVELVAVNDPFITTDYMVYMFKYDTVHGHWKHHDIKVKDSKTLLFGEKAVTVFGIRNPEEIPWGEAGADYVIESTGVFTDKDKAAAHLKGGAKKVVISAPSKDAPMFVVGVNEDKYTSDINILSNASCTTNCLAPIAKVLNDKFGILEGLMTTVHAVTATQKTVDGPSSKDWRGGRAAGFNIIPSSTGAAKAVGKVLPALNGKLTGMAFRVPTVDVSVVDLTVRLEKGASYDEIKAAVKEASETSLKGILGYVDEDLVSTDFIGDSRSSIFDAKAGIALNDKFVKVVAWYDNEWGYSNRVIDLVRHVSKTQ

> GAPDHC iso2

MLSSSSPPPPNLLSVSRALISTSMASKIKIGINGFGRIGRLVARVALLSDDVELVAVNDPFITTDYVIESTGVFTDKDKAAAHLKGGAKKVVISAPSKDAPMFVVGVNEDKYTSDINILSNASCTTNCLAPIAKVLNDKFGILEGLMTTVHAVTATQKTVDGPSSKDWRGGRAAGFNIIPSSTGAAKAVGKVLPALNGKLTGMAFRVPTVDVSVVDLTVRLEKGASYDEIKAAVKEASETSLKGILGYVDEDLVSTDFIGDSRSSIFDAKAGIALNDKFVKVVAWYDNEWGYSNRVIDLVRHVSKTQ

>GAPDHC iso3

MLSSSSPPPPNLLSVSRALISTSMASKIKIGINGFGRIGRLVARVALLSDDVELVAVNDPFITTDYMVYMFKYDTVHGHWKHHDIKVKDSKTLLFGEKEVTVFGIRNPEEIPWGEAGADYVIESTGVFTDKDKAAAHLKGGAKKVVISAPSKDAPMFVVGVNEDKYTSDINILSNASCTTNCLAPIAKVLNDKFGILEGLMTTVHAVTATQKTVDGPSSKDWRGGRAAGFNIIPSSTGAAKAVGKVLPALNGKLTGMAFRVPTVDVSVVDLTVRLEKGASYDEIKAAVKEASETSLKGILGYVDEDLVSTDFIGDSRSSIFDAKAGIALNDKFVKVVAWYDNEWGYSNRVIDLVRHVSKTQ

>GAPDHC iso4

MLSVSRALISTSMAGKIKIGINGFGRIGRLVARVALLSDDVELVAVNDPFITTDYMVYMFKYDTVHGHWKHHDIKVKDSKTLLFGEKEVTVFGIRNPEEIPWGEAGADYVIESTGVFTDKDKAAAHLKGGAKKVVISAPSKDAPMFVIGVNEDKYTSDINILSNASCTTNCLAPIAKVLNDKFGILEGLMTTVHAVTATQKTVDGPSSKDWRGGRAAGFNIIPSSTGAAKAVGKVLPALNGKLTGMAFRVPTVDVSVVDLTVRLEKGASYDEIKAAVKEASETSLKGILGYVDEDLVSTDFIGDSRSSIFDAKAGIALNDKFVKVVAWYDNEWGYSNRVIDLVRHVSKTQ

>GAPDHC iso5

MLFSSTPPPPNLFSFARSLISSSMASKIKIGINGFGRIGRLVARVALLSDDIDLVAVNDPFITTDYMVYMFKYDTVHGHWKHHDIKVKDSKTLLFGEKEVTVFGIRNPEEIPWGEAGADFVIESTGVFTDKDKAAAHLKGGAKKVVISAPSKDAPMFVVGVNEDKYTSDINILSNASCTTNCLAPIAKVLNDKFGILEGLMTTVHAVTATQKTVDGPSSKDWRGGRAAGFNIIPSSTGAAKAVGKVLPALNGKLTGMAFRVPTVDVSVVDLTVRLEKGASYDEIKAAVKEASETSLKGILGYVDEDLVSTDFIGDSRSSIFDAKAGIALNDKFVKVVAWYDNEWGYSNRVIDLVRHVSKTQ

>GAPDHC iso6

MLSFSRSLISTSMASKIKIGINGFGRIGRLVARVALLSDDIDLVAVNDPFITTDYMVYMFKYDTVHGHWKHHDIKVKDSKTLLFGEKEVTVFGIRNPEEIPWGEAGADFVIESTGVFTDKDKAAAHLKGGAKKVVISAPSKDAPMFVVGVNEDKYTSDINILSNASCTTNCLAPIAKVLNDKFGILEGLMTTVHAVTATQKTVDGPSSKDWRGGRAAGFNIIPSSTGAAKAVGKVLPALNGKLTGMAFRVPTVDVSVVDLTVRLEKGASYDEIKAAVKEASETSLKGILGYVDEDLVSTDFIGDSRSSIFDAKAGIALNDKFVKVVAWYDNEWGYSNRVIDLVRHVSKTQ

>GAPDHC iso7

MASSSAFLGSSANLSLDAPRDRLPSSASANVDPKGCHRMVSGGSRRRTSIFFKCAATRIEPLKATATEVPPELWSSPIGEKTRVGINGFGRIGRLVLRVATSRDDVEVVAVNDPFIDAKYMAYMLKYDSTHGVFNGIIHVVDDSTLEINGKRIAVLCKRDPADIPWGNLGAEYVVESSGVFTTLGKASAHIKGGAKKVVISAPSADAPMFVVGVNEKTYKSDMDIVSNASCTTNCLAPLAKVVHEEFGIVEGLMTTVHATTATQKTVDGPSMKDWRGGRGAGQNIIPSSTGAAKAVGKVLPALNGKLTGMAFRVPTPNVSVVDLTCRLQKSASYEDVKATIKYAADGPLKGILGYTDEDVVSNDFIGDSRSSIFDAKAGIGLSSSFMKLVAWYDNEWGYSNRVLDLIEHMALVNSHK

>GAPDHC iso8

MELSIGEKTRVGINGFGRIGRLVLRVATSRDDVEVVAVNDPFIDAKYMAYMLKYDSTHGVFNGIIHVVDDSTLEINGKRIVVLCKRDPADIPWGNLGAEYVVESSGVFTTLGKASAHIKGGAKKVVISAPSADAPMFVVGVNEKTYKSDMDIVSNASCTTNCLAPLAKVVHEEFGIVEGLMTTVHATTATQKTVDGPSMKDWRGGRGAGQNIIPSSTGAAKAVGKVLPALNGKLTGMAFRVPTPNVSVVDLTCRLQKSATYEDVKATIKYAADGPLKGILGYTDEDVVSNDFIGDSRSSIFDAKAGIGLSSSFMKLVAWYDNEWGYSNRVLDLIEHMALVNAHK

>GAPDHC iso9

MFGEKEVTVFGIRNPEEIPWGEAGADFVIESTGVFTDKDKAAAHLKGGAKKVVISAPSKDAPMFVVGVNEDKYTSDINILSNASCTTNCLAPIAKVLNDKFGILEGLMTTVHAVTATQKTVDGPSSKDWRGGRAAGFNIIPSSTGAAKAVGKVLPALNGKLTGMAFRVPTVDVSVVDLTVRLEKGASYDEIKAAVKEASETSLKGILGYVDEDLVSTDFIGDSRSSIFDAKAGIALNDKFVKVVAWYDNEWGYSNRVIDLVRHVSKTQ

>GAPDHC iso10

MLSFSRSLISTSMASKIKIGINGFGRIGRLVARVALLSDDIDLVAVNDPFITTDYMVYMFVVGVNEDKYTSDINILSNASCTTNCLAPIAKVLNDKFGILEGLMTTVHAVTATQKTVDGPSSKDWRGGRAAGFNIIPSSTGAAKAVGKVLPALNGKLTGMAFRVPTVDVSVVDLTVRLEKGASYDEIKAAVKEASETSLKGILGYVDEDLVSTDFIGDSRSSIFDAKAGIALNDKFVKVVAWYDNEWGYSNRVIDLVRHVSKTQ

>GAPDHC iso11

MLSFSRSLISTSMASKIKIGINGFGRIGRLVARVALLSDDIDLVAVNDPFITTDYMVYMFKYDTVHGHWKHHDIKVKDSKTLLFGEKEVTVFGIRNPEEIPWGEAGADFVIESTGVFTDKDKAAAHLKGGAKKVVISAPSKDAPMFVVGVNEDKYTSDINIIPSSTGAAKAVGKVLPALNGKLTGMAFRVPTVDVSVVDLTVRLEKGASYDEIKAAVKEASETSLKGILGYVDEDLVSTDFIGDSRSSIFDAKAGIALNDKFVKVVAWYDNEWGYSNRVIDLVRHVSKTQ

>GAPDHA iso1

MHLQAMASSTLLSSSMASASLQAGSKGFSEFSGLRNSSALPVARKVQADDFVSAIAFQTAAVRGSGYRKGVTEAKLKVAINGFGRIGRNFLRCWHGRKDSPLDVIAINDTGGVKQASHLLKYDSTLGIFDADVKPVGDTVISVDGKEIQVVSNRNPANLPWKELGIDLVIEGTGVFVDREGAGKHIQAGAKKVLITAPGKGDIPTYVVGVNADAYNPDEPIISNASCTTNCLAPFVKVLDQKFGIIKGTMTTTHSYTGDQRLLDASHRDLRRARAAALNIVPTSTGAAKAVALVLPSLKGKLNGIALRVPTPNVSVVDLVVQVEKKTFAEEVNAAFRESAEKELNGILSVCDEPLVSVDFRCTDVSSTVDASLTMVMGDDMVKVIAWYDNEWGYSQRVVDLADIVANQWK

>GAPDHA iso2

MHPQAMASSTFLSSSMASASLQAGSKGFSEFSGLRSSSALPVARKVQADDFVSAIAFQTAAVRGSGYRKGVTEAKLKVAINGFGRIGRNFLRCWHGRKDSPLDVIAINDTGGVKQASHLLKYDSTLGIFDADVKAVGDTVISVDGKEIQVVSNRNPANLPWKELGIDLVIEGTGVFVDREGAGKHIQAGAKKVLITAPGKGDIPTYVVGVNADAYSPDEPIISNASCTTNCLAPFVKVLDQKFGIIKGTMTTTHSYTGDQRLLDASHRDLRRARAAALNIVPTSTGAAKAVALVLPSLKGKLNGIALRVPTPNVSVVDLVVQVEKKTFAEEVNAAFRESAEKELNGILSVCDEPLVSVDFRCTDVSSTVDASLTMVMGDDMVKVIAWYDNEWGYSQRVVDLADIVANQWK

>GAPDHA iso3

MHPQAMASSTLLSSSMASASLQAGSKGFSEFSGLRSSSALPVARKVQADDFVSAIAFQTAAVRGSGYRKGVTEAKLKVAINGFGRIGRNFLRCWHGRKDSPLDVIAINDTGGVKQASHLLKYDSTLGIFDADVKAVGDTVISVDGKEIQVVSNRNPANLPWKELGIDLVIEGTGVFVDREGAGKHIQAGAKKVLITAPGKGDIPTYVVGVNADAYSPDEPIISNASCTTNCLAPFVKVLDQKFGIIKGTMTTTHSYTGDQRLLDASHRDLRRARAAALNIVPTSTGAAKAVALVLPSLKGKLNGIALRVPTPNVSVVDLVVQVEKKTFAEEVNAAFRESAEKELNGILSVCDEPLVSVDFRCTDVSSTVDASLTMVMGDDMVKVIAWYDNEWGYSQRVVDLADIVANQWK

>GAPDHA iso4

MASSTFLSSSMASASLQAGSKGFSEFSGLRSSSALPVARKVQADDFVSAIAFQTAAVRGSGYRKGVTEAKLKVAINGFGRIGRNFLRCWHGRKDSPLDVIAINDTGGVKQASHLLKYDSTLGIFDADVKAVGDTVISVDGKEIQVVSNRNPANLPWKELGIDLVIEGTGVFVDREGAGKHIQAGAKKVLITAPGKGDIPTYVVGVNADAYSPDEPIISNASCTTNCLAPFVKVLDQKFGIIKGTMTTTHSYTGDQRLLDASHRDLRRARAAALNIVPTSTGAAKAVALVLPSLKGKLNGIALRVPTPNVSVVDLVVQVEKKTFAEEVNAAFRESAEKELNGILSVCDEPLVSVDFRCSDVSSTVDASLTMVMGDDMVKVIAWYDNEWGYSQRVVDLADIVANQWK

>GAPDHA iso5

MRSSSALPVARKVQADDFVSAIAFQTAAVRGSGYRKGVTEAKLKVAINGFGRIGRNFLRCWHGRKDSPLDVIAINDTGGVKQASHLLKYDSTLGIFDADVKAVGDTVISVDGKEIQVVSNRNPANLPWKELGIDLVIEGTGVFVDREGAGKHIQAGAKKVLITAPGKGDIPTYVVGVNADAYSPDEPIISNASCTTNCLAPFVKVLDQKFGIIKGTMTTTHSYTGDQRLLDASHRDLRRARAAALNIVPTSTGAAKAVALVLPSLKGKLNGIALRVPTPNVSVVDLVVQVEKKTFAEEVNAAFRESADKELNGILSVCDEPLVSVDFRCSDVSSTVDASLTMVMGDDMVKVIAWYDNEWGYSQRVVDLADIVANQWK

>GAPDHA iso6

MASSTLLSSSMASASLQAGSKGFSEFSGLRNSSALPVARKVQADDFVSAIAFQTAAVRGSGYRKGVTEAKLKVAINGFGRIGRNFLRCWHGRKDSPLDVIAINDTGGVKQASHLLKYDSTLGIFDADVKAVGDTVISVDGKEIQVVSNRNPANLPWKELGIDLVIEGTGVFVDREGAGKHIQAGAKKVLITAPGKGDIPTYVVGVNADAYSPDEPIISNASCTTNCLAPFVKVLDQKFGIIKGTMTTTHSYTGDQRLLDASHRDLRRARAAALNIVPTSTGAAKAVALVLPSLKGKLNGIALRVPTPNVSVVDLVVQVEKKTFAEEVNAAFRESADKELNGILSVCDEPLVSVDFRCSDVSSTVDASLTMVMGDDMVKVIAWYDNEWGYSQRVVDLADIVANQWK

>GAPDHA iso7

MHPQAMASSTLLSSSMASASLQAGSKGFSEFSGLRSSSALPIARKVHADDFVSAIAFQTAAVRGSGYRKGVTEAKLKVAINGFGRIGRNFLRCWHGRKDSPLDVIAINDTGGVKQASHLLKYDSTLGIFDADVKAVGDTVISVDGKEIQVVSNRNPANLPWKELGIDLVIEGTGVFVDREGAGKHIQAGAKKVLITAPGKGDIPTYVVGVNADAYSPDEPIISNASCTTNCLAPFVKVLDQKFGIIKGTMTTTHSYTGDQRLLDASHRDLRRARAAALNIVPTSTGAAKAVALVLPSLKGKLNGIALRVPTPNVSVVDLVVQVEKKTFAEEVNAAFRESADKELNGILSVCDEPLVSVDFRCSDVSSTVDASLTMVMGDDMVKVIAWYDNEWGYSQRVVDLADIVANQWK

>GAPDHA iso8

MAWEEGFTSHLLKYDSTLGIFDADVKPVGDTVISVDGKEIQVVSNRNPANLPWKELGIDLVIEGTGVFVDREGAGKHIQAGAKKVLITAPGKGDIPTYVVGVNADAYNPDEPIISNASCTTNCLAPFVKVLDQKFGIIKGTMTTTHSYTGDQRLLDASHRDLRRARAAALNIVPTSTGAAKAVALVLPSLKGKLNGIALRVPTPNVSVVDLVVQVEKKTFAEEVNAAFRESAEKELNGILSVCDEPLVSVDFRCTDVSSTVDASLTMVMGDDMVKVIAWYDNEWGYSQRVVDLADIVANQWK

>GAPDHB iso1

MVVAAMASAHAALASSRLPSGNRLTSRANHAHATQCLSKRLDLADFSGLRSSGCVTFSKQGREQSFHDVLAAQLTTKATGTPVKGETVAKLKVAINGFGRIGRNFLRCWHGRKDSPLEVIVVNDSGGVKNASHLLKYDSMLGTFKADVKIVDNETISVDGKPVKVVSSRDPLKLPWAELGIDIVIEGTGVFVDGPGAGKHIQAGAKKVIITAPAKGADIPTYVVGVNEGDYGHEVANIISNASCTTNCLAPFVKILDEEFGIVKGTMTTTHSYTGDQRLLDASHRDLRRARAAALSIVPTSTGAAKAVSLVLPQLKGKLNGIALRVPTPNVSVVDLVVNVEKKGISADDVNAAFRKAAEGRLKGVLAVCDVPLVSVDFRCTDVSSTIDSSLTMVMGDDMVKVVAWYDNEWGYSQRVVDLAHLVAEKWPGAPVKRPGDPLEDYCETNPETKECKVFEA

>GAPDHB iso2

MASAHAALASSRLPSGTRLTSRANHAHATQCLSKRLDLADFSGLRSSGCVTFSKQGREQTFHDALAAQLTTKATGTPVKGETVAKLKVAINGFGRIGRNFLRCWHGRKDSPLEVIVVNDSGGVKNASHLLKYDSMLGTFKADVKIVDNETISVDGKPVKVVSSRDPLKLPWAELGIDIVIEGTGVFVDGPGAGKHIQAGAKKVIITAPAKGADIPTYVVGVNEGDYGHEVANIISNASCTTNCLAPFVKILDEEFGIVKGTMTTTHSYTGDQRLLDASHRDLRRARAAALSIVPTSTGAAKAVSLVLPQLKGKLNGIALRVPTPNVSVVDLVVNVQKKGISADDVNAAFRKAAEGRLKGVLAVCDVPLVSVDFRCTDVSSTIDSSLTMVMGDDMVKVVAWYDNEWGYSQRVVDLAHLVAEKWPGAPVKRPGDPLEDYCETNPETKECKVFEE

>GAPDHB iso3

MLGTFKADVKIVDNETISVDGKPVKVVSSRDPLKLPWAELGIDIVIEGTGVFVDGPGAGKHIQAGAKKVIITAPAKGADIPTYVVGVNEGDYGHEVANIISNASCTTNCLAPFVKILDEEFGIVKGTMTTTHSYTGDQRLLDASHRDLRRARAAALSIVPTSTGAAKAVSLVLPQLKGKLNGIALRVPTPNVSVVDLVVNVQKKGISADDVNAAFRKAAEGRLKGVLAVCDVPLVSVDFRCTDVSSTIDSSLTMVMGDDMVKVVAWYDNEWGYSQRVVDLAHLVAEKWPGAPVIRPGDPLEDYCETNPETKECKVFEE

>GAPDHB iso4

MASAHAALASSRLPSGTRLTSRANHAHATQCLSKRLDLADFSGLRSSGCVTFSKQGREQSFHDVLAAQLTTKATGTPVKGETVAKLKVAINGFGRIGRNFLRCWHGRKDSPLEVIVVNDSGGVKNASHLLKYDSMLGTFKADVKIVDNETISVDGKPVKVVSSRDPLKLPWAELGIDIVIEGTGVFVDGPGAGKHIQAGAKKVIITAPAKGADIPTYVVGVNEGDYGHEVANIISNASCTTNCLAPFVKILDEEFGIVKGTMTTTHSYTGDQRLLDASHRDLRRARAAALSIVPTSTGAAKAVSLVLPQLKGKLNGIALRVPTPNVSVVDLVVNVQKKGISADDVNAAFRKAAEGRLKGVLAVCDVPLVSVDFRCTDVSSTIDSSLTMVMGDDMVKVVAWYDNEWGYSQRVVDLAHLVAEKWPGAPVKRPGDPLEDYCETNPETKECKVFEE

>GAPDHB iso5

MVVAAMASAHAALASSRLPSSNRLTSRANHAHATQCLSKRLDLADFSGLRSSGCVTFSKQGREQSFHDVLAAQLTTKATGTPVKGETVAKLKVAINGFGRIGRNFLRCWHGRKDSPLEVIVVNDSGGVKNASHLLKYDSMLGTFKADVKIVDNETISVDGKPVKVVSSRDPLKLPWAELGIDIVIEGTGVFVDGPGAGKHIQAGAKKVIITAPAKGADIPTYVVGVNEGDYSHEVANIISNASCTTNCLAPFVKILDEEFGIVKGTMTTTHSYTGDQRLLDASHRDLRRARAAALSIVPTSTGAAKAVSLVLPQLKGKLNGIALRVPTPNVSVVDLVVNVQKKGISADDVNAAFRKAAEGRLKGVLAVCDVPLVSVDFRCTDVSSTIDSSLTMVMGDDMVKVVAWYDNEWGYSQRVVDLAHLVAEKWPGAPVIRPGDPLEDYCETNPETKECKVFEE

>AlaAT iso1

MAGLITLDNINPKVKECEYAVRGAIVTHAQRLQQQLQSNPASLPFDEILYCNIGNPQSLGQEPITFYREVLALLDHPALLQKEEALSLFSSDAIERAQQILAQIPGRATGAYSHSQGIKGLRDAIAAGITARDGYPANADDIFLTDGASPGVHTMMRLLIRSKNDGILCPIPQYPLYSASIALHGGTLVPYYLNEEAGWGLELPEVKNQLEAARSNGIEVRALVVINPGNPTGQVLSKENQIEIVDFCKKEGLVLLADEVYQQNVYAKDKEFNSFKKISRSMGYGDEDITLVSFQSVSKGFYGECGKRGGYMEVTGFDAGVREQIYKLASVNLCSNISGQILASLVMNPPKEGDKSYASYAAERDGILSSLARRAKILVDAFNSMEGITCNNAEGAMYLFPRIILPNKAIEAAKAANTSPDAFYAHRLLDATGIVVVPGSGFRQVPGTYHFRCTILPPESKMPLIASRFKAFHEAFIAEFHD

>AlaAT iso2

MRRFVADRAKKLFFYSISADSSRIGPLFSRLLTSSSVEAATMSPTVTVDTNRVPAVTVDTINPKVLKCEYAVRGEIVSLAQHLQTELQAKPGSHPFDEILYCNIGNPQSLGQQPITFFREILALVDHPALLDKDGTHALFSADSIDRAFKILERIPGRATGAYSHSQGIKGLRDEIAAGIASRDGYAANADDIFLTDGASPAVHMMMQLLIRSEKDGILCPIPQYPLYSASIALHGGSLVPYYLDEATGWGLELSEVKKQLEEAKSKGITVRALVVINPGNPTGQVLAEDNQKEIVEFCKNEGLVLLADEVYQENVYVEDKKFNSFKKVSRSMGYGDEDLALVSFQSVSKGYYGECGKRGGYMEVTGFSADVREQIYKVASVNLCSNISGQILASLVMNPPQVGDESYESYTAEKEEILSSLARRAKTLEDAFNSMEGVTCNKAEGAMYLFPRLCLPNKAIEAAKAAKAAPDAFYARRLLNATGIVVVPGSGFGQVPGTWHIRCTILPQEDKIPGIVSSLKAFHEAFMAEFRD

>AlaAT iso3

MRRFVADRAKRLLFPSTSADPSRIGPLFSRLLTSSGEQAATMSPTATVDTNRVPAVTVDTINPKVLKCEYAVRGEIVSLAQHLQTELQAKPGSHPFDEILYCNIGNPQSLGQQPITFFREILALVDHPALLDKDGTHALFSADSIDRAFKILERIPGRATGAYSHSQGIKGLRDEIAAGITARDGYAANADDIFLTDGASPAVHMMMQLLIRSEKDGILCPIPQYPLYSASIALHGGSLVPYYLDEATGWGLELSEVKKQLEEAKSKGITVRALVVINPGNPTGQVLAEDNQKEIVEFCKNEGLVLLADEVYQENVYVKDKKFNSFKKVSRSMGYGDEDLALVSFQSVSKGYYGECGKRGGYMEVTGFSADVREQIYKVASVNLCSNISGQILASLVMNPPQVGDESYESYTAEKEEILSSLARRAKTLEDAFNSMEGVTCNKAEGAMYLFPRLRLPNKAIEAAKAAKAAPDAFYARRLLNATGIVVVPGSGFGQVPGTWHIRCTILPQEDKIPGIVSSLKAFHEAFMAEFRD

>AspAT iso1

MASGSLLRRTILRPSISPAIGGARSVTSWWSNVEPAAKDPILGVTEAFLADTNPNKVNVGVGAYRDDNGKPVVLDCVREAERRIAGNQNMEYLPMGGSMKLIEESIKLAYGENSEFIKDKRVAAVQTLSGTGACRLFAEFQRRFRSDSQIYISVPTWANHHNIWRDAHVPQRTFHYYHAETRALDFAALMDDVKNAPGGSFFLLHACAHNPTGVDPSEEQWREISYQFKVKQHFAFFDMAYQGFASGDPERDAKAIRIFLEDGHLIGCAQSFAKNMGLYGQRVGCLSVLCEDELQAVAVKSQLQQIARPMYSNPPLHGALVVSIILADPELKLLWLKEVKGMADRIIGMRAALRDNLEALGSPLSWEHITNQIGMFCYSGLTPEQVDWLTNEYHIYMTRNGRISMAGVTTGNVGYLANAIHEVTKSR

>AspAT iso2

RYYDPATRGLDFQGLIEDLSAAPSGAIVLLHACAHNPTGVDPTPDQWEQIRQLMRSKALLPFFDSAYQGFASGSLDADAYSVRLFVSDGGECLAAQSYAKNLGLYGERVGALSIVCRAADVASRVESQLKLVIRPMYSNPPIHGASIVATILRDRQMFTDWTVELKAMADRIISMRKQLYEALLAKGTPGDWTHIIKQIGMFTFTGLNTQQVAFMTKEYHIYMTSDGRISMAGLSAQRIPHLVEAIHAAVTRVA

>AspAT iso3

KSQLKRLARPMYSNPPVHGARIVANVVGDPVLFQEWKDEMAMMAGRIKNVRQRLYDSLTEKDKSGKDWSFILKQIGMFSFTGLNKSQSDNMTGKWHVYMTKDGRISLAGLSLAKCEYLADAIIDSFHNVS

>AspAT iso4

MASTMVSLSFSAAVARGTENARVGHAAWKKKRNGFANMKSPARTTMSVAVDVSRFEGVSMAPPDPILGVSEAFRADTNDLKLNLGVGAYRTEELQPYVLNVVKKAEKLMLEKGENKEYLPIEGLAAFNNATADLLLGADNPVIQQGRIATVQGLSGTGSLRLGAAFIQRYFPEAKALISSPTWGNHKNIFNDARVPWSEYRYYDPRTVGLDFEGMIADIKAAPEGSFVVLHGCAHNPTGIDPSPEQWEKIADVIQEKNHVPFFDVAYQGFASGSLDDDAYSVRMFVSRGLEALVAQSYSKNLGLYSERIGAINVVCSSSDAAIRVKSQLKRLARPMYSNPPVHGARIVANVVGDPVLFQEWKDEMVMMAGRIKNVRQRLYDSLTEKEKSGKDWSFILKQIGMFSFTGLNKSQSDNMTDKWHVYMTKDGRISLAGLSLAKCEYLADAIIDSFHNVS

>DTC

MGDSKPKSAAPSGVWATVKPFANGGASGMLATCVIQPIDMVKVRIQLGQGSAGHVAKTMLANDGITAFYKGLSAGLLRQATYTTARLGSFRVLTNKAVEANDGKPLPLLQKAVIGLTAGAIGACVGSPADLALIRMQADATLPLAQRRHYKNAFHALYRIVADEGVLALWKGAGPTVVRAMSLNMGMLASYDQSIEFFRDSLGLGEYSTVVGASAVSGFFASACSLPFDYVKTQIQKMQPDASGKYPYTGSLDCFVKTVKSGGPFKLYTGFPVYCVRIAPHVMMTWIFLNQIQKFEKKIGL

>MPC1 iso1

MSAFRAFLNSPVGPKTTHFWGPVANWGFVVAGLVDTQKPPDMISGNMTAAMCVYSGLFMRFAWMVQPRNYLLLACHASNETVQLYQLSRWEKSQGYIGKTGATEGH

>MPC1 iso2

MSAFRAFLNSPVGPKTTHFWGPVANWGFVVAGLVDTQKPPDMISGNMTAAMCVYSGLFMRFAWMVQPRNYLLLACHASNETVQLYQLSRWAKGQGYIGKTEATEGH

>MPC1 iso3

MLQMKQSNFTNFHVGQKARGTLGRRSPLRVIDFFLFYVVFIFLECYNAPDHILPLSWIDSWIACGRLSSNLMISFINCVSPRKTIPCYHPYACFINKRNFVVTHILHCFISSPFDVRC

>MPC1 iso4

MKLSNFTNFHVGQKARGTLGRQRPLRGIDFFLFSVVLVFLECYNAPDHILPLSWIDSWIARGRLSSNLRVFGINCVSPRKTIPCYHPYVCFINKRNFAVTCNTLFYLISFRRMCQTLELNSLYQSECSIDS

>MPC1 iso5

MSAFRAFLNSPVGPKTTHFWGPVANWGFVVAGLVDTQKPPDMISGNMTAAMCVYSGLFMRFAWMVQPRNYLLLACHASNETVQLYQLSRWAKGQGYIGKMEATEGH

>MPC1 iso6

MSAFRAFLNSPVGPKTTHFWGPVANWGFVVAGLVDTQKPPDMISGNMTAAMCVYSGLFMRFAWMVQPRNYLLLACHASNETVQLYQLSRWAKGQGYIGKTEATEGH

>MPC1 iso7

MSAFRAFLNSPVGPKTTHFWGPVANWGFVVAGLVDTQKPPDMISGNMTAAMCVYSGLFMRFAWMVQPRNYLLLACHASNETVQLYQLSRWAKGQGYIGKMEATEGH

>MPC1 iso8

MSAFRAFLNSPVGPKTTHFWGPVANWGFVVAGLVDTQKPPDMISGNMTAAMCVYSGLFMRFAWMVQPRNYLLLACHASNETVQLYQLSRWAKGQGYIGKMEATEGH

**Table S2** **Phosphorylation state of PEPC isoforms at different times.** [See separate Excel file.]

**Table S3.** **Comparison of biochemical CO2 concentrating mechanisms in leaves of aquatic and terrestrial plants. CAM stands for Crassulacean Acid Metabolism, NAD(P)-ME stands for NAD(P) malic enzyme, PEP-CK stands for phosphoenolpyruvate-carboxykinase.**

**References**

**Casati P, Lara MV, Andreo CS. 2000.** Induction of a C_4_-like mechanism of CO_2_ fixation in *Egeria densa*, a submersed aquatic species. *Plant Physiology* **123**: 1611-1622.

**Das VSR and Raghavendra AS. 1977.** Kranz leaf anatomy and C_4_ dicarboxylic acid pathway of photosynthesis in *Spinifex squarrosus* L. *Indian Journal of Experimental Biology* **15**: 645–648.

**Gilman IS, Moreno-Villena JJ, Lewis ZR, Goolsby EW, Edwards EJ. 2022.** Gene co-expression reveals the modularity and integration of C_4_ and CAM in *Portulaca*. *Plant Physiology* **189**: 735-753.

**Guralnick LJ, Edwards G, Ku MSB, Hockema B, Franceschi V. 2002.** Photosynthetic and anatomical characteristics in the C_4_ crassulacean acid metabolism-cycling plant *Portulaca grandiflora*. *Functional Plant Biology* **29**: 763-773.

**Ho C-L, Chiang J-M, Lin T-C, Martin CE. 2019.** First report of C_4_/CAM-cycling photosynthetic pathway in a succulent grass, *Spinifex littoreus* (Brum. f.) Merr., in coastal regions of Taiwan. *Flora* **254**: 194-202.

**Huang J, Khan S, Wang S, Liao Z, Zhu X, Han Q, W Li, Yin L, Jiang HS. 2025.** C_4_-like metabolism and HCO_3_^-^ use in submerged leaves of *Ottelia cordata* lacking Kranz anatomy at both low and high CO_2_ concentrations. *Plant Physiology and Biochemistry* **219**: 109346.

**Koch K., Kennedy RA. 1980.** Characteristics of crassulacean acid metabolism in the succulent C_4_ dicot, *Portulaca oleracea* L. *Plant Physiology* **65**: 193-197.

**Lara MV, Casati P, Andreo CS. 2002.** CO_2_-concentrating mechanisms in *Egeria densa*, a submersed aquatic plant. *Plant Physiology* **115**: 487-495.

**Magnin NC, Cooley BA, Reiskind JB, Bowes G. 1997.** Regulation and localization of key enzymes during the induction of kranz-less, C_4_-type photosynthesis in *Hydrilla verticillata*. *Plant Physiology* **115**: 1681-1689.

**Moreno-Villena JJ, Zhou H, Gilman IS, Tausta SL, Cheung CYM, Edwards EJ. 2022.** Spatial resolution of an integrated C_4_+CAM photosynthetic metabolism. *Science Advances* **8**: eabn2349.

**Muhaidat R. McKown AD. 2013.** Significant involvement of PEP-CK in carbon assimilation of C_4_ eudicots. *Annals of Botany* **111**: 577-589.

**Rao SK, Magnin NC, Reiskind JB, Bowes G. 2002.** Photosynthetic and other phosphoenolpyruvate carboxylase isoforms in the single-cell, facultative C_4_ system of *Hydrilla verticillata*. *Plant Physiology* **130**: 876-886.

**Reiskind JB, Madsen TV, Van Ginkel LC, Bowes G. 1997.** Evidence that inducible C_4_-type photosynthesis is a chloroplastic CO_2_-concentrating mechanism in *Hydrilla*, a submersed monocot. *Plant, Cell and Environment* **20**: 211-220.

**Siadjeu C, Kadereit G. 2024.** C_4_-like *Sesuvium sesuvioides* (Aizoaceae) exhibits CAM in cotyledons and putative C_4_-like + CAM metabolism in adult leaves as revealed by transcriptome analysis. *BMC Genomics* **25**: 688.

**Voznesenskaya EV, Franceschi VR, Kiirats O, Freitag H, Edwards GE. 2001.** Kranz anatomy is not essential for terrestrial C_4_ plant photosynthesis. *Nature* **414**: 543-546.

**Voznesenskaya EV, Koteyeva NK, Edwards GE, Ocampo G. 2017.** Unique photosynthetic phenotypes in *Portulaca* (Portulacaceae): C_3_-C_4_ intermediates and NAD-ME C_4_ species with Pilosoid-type Kranz anatomy. *Journal of Experimental Biology* **68**: 225-239.

**Wang S, Li P, Liao Z, Wang W, Chen T, Yin L, Jiang HS, Li W. 2022.** Adaptation of inorganic carbon utilization strategies in submerged and floating leaves of heteroblastic plant *Ottelia cordata*. *Environmental and Experimental Botany* **196**: 104818.

**Winter K, Garcia M, Virgo A, Holtum JAM.** **2019.** Operating at the very low wend of the Crassulacean acid metabolism spectrum*: Sesuvium portulacastrum* (Aizoaceae). *Journal of Experimental Botany* **70**: 6561-6570.

**Winter K, Garcia M, Virgo A, Ceballos J, Holtum JAM. 2021.** Does the C_4_ plant *Trianthema portulacastrum* (Aizoaceae) exhibit weakly expressed crassulacean acid metabolism (CAM)? *Functional Plant Biology* **48**: 655-665.
